# Electrochemical Proton Reduction over Nickel Foam for *Z*‐Stereoselective Semihydrogenation/deuteration of Functionalized Alkynes

**DOI:** 10.1002/cssc.202102221

**Published:** 2021-12-16

**Authors:** Alejandro Valiente, Pablo Martínez‐Pardo, Gurpreet Kaur, Magnus J. Johansson, Belén Martín‐Matute

**Affiliations:** ^1^ Department of Organic Chemistry Stockholm University The Arrhenius Laboratory 16C 106 91 Stockholm Sweden; ^2^ Medicinal Chemistry, Research and Early Development; Cardiovascular, Renal and Metabolism (CVRM) Biopharmaceuticals R&D AstraZeneca Pepparedsleden 1 43150 Mölndal, Gothenburg Sweden

**Keywords:** alkynes, electrocatalysis, nickel foam, semideuteration, semihydrogenation

## Abstract

Selective reduction strategies based on abundant‐metal catalysts are very important in the production of chemicals. In this paper, a method for the electrochemical semihydrogenation and semideuteration of alkynes to form *Z*‐alkenes was developed, using a simple nickel foam as catalyst and H_3_O^+^ or D_3_O^+^ as sources of hydrogen or deuterium. Good yields and excellent stereoselectivities (*Z*/*E* up to 20 : 1) were obtained under very mild reaction conditions. The reaction proceeded with terminal and nonterminal alkynes, and also with alkynes containing easily reducible functional groups, such as carbonyl groups, as well as aryl chlorides, bromides, and even iodides. The nickel‐foam electrocatalyst could be recycled up to 14 times without any change in its catalytic properties.

## Graphical abstract

**Foam catalyst**: Commercially available nickel foam is an excellent catalyst for the electrochemical semihydrogenation/semideuteration of functionalized alkynes in diluted sulfuric acid under ambient conditions. Here, the applicability of this catalyst for the stereoselective synthesis of *Z*‐alkenes and *Z*‐alkenes‐*d_2_* is presented. The nickel foam is recyclable up to 14 times without losing its catalytic properties.

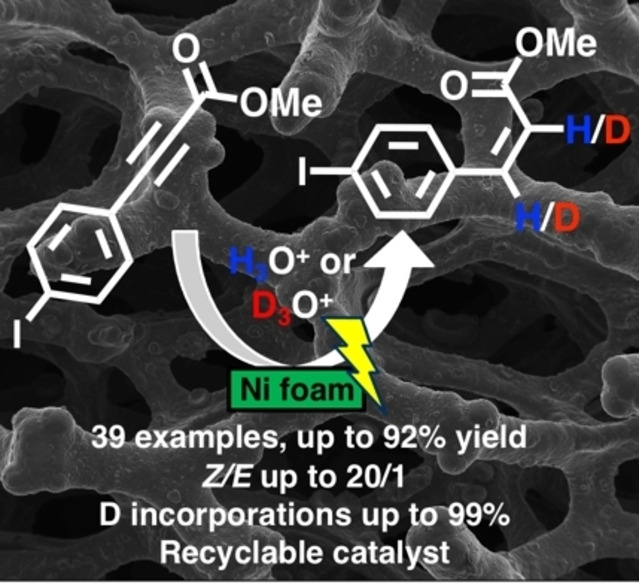

## Introduction

The stereoselective synthesis of alkenes is a highly relevant reaction in synthetic organic chemistry. It gives selective access to *Z*‐alkenes, which have applications in materials chemistry as well as in the pharmaceutical industry.[^1^, ^2^, ^3^, ^4^, ^5^] The semireduction of alkynes is one of the few available methods for the formation of *Z*‐alkenes. This reaction mainly uses palladium[^6^, ^7^] (e. g., Lindlar),^[8]^ rhodium,[^9^, ^10^, ^11^] ruthenium,^[12]^ or iridium[^13^, ^14^, ^15^] catalysts in gas cylinders/pressurized reaction vessels.^[2]^ The use of catalysts based on earth‐abundant metals rather than noble metals for hydrogenation reactions is an interesting prospect as it would contribute to sustainable organic synthesis. Raney nickel is a common example of a highly active base‐metal catalyst for reduction reactions; however, its pyrophoric nature is a significant limitation.^[16]^ Other nickel complexes or nanoparticles have been reported to be good reduction catalysts, sometimes needing high hydrogen pressure and high temperature,[^17^, ^18^, ^19^, ^20^, ^21^] or an excess of the reductants, such as NaBH_4_ or formic acid.[^22^, ^23^, ^24^, ^25^, ^26^] Hydrogen obtained from fossil resources can also be replaced by hydrogen produced electrochemically from water.[^5^, ^27^, ^28^, ^29^, ^30^] This approach is particularly important for deuteration reactions, as it allows isotopically labeled compounds, which have applications in medicinal chemistry, to be synthesized simply by using deuterated water solutions, without the need to use deuterium gas cylinders.[^31^, ^32^] To make use of the hydrogen produced electrochemically, an additional catalyst (namely, the hydrogenation catalyst) is needed. Recently, examples where a single metal fulfills both functions have been reported, using copper catalysts[^33^, ^34^] or a selenium‐doped nickel‐foam electrode.^[35]^ The former are excellent catalysts for the reduction of acetylene gas, and the latter, under basic conditions (KOH, 1 m), gives excellent results in the reduction of terminal alkynes. It would be desirable to use these same materials for the synthesis of functionalized *Z*‐alkenes, but this remains a challenging goal. Different functionalized nickel foams are well‐known electrocatalysts for the hydrogen‐evolution reaction (HER) under both basic and acidic conditions.[^36^, ^37^, ^38^, ^39^, ^40^] Specifically, the electrochemical performance of the Ni foam/sulfuric acid system has been widely investigated, but to the best of our knowledge it has barely been explored in organic synthesis.^[39]^

**Scheme 1:**
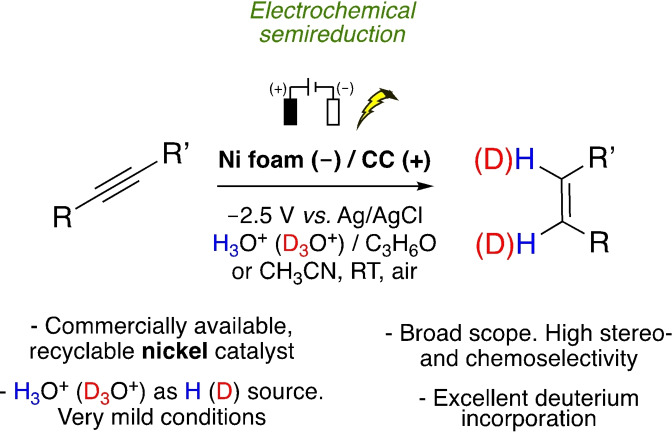
This work: electrocatalytic semireduction of alkynes over a nickel foam.

In this paper, we report a straightforward procedure for the electrochemical *Z‐*selective semireduction of substituted alkynes with commercial nickel foam as the cathode, and carbon cloth (CC) as the anode, using H_3_O^+^ or D_3_O^+^ as hydrogen or deuterium sources, under very mild conditions (Scheme 1). A wide variety of internal and terminal alkynes were hydrogenated and deuterated in short reaction times with excellent stereoselectivities. The reaction works well for terminal and nonterminal alkynes, and it is highly chemoselective, as demonstrated by the reduction of alkynes functionalized with reducible groups such as ketones, cyano groups, and aromatic halides. The nickel cathode showed excellent recyclability of up to at least 14 cycles.

## Results and Discussion

We started our investigations using diphenylacetylene (**1 a**) as a model substrate in a mixture of aqueous sulfuric acid and acetone (*v*/*v*=1 : 1) as electrolyte. The initial tests were carried out at a potential such that enough catalytically active species would be present for the semihydrogenation reaction to take place. Thus, **1 a** was subjected to −1.5 V vs. Ag/AgCl for 2 h (Table 1, entry 1), which gave a 31 % yield of *Z‐*stilbene (**2 a**) along with 3 % of *E‐*stilbene (**3 a**) and 10 % of 1,2‐diphenylethane (**4 a**). When the reaction time was extended to 6 h under these same conditions, **2 a** was formed in 48 % yield, albeit with large amounts of the overhydrogenation product **4 a** (32 %, Table 1, entry 2). Different ratios of acetone and water were tested (1 : 3 or 3 : 1, Table 1, entries 3 and 4, respectively), but the outcome did not improve. Other solvents were tested (Figure S1), and we found that acetone gave the best initial results. This could be due to a combination of different factors, namely coordination properties of the solvent, or solubility of **2 a** in the mixture of organic solvent with aqueous sulfuric acid (*v*/*v*=1 : 1). The best solubility was found when acetone was used as the organic solvent (Figure S1). When the concentration of sulfuric acid was lowered from 0.25 to 0.13 m, 29 % of **2 a** was obtained, and only 2 % of alkane **4 a** (5 times less than in Table 1, entry 1 over the same reaction time). Further decreasing the concentration of sulfuric acid (0.05 m, Table 1, entry 6), resulted in a severe decrease in the yield. A plausible explanation could be that H_3_O^+^ is the actual source of catalytically active species, and therefore lowering its concentration below certain threshold has a dramatic effect in the yield. When NBu_4_BF_4_ was used instead of H_2_SO_4_ (Table 1, entry 7), only traces of **2 a** were formed, supporting this hypothesis.

**Table 1:** Optimization of the reaction conditions.^[a]^

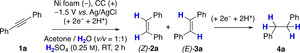				
Entry	Deviation from initial conditions	Conv. [%]	Yield **2 a**/**3 a**/**4 a** [%]	*Z/E*
1	none	44	31 : 3 : 10	10 : 1
2	6 h	87	48 : 7 : 32	7 : 1
3^[b]^	acetone/H_2_O, 1 : 3, *v*/*v*	18	14 : 2 : 2	7 : 1
4	acetone/H_2_O, 3 : 1, *v*/*v*	9	8 : 1:–	5 : 1
5	H_2_SO_4_ (0.13 m)	33	29 : 2 : 2	15 : 1
6	H_2_SO_4_ (0.05 m)	11	8 : 2 : 1	4 : 1
7	NBu_4_BF_4_ (0.25 m)	–	traces	–
8	H_2_SO_4_ (0.13 m), −2 V	59	51 : 3 : 5	19 : 1
9	H_2_SO_4_ (0.13 m), −2.5 V	78	64 : 4 : 10	16 : 1
**10**	**H_2_SO_4_ (0.13 m), −2.5 V, 4 h**	**94**	**76 : 4 : 13**	**19 : 1**

[a] Reactions were carried out in a divided cell using **1 a** (0.4 mmol) in of solvent mixture (25 mL in each chamber). Conversion, yields, and *Z/E* ratios were determined by ^1^H NMR spectroscopy using an internal standard. [b] **1 a** was poorly soluble.

When the negative potential was increased to −2.0 or −2.5 V in sulfuric acid (0.13 m), **2 a** was obtained in 51 and 64 % yield, respectively, within 2 h (Table 1, entries 8 and 9). When the reaction time was increased to 4 h, *Z‐*alkene **2 a** was obtained in 76 % yield with a Z/*E* selectivity of 19 : 1, together with just 13 % of overhydrogenated **4 a** (Table 1, entry 10). Longer reaction times resulted in the consumption of product **2 a** to form **4 a** (Figure S2).

**Scheme 2:**
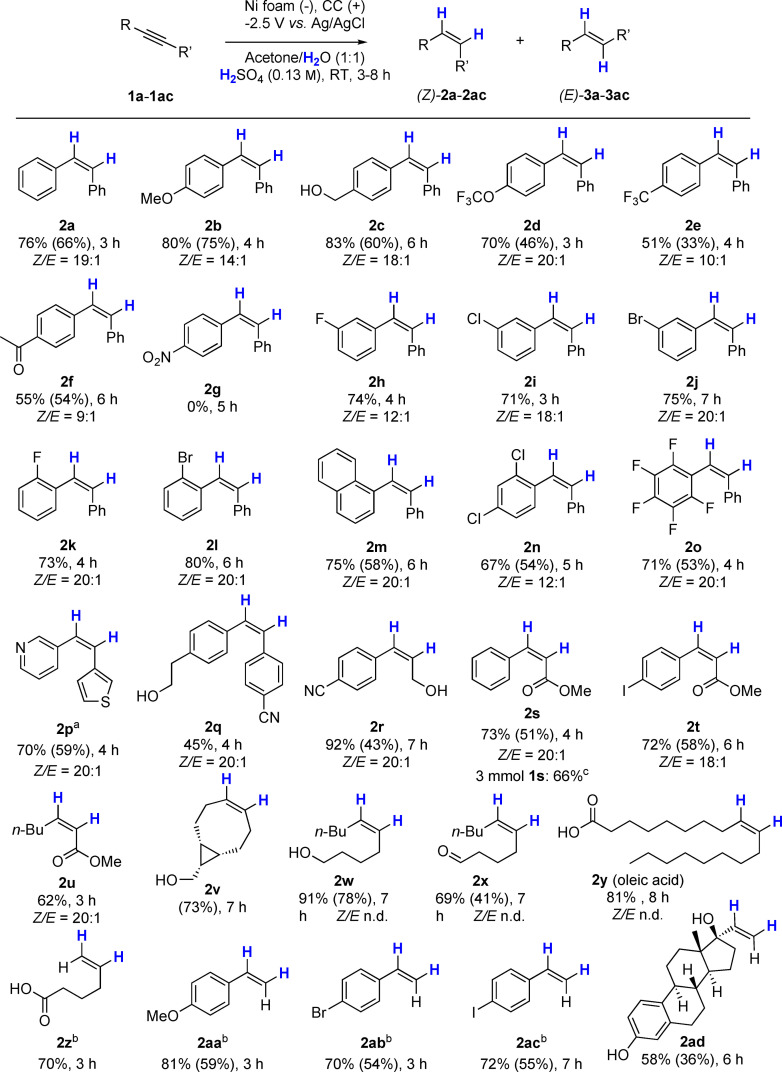
Substrate scope. Unless otherwise indicated: alkyne (0.4 mmol), acetone/H_2_O (25 mL; 1 : 1, *v*/*v*), H_2_SO_4_ (0.13 m), Ni foam cathode (1 cm^2^). [a] H_2_SO_4_ (0.25 m); [b] H_2_SO_4_ (0.06 m); [c] 3 mmol of **1 s**, H_2_SO_4_ (1 m), 7 h. Yields and *Z*/*E* ratios were determined by ^1^H NMR spectroscopy using an internal standard. Isolated yields in parentheses.

The scope and limitations of the electrochemical semihydrogenation reaction on Ni foam was then investigated (Scheme 2) under the optimized conditions (Table 1, entry 10). Substituted diphenylacetylene derivatives bearing electron‐donating groups at the *para‐*position on one of the aryl groups gave *Z‐*alkenes **2 b** (*p‐*OCH_3_) and **2 c** (*p‐*CH_2_OH) in 80 and 83 % yield, respectively, with high stereoselectivity (*Z*/*E* up to 18 : 1). Fluorinated derivatives **1 d** (*p‐*OCF_3_) and **1 e** (*p‐*CF_3_) were reduced in 70 and 51 % yields. Importantly, acylated diphenylacetylene (**1 f**) was reduced chemoselectively to give **2 f** in 55 % yield, the mass balance being the saturated product **4 f**. Importantly, the ketone group remained untouched. In the case of the NO_2_‐containing **1 g**, only the cathodic reduction of the NO_2_ group took place, and **2 g** was not detected (Figure S3). F‐substituted diphenylacetylene **1 h** gave **2 h** in 74 % yield (*Z*/*E*=12 : 1). Halides such as Cl and Br in **1 i** and **1 j** were very well tolerated, and the corresponding *Z‐*alkenes were formed in yields of 71 and 75 %, respectively. Other substrates with *ortho‐*substituents, such as **1 k** (*o‐*F) and the bulkier *o‐*Br (**1 l**) gave the corresponding *Z‐*alkenes in good yields. Naphthalene derivative **2 m** was obtained in 75 % yield with a *Z*/*E* ratio of 20 : 1. Reduction of polychlorinated and fluorinated compounds **1 n** and **1 o** was achieved in 67 and 71 % yields, respectively (*Z*/*E*≥12 : 1). The efficiency of the system for the reduction of heterocycle‐containing alkynes was tested by reducing 3‐(thiophen‐3‐ylethynyl)pyridine (**1 p**), which gave a 70 % yield with a *Z*/*E* ratio of 20 : 1. Polysubstituted derivative **2 q** was obtained in a modest yield (45 %), whereas propargylic alcohol **1 r** was successfully reduced to (*Z*)‐4‐(3‐hydroxyprop‐1‐en‐1‐yl)benzonitrile (**2 r**) in excellent yield and with excellent selectivity (92 %, *Z*/*E*=20 : 1). The cyano group remained untouched in both of these examples, thus this approach can be used to give access to aromatic building blocks bearing both nitrile and allylic alcohol functionalities, which can serve as handles for further functionalization. Ester‐substituted alkynes (R′=CO_2_Me) **1 s** and **1 t** were tolerated, giving **2 s** and **2 t** in 73 and 72 % yields, respectively, with *Z*/*E* ratios of 20 : 1. Importantly, dehalogenation did not occur in the reduction of 4‐iodopheyl acrylate (**1 t**), which is a common cause of byproduct formation when using Pd catalysts.^[41]^ To the best of our knowledge, there are only a few semireduction methods in the literature that are compatible with aryl iodides.[^42^, ^43^] The semihydrogenation of **1 s** was also performed at 3 mmol scale, obtaining **2 s** in 66 % yield (Scheme 2 and Figure S4). Aliphatic ester **2 u** was formed in 62 % yield (*Z*/*E*=20 : 1) in only 3 h from the corresponding alkyne. The method also works well on alkynes with aliphatic substituents at both R and R′, as exemplified by **2 v**–**2 y**. (*Z*)‐Bicyclo[6.1.0]non‐4‐en‐9‐ylmethanol (**2 v**) was produced in 58 % yield after selective hydrogenation of the strained cyclooctyne. Functionalized cyclooctynes have proved to be useful compounds in chemical biology, but access to the corresponding cyclooctene derivatives is usually more difficult. The approach described here would provide precursors for Diels‐Alder reactions, giving access to functionalized cycloaddition adducts.^[44]^ (*Z*)‐Dec‐5‐en‐1‐ol (**2 w**) was obtained in excellent yield (91 %) within 7 h. Remarkably, aliphatic aldehydes such as dec‐5‐ynal (**1 x**) were chemoselectively reduced affording **2 x** in 69 % yield along with only 15 % of the alcohol side‐product (**2 w**). Unprotected stearolic acid was also reduced to oleic acid (**2 y**) in 81 % yield. In general, terminal aliphatic alkynes are reduced easily under these conditions, as exemplified by **1 z**, **1 aa**, **1 ab**, **1 ac**, and **1 ad**. The more complex molecule ethynylestradiol (**1 ad**) was also reduced to give **2 ad** in a synthetically useful yield of 58 %. A current efficiency of 8.6 % was estimated for **2 a** under the reaction conditions of Table 1, entry 10 (Figure S5). The efficiencies are higher (up to ≈30 %) for terminal alkynes, which are more prone to be reduced than internal alkynes under the reaction conditions reported here (Figure S6). The hydrogen evolution reaction occurs simultaneously, resulting in lower current efficiencies for substrates that require longer reaction times. Despite this, the simplicity of the method, the availability of this simple Ni foam and the good isolated chemical yields make this method useful for organic synthesis.

H−D isosteric replacement is used in medicinal chemistry to improve the metabolic stability of drugs (e. g., facile access to deuterated alkenes will allow for the stereo‐/enantioselective synthesis of functionalized cyclopropanes of high value in drug discovery programs).^[45]^ For the synthesis of deuterated alkenes, deuterium gas (D_2_) and a metal catalyst (normally, palladium), or superstoichiometric amounts of deuterated reductants are traditionally used.[^30^, ^44^, ^45^, ^46^, ^47^] Electrochemical approaches allow readily available deuterated water/acids to be used as the deuterium sources. We were able to expand the method to allow the deuteration of internal and terminal alkynes by slightly altering the reaction conditions; deuterated water and deuterated sulfuric acid were used, and acetone (p*K*_a_=20) was replaced by acetonitrile (p*K*_a_=25), as acetone was found to engage in H/D scrambling reactions (Figure S7).

**Scheme 3:**
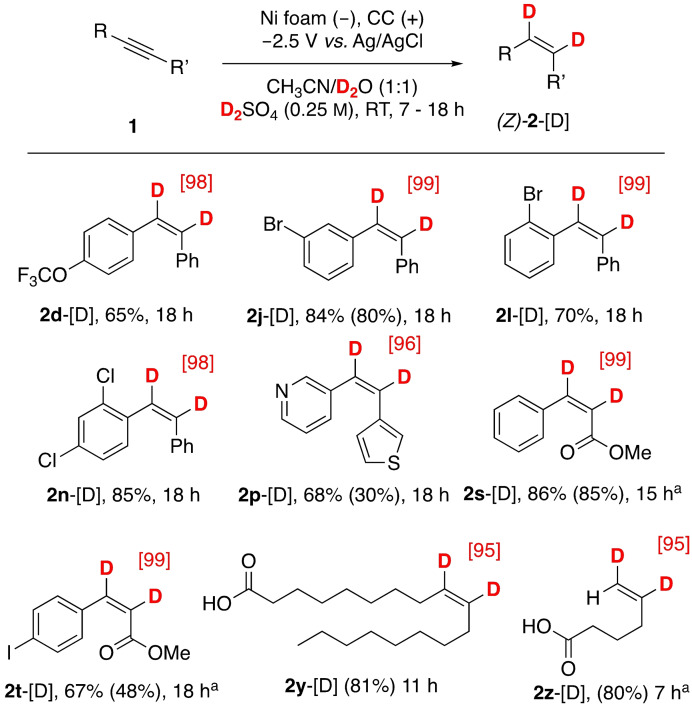
Semideuteration of alkynes. Unless otherwise indicated: alkyne (0.4 mmol), CH_3_CN/H_2_O (25 mL; 1 : 1, *v*/*v*), H_2_SO_4_ (0.25 m), Ni foam cathode (1 cm^2^). [a] H_2_SO_4_ (0.13 m). Yields were determined by ^1^H NMR spectroscopy using an internal standard (isolated yields in parentheses). Deuterium incorporations [%] were determined by ^1^H NMR spectroscopy and indicated in brackets.

The scope of the deuteration reactions was found to be similar to that of the hydrogenation reactions shown in Scheme 1. Deuterated arylacetylene derivatives **2 d**‐[D]–**2 z**‐[D] were obtained in good to high yields (53–86 %) (Scheme 3). In all instances, the deuterium content was very high, as determined by ^1^H NMR spectroscopy in combination with high‐resolution mass spectrometry (HRMS, see the Supporting Information). Remarkably, oleic acid‐9,10‐*d_2_* (**2 y**‐[D]) was synthesized in 81 % isolated yield with 95 % deuterium incorporation (for current efficiencies, see Figure S8).

**Scheme 4:**
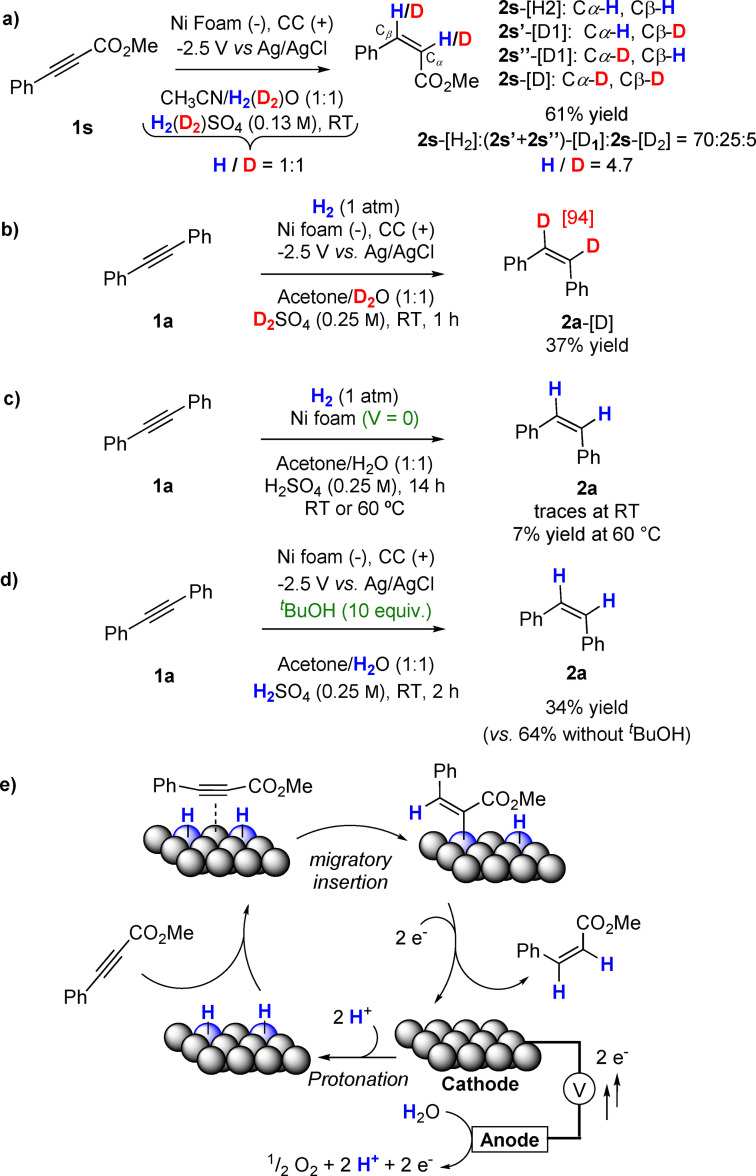
Control experiments for the electrochemical semireduction and plausible reaction mechanism.

The rates of the semideuteration reactions were significantly lower than those of the semihydrogenation reactions (i. e., **2 d** vs. **2 d**‐[D], 3 vs. 18 h). In a H/D competition experiment, olefin **2 s** was obtained with 84 % hydrogen content and only 16 % deuterium on the olefin functionality, consisting of a mixture of non‐deuterated (**2 s**‐[H_2_]), monodeuterated (**2 s′**‐[D_1_] and **2 s′′**‐[D_1_]), and fully deuterated **2 s** (**2 s**‐[D_2_]) in a ratio of 70 : 25 : 5. Importantly, the ratio H/D was identical at both olefinic carbons (Cα and Cβ in Scheme 4a), as determined by HRMS and ^1^H NMR spectroscopy (Scheme 4a and Figure S9). This indicates that proton discharge on the Ni foam is the rate limiting step (i. e., the concentration of Ni−H* species is significantly larger than that of Ni−D* species).[^40^, ^50^, ^51^, ^52^] Further, that the H/D ratio of 84 : 16 is maintained at each olefinic carbon indicates that both H atoms are transferred to the alkyne from the Ni foam, ruling out involvement of protodemetallation steps in the mechanistic cycle (Scheme 4). The formation of vinyl radicals has been also discarded, as control experiments in the presence of radical scavengers did not affect the outcome of the reaction (Figure S10). A control experiment under deuteration conditions (i. e., D_2_O/D_2_SO_4_) but with the addition of a balloon of H_2_ gave the deuterated product **2 a**‐[D] exclusively (94 %, Scheme 4b). Furthermore, when the reaction was carried out in the absence of electricity and under a hydrogen atmosphere, only 7 % of **2 a** was formed after heating at 60 °C for 14 h (Scheme 2c). These experiments suggest that the active species are Ni−H* directly produced upon protonation of the nickel atoms of the foam at the used potential, and not formed via dissociative H_2_ (or D_2_) adsorption on the metal surface. Thus, the Ni foam is an active hydrogenation catalyst only under electrochemical conditions. The results from cyclic voltammetry (CV) experiments did not provide evidence for a direct electron transfer to the alkyne (Figures S11 and S12). However, electron transfer is observed (Figures S11 and S12) due to reduction of Ni^II^ over the foam, regenerating catalytically active low‐valent Ni species on the surface. Ni foam is reported to suffer corrosion in acidic electrolytes forming H_2_ and Ni^II^ (which is reduced at potentials >−0.246 V vs. reversible hydrogen electrode, RHE).^[53]^ Furthermore, a mechanism via vinyl radicals may be ruled out as the alkene products are formed with excellent stereoselectivity (*Z*/*E* up to 20 : 1). Vinyl radicals would be formed if the mechanism involved a direct transfer of electrons to the alkyne substrates. However, vinyl radicals are not configurationally stable, which would have resulted in formation of a mixture of alkenes, where the *E*‐alkene may have been the major product for the majority of the substrates.[^54^, ^55^] That Ni−H* are the active species was further demonstrated by carrying out experiments in the presence of *tert‐*butanol (10 equiv.), which caused a dramatic decrease in the yield of **2 a**, from 64 to 34 % (Scheme 4d). This results from formation of 2‐methyl‐2‐propanol radical upon reaction of *tert‐*butanol with M−H* species.^[56]^

According to these results, a catalytic cycle for the electrochemical semihydrogenation reaction is proposed, involving Ni−H* species, where the stereospecific *syn‐*migratory insertion step explains formation of *Z‐*alkenes (Scheme 4e).

**Figure 1:**
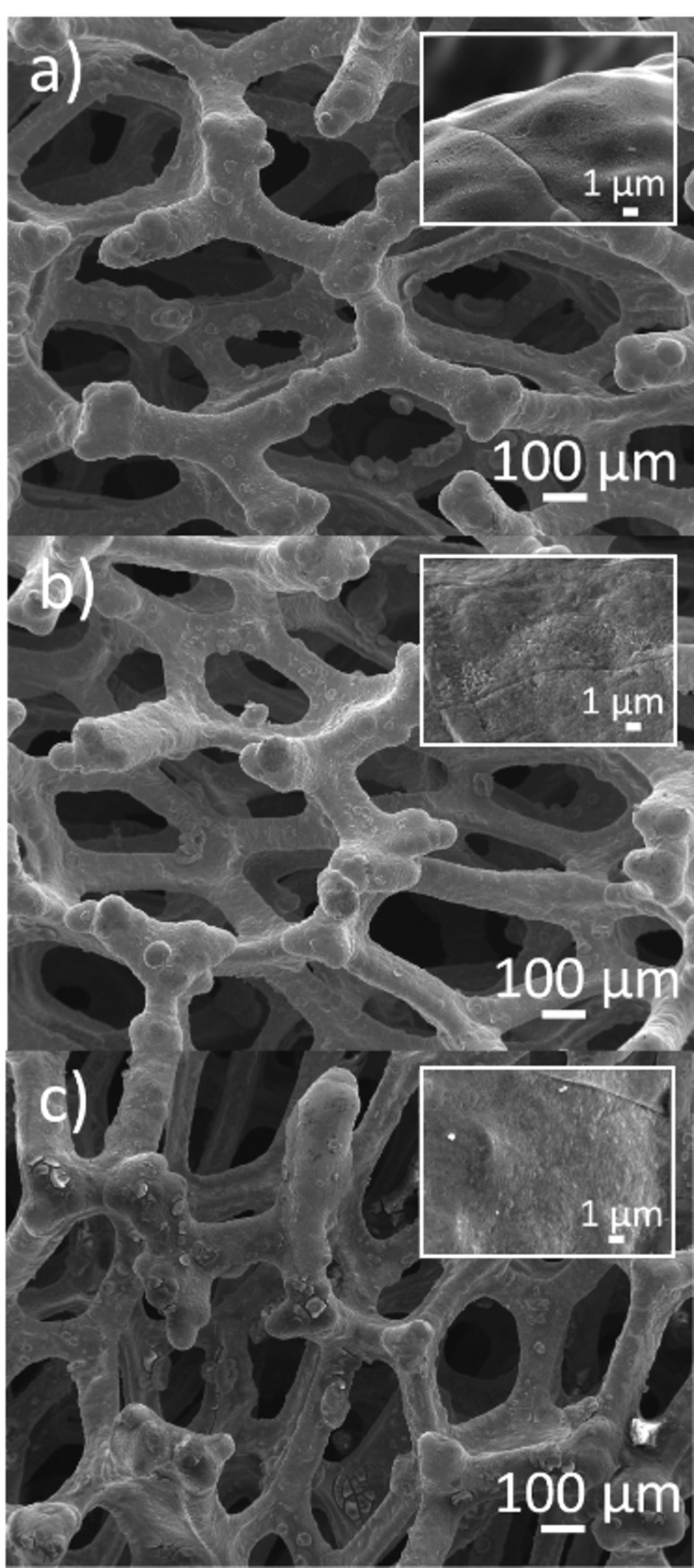
SEM images of Ni foam: (a) fresh and washed, (b) reused and washed for 11 catalytic cycles, and (c) reused for 14 catalytic cycles and unwashed for the final three cycles. Insets show the Ni foam surface at higher magnification.

The recyclability of the Ni foam as an electrocatalyst was investigated using the model substrate diphenylacetylene (**1 a**). To show that the reaction rate was similar in each recycling test, the reactions were carried out for only 2 h, shorter than the optimized 4 h. The Ni foam was washed with aq. HCl (0.5 m) and water and sonicated before each catalytic reaction for 11 cycles. It showed excellent recyclability, giving a yield of 54±9 % of *Z*‐alkene (*Z*/*E*=15 : 1) (Figure S13). After this, three more cycles were carried out without washing the foam. However, the catalyst performed equally well, suggesting that the Ni foam is a recyclable catalyst for the model substrate, regardless of washing. Scanning electron microscopy (SEM) images were collected to analyze the morphology of the Ni foam (Figure 1). The washed fresh Ni foam showed an etched surface which is typically seen after the conventional washing treatment with aq. HCl (Figure 1a).^[57]^ After recycling and washing the Ni foam for 11 catalytic cycles, the surface of the Ni foam looked similar to the fresh Ni foam (Figure 1b). This shows that the structure of Ni foam is intact, and the multiple catalytic cycles do not affect it. However, when the Ni foam was recycled for a further three runs without washing, the surface showed a deposition of some additional material, which could be sulfate salts formed during the reactions (Figure 1c). SEM images of the carbon cloth were also collected. No major changes were observed after being used for 14 cycles, which demonstrates the robust nature of this material and suggests that it could potentially be further reused (Figure S14).

## Conclusions

We have developed a straightforward method for the electrochemical semireduction of alkynes using H_3_O^+^ (D_3_O^+^) as a hydrogen (deuterium) source under very mild conditions (RT and 1 atm). Notably, we showed that a commercially available Ni foam electrode can be used and recycled up to 14 times to give a wide variety of *Z‐*alkenes with very high chemo‐ and stereoselectivity in short reaction times. Easily reducible groups such as aryl bromides and iodides were well tolerated, and dehalogenated byproducts, commonly formed under Pd catalysis, were not formed under these conditions. The method was also compatible with aliphatic groups, strained triple bonds, and other functionalities such as aldehyde‐, keto‐, and cyano‐substituted aryl groups. The highly substituted *Z*‐alkene products are important building blocks that can be used for further functionalization. We also showed that the Ni foam electrode could be used for the synthesis of deuterated analogues with high deuterium incorporations. Overall, this work demonstrates that a simple, commercially available nickel‐foam electrode can be used for electrochemical stereoselective hydrogenation and deuteration of functionalized alkynes.

## Conflict of interest

The authors declare no conflict of interest.

## Supporting information

As a service to our authors and readers, this journal provides supporting information supplied by the authors. Such materials are peer reviewed and may be re‐organized for online delivery, but are not copy‐edited or typeset. Technical support issues arising from supporting information (other than missing files) should be addressed to the authors.

Supporting Information
