# Supplementary material for: Electrochemical Proton Reduction over Nickel Foam for Z‐Stereoselective Semihydrogenation/deuteration of Functionalized Alkynes
Source: ChemSusChem. 2021 Dec 16;15(1):e202102221. doi: 10.1002/cssc.202102221 (PMC9300003; doi:10.1002/cssc.202102221)
Supplement: Supplementary file 1 — Supporting Information [file CSSC-15-0-s001.pdf]

ChemSusChem

Supporting Information

Electrochemical Proton Reduction over Nickel Foam for *Z*-Stereoselective Semihydrogenation/deuteration of Functionalized Alkynes

Alejandro Valiente, Pablo Martínez-Pardo[+], Gurpreet Kaur[+], Magnus J. Johansson, and Belén Martín-Matute*© 2021 The Authors. ChemSusChem published by Wiley-VCH GmbH. This is an open access article under the terms of the Creative Commons Attribution License, which permits use, distribution and reproduction in any medium, provided the original work is properly cited.

Contents

1. General information 1
2. Synthesis of starting materials 1
3. General procedures 1
   3.1. Semihydrogenation reactions 2
   3.2. Semideuteration reactions 2
4. Supplementary figures 3
5. Characterization of compounds 2a – 2ac / 2d-[D] – 2x-[D] 13
6. NMR spectra 22
7. References 61

1. General information

High Resolution Mass Spectrometry (HRMS): HRMS spectra were recorded on a Bruker microTOF ESI-TOF mass spectrometer measuring in electrospray ionization (ESI) in both positive and negative modes. Purifications by flash chromatography were performed using 60 Å (35−70 μm) silica gel (Acros Kieselgel 60). Nuclear Magnetic Resonance (NMR) spectroscopy: ^{1}H NMR spectra were recorded at 400 MHz and ^{13}C NMR spectra were recorded at 100 MHz on a Bruker Advance spectrometer. ^{1}H and ^{13}C NMR chemical shifts (δ) are reported in ppm from tetramethylsilane with the solvent resonance as the internal standard ($CDCl_3$: δ_H 7.26 ppm and δ_C 77.00 ppm; MeOD: δ_H 3.31 ppm and δ_C 49.00 ppm). Coupling constants (*J*) are given in Hz. Electrochemical experiments were performed using a GAMRY potentiostat (Interface 1010E). Potentials were measured versus the Ag/AgCl/KCl (sat.) reference electrode and carbon cloth (CC, 1 x 6 cm^2) was used as counter electrode. Cyclic voltammetry curves (CV) were recorded at the scan rate of 100 mV/s. Product yields were determined by isolation and by ^{1}H NMR spectroscopy using 1,2,4,5-tetrachloronitrobenzene as internal standard. Deuterium incorporations were determined by ^{1}H NMR spectroscopy and HRMS. Scanning electron microscopy (SEM): SEM images were obtained using JEOL JSM-7000F with secondary electron detector and 15.0 kV acceleration voltage. The Ni foam was mounted on the sample holder with clamps whereas carbon cloth was attached to a carbon tape. Current efficiencies were calculated according to the formula:

$$\mathrm{FE}\ (\%) = \frac{\text{mol product}}{Q/_{n\,\mathrm{x}\,\mathrm{F}}} x\ 100$$

Where Q is the total passed charge in coulombs (C), n is the number of electrons transferred and F is the Faraday constant (96485 $Cmol^{-1}$).

2. Synthesis of starting materials

Substrates **1b-1m**, **1p** were synthesized according to a reported procedure using ppm loadings of Pd.[1] Substrates **1n** and **1o** were obtained following an alternative method using a system based on $PdCl_2(PPh_3)_2$ and triethylamine as solvent.[2] Substrate **1t** was synthesized according to a reported procedure also using low loadings of $Pd(OAc)_2$ (0.01 mol%).[3] The rest of substates were obtained from the suppliers and used without further purification.

3. General procedures

General set-up and catalyst preparation: The measurements were conducted using a divided electrochemical cell which is composed of a working electrode (Ni foam), a counter electrode (carbon cloth, CC) and an Ag/AgCl reference electrode. The image of the electrolytic cell is shown below. The Ni foam and carbon cloth were used with the fixed dimensions for all the experiments *i.e.,* 1x2 cm^2 and 6x1 cm^2. Prior to the experiment, the Ni foam was washed as following; i) added 6 mL of 0.5 M aq. HCl and sonicated for 10 min, ii) replace the acid with water and decanted three times and added 6 mL of water and sonicated for 5 min, iii) exchange the water with acetone once, before using it in the reaction set-up.

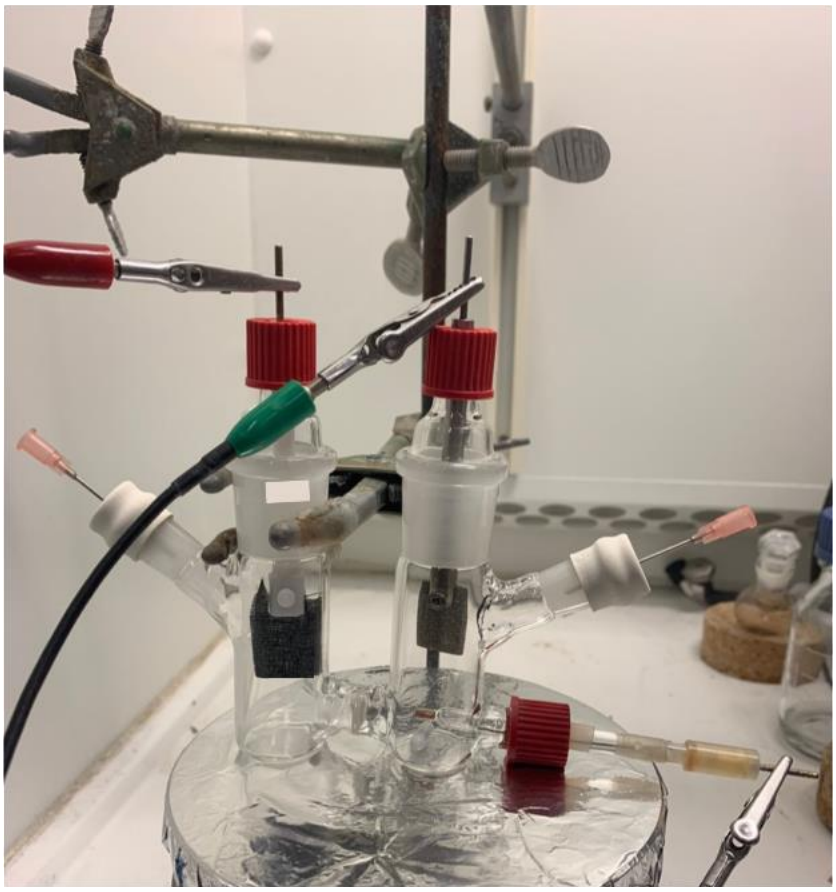

Reaction set-up for electrochemical semi-hydrogenation/deuteration of alkynes

3.1. Semihydrogenation reactions

The reaction cell was placed on a stirring plate and held in place with a clamp. Magnets were added in each chamber and reference electrode was attached. Both the chambers were charged with 12.5 mL of acetone, followed by the alkyne (0.4 mmol). Both the chambers were charged with 12.5 mL of H_2SO_4 0.25 M (Important: the alkyne should be added to the cell only when acetone is present. Direct addition into the aqueous mixture might complicate the dissolution). Carbon cloth and Ni foam were held in place and the reaction cell was closed. The potentiostat was attached to the cell and the mixture was subjected to chronoamperometry at –2.5 V under stirring. Upon reaction completion, the content from the cathodic chamber was transferred to a separating funnel and Ni foam and the chamber was rinsed twice with acetone. Water is added to the separatory funnel (c.a. 100 mL) and the product was extracted with DCM (x2) and dried over $MgSO_4$, analyzed by ^{1}H NMR spectroscopy. The reaction mixtures were purified with column chromatography. The compounds **2d**, **2e**, **2f**, **2h**, **2q**, **2u**, **2aa** and **2ac** are shown as a mixture with the alkyne or the alkane.

3.2. Semideuteration reactions

For the semideuteration experiments, a similar procedure was followed. First, the cell and the Ni foam electrode were dried under high vacuum for *c.a.* 1 h. Both chambers were charged with dry acetonitrile under argon to minimize the exposure of the system to moisture. Then, alkyne was added and solubilized prior to the addition of D_2SO_4 in D_2O solution. Upon reaction completion, the work-up was performed as indicated above and the products were purified with column chromatography.

4. Supplementary figures

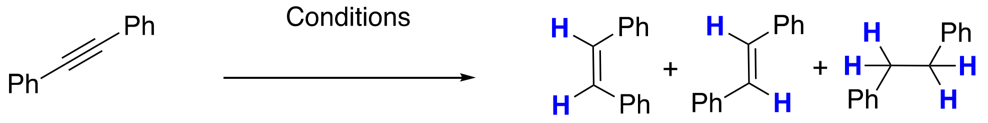

Entry	Conditions[a]	Potential (V)	Time (h)	Conv. (%)	Yield 2a / 3a / 4a (%)	*Z/E*	Current efficiency 2a / 3a / 4a (%)
1	MeOH/H_2O 1:1, H_2SO_4 (0.25 M) [b]	−1.5	2	22	18 / 2 / 2	9:1	3.6 / 0.4 / 0.8
2	DMF/H_2O 1:1, H_2SO_4 (0.25 M)	−1.5	2	35	8 / 1 / -	8:1	2.2 / 0.3 / -
3	CH_3CN/H_2O 1:1, H_2SO_4 (0.25 M)	−1.5	2	14	12 / 1 / 1	12:1	3.3 / 0.3 / 0.6
4[a]	TFE/H_2O 1:1, H_2SO_4 (0.25 M) [b]	−1.5	2	22	14 / 1 / 6	14:1	5.0 / 0.4 / 4.3
5	NMP /H_2O 1:1, H_2SO_4 (0.25 M)						
6	Acetone/H_2O 1:1, H_2SO_4 (0.13 M)	−2.5	5	97	72 / 7 / 18	10:1	5.7 / 0.6 / 3.6
7	Acetone/H_2O 1:1, H_2SO_4 (0.13 M)	−2.5	6	96	67 / 7 / 21	10:1	7.8 / 0.8 / 3.9
8	CC (–) Acetone/H_2O 1:1, H_2SO_4 (0.25 M)	−2.5	4	4	2 / 2 / -	1:1	0.3 / 0.3 / -
9	Graphite rod (+) Acetone/H_2O 1:1, H_2SO_4 (0.13 M)	−2.5	4	69	58 / 4 / 6	15:1	6.5 / 0.4 / 1.3

Reactions were performed in a divided cell using 0.4 mmol 1a in 25 mL of solvent mixture (in each chamber). Conversion, yields and *Z/E* ratios were determined by ^{1}H NMR spectroscopy using an internal standard; NMP = *N*-methylpyrrolydone. [a] Solvent ratios are indicated in *v/v*; [b] Poor solubility of **2a**

Figure S1*. Additional optimization experiments, including current efficiencies*

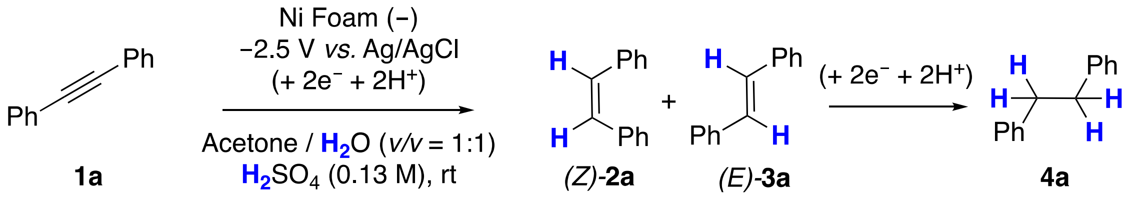

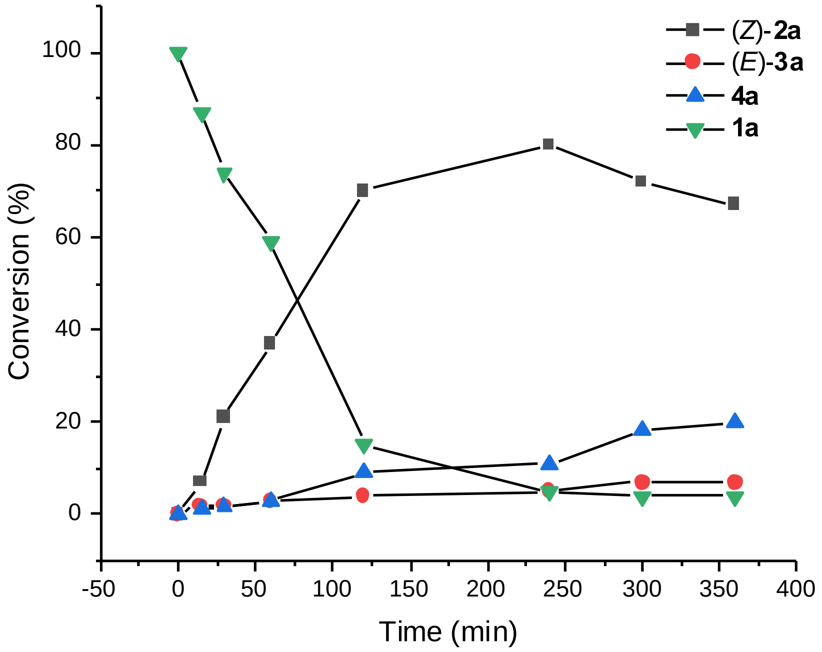

Figure S2. *Kinetic profiles for the semihydrogenation reaction under optimal conditions*

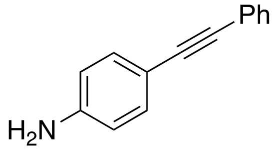

4-(phenylethynyl)aniline

^{1}H and ^{13}C values are in agreement with those reported in the literature.[4]

^{1}H NMR (400 MHz, $CDCl_3$) δ (ppm): 7.50-7.48 (2H, m, Ar), 7.36-7.28 (5H, m, Ar), 6.63 (2H, d, *J* = 8.6 Hz), 3.81 (2H, bs). ^{13}C NMR (100 MHz, $CDCl_3$) δ (ppm): 146.6 (C), 132.9 (CH), 131.3 (CH), 128. 2 (CH), 127.6 (C), 123.9 (C), 114.7 (CH), 112.7 (C), 90.1(C), 87.3 (C).

Figure S3. *Spectroscopic information of 4-(phenylethynyl)aniline obtained after the reduction of substrate **1g**.*

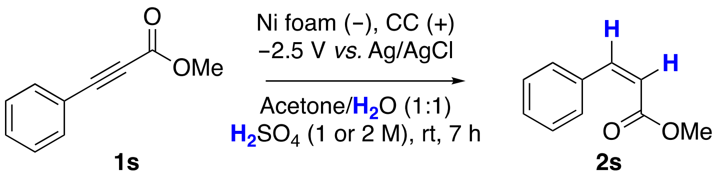

3 mmol (**1s**, 474 mg), H_2SO_4 (1 M): **2s**, 66%

6.2 mmol, (**1s,** 1 g), H_2SO_4 (2 M): **2s**, 44%

Figure S4. *Scale-up experiments of the semihydrogenation reaction*

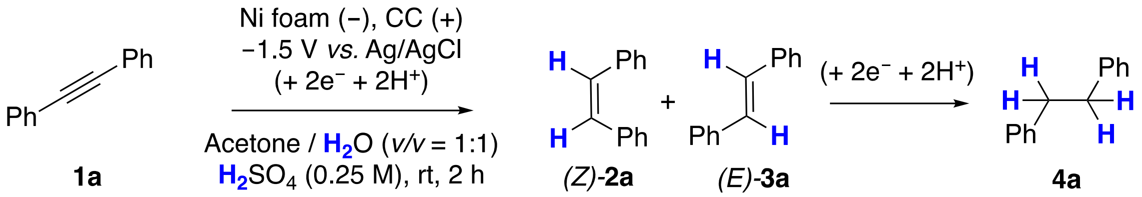

Entry	Deviation from initial conditions[a]	Yield 2a / 3a / 4a (%)	Current efficiency 2a / 3a / 4a (%)
1	none	31 / 3 / 10	9.1 / 0.9 / 5.9
2	6 h	48 / 7 / 32	5.0 / 0.7 / 6.7
3	Acetone/H_2O, 1:3	14 / 2 / 2	1.6 / 0.2 / 0.5
4	Acetone/H_2O, 3:1	8 / 1 / -	4.9 / 0.6 / -
5	H_2SO_4 (0.13 M)	29 / 2 / 2	10.5 / 0.7 / 1.4
6	H_2SO_4 (0.05 M)	8 / 2 / 1	8.2 / 2.1 / 2.1
7	NBu_4BF_4 (0.25 M)	traces	- / - / -
8	H_2SO_4 (0.13 M), –2 V	51 / 3 / 5	13.6 / 0.8 / 2.7
9	H_2SO_4 (0.13 M), –2.5 V	64 / 4 / 10	15.6 / 1.0 / 4.9
**10**	**H_2SO_4 (0.13 M), –2.5 V, 4 h**	**76 / 4 / 13**	**8.6 / 0.5/ 3.0**

Reactions were performed in a divided cell using 0.4 mmol 1a in 25 mL of solvent mixture (in each chamber). Conversion, yields and *Z/E* ratios were determined by ^{1}H NMR spectroscopy using an internal standard; [a] Solvent ratios are indicated in *v/v*.

Figure S5. *Optimization of the semihydrogenation reaction (Table 1 in main text) including current efficiencies.*

Compound	Yield (%)	Current efficiency (%)
**2b**	80	9.4
**2c**	83	7.2
**2d**	70	8.1
**2e**	51	7.3
**2f**	55	5.0
**2g**	-	-
**2h**	74	9.3
**2i**	71	9.5
**2j**	75	8.8
**2k**	73	9.3
**2l**	80	6.3
**2m**	75	6.7
**2n**	67	6.7
**2o**	71	9.0
**2p**	70	10.3
**2q**	45	3.1
**2r**	92	6.4
**2s**	73	7.3
**2t**	72	5.3
**2u**	62	18.0
**2v**	73	6.1
**2w**	91	8.1
**2x**	69	9.3
**2y**	81	5.8
**2z**	70	20.4
**2aa**	81	28.8
**2ab**	70	20.9
**2ac**	72	9.5
**2ad**	58	2.4

***Figure S6**. Current efficiencies for hydrogenated Z-alkene products*

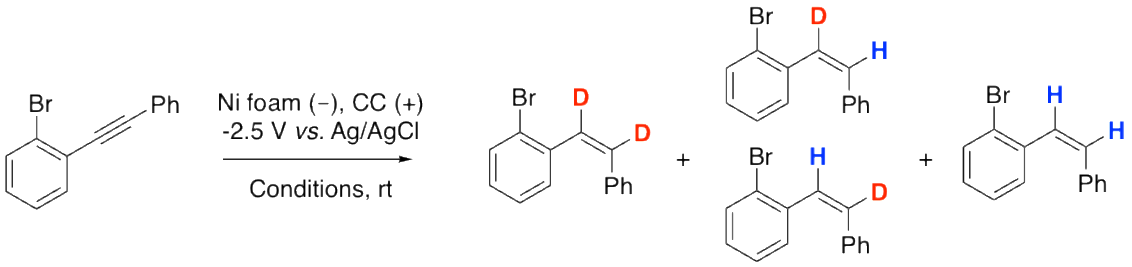

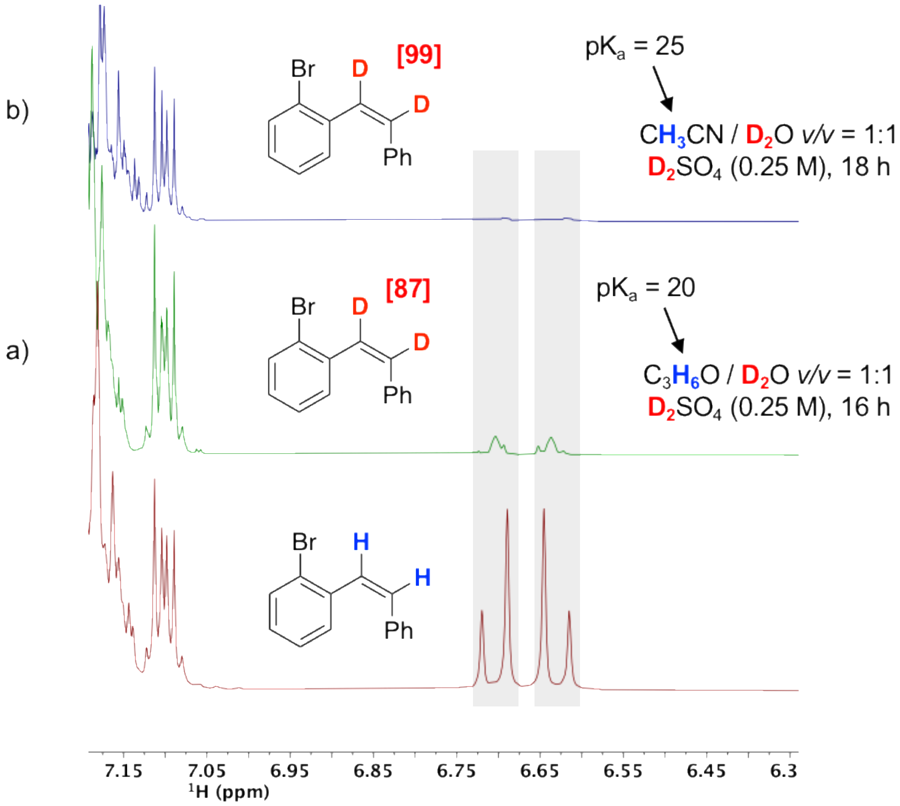

Figure S7. *Solvent effect in the deuteration reaction*

**Compound**	**Yield (%)**	**Current efficiency (%)**
**2d-[D]**	65	1.7
**2j-[D]**	84	2.0
**2l-[D]**	70	1.9
**2n-[D]**	85	2.1
**2p-[D]**	68	1.8
**2s-[D]**	86	4.7
**2t-[D]**	67	1.6
**2y-[D]**	80	6.2
**2z-[D]**	81	5.6

Figure S8. *Current efficiencies for deuterated Z-alkene products*

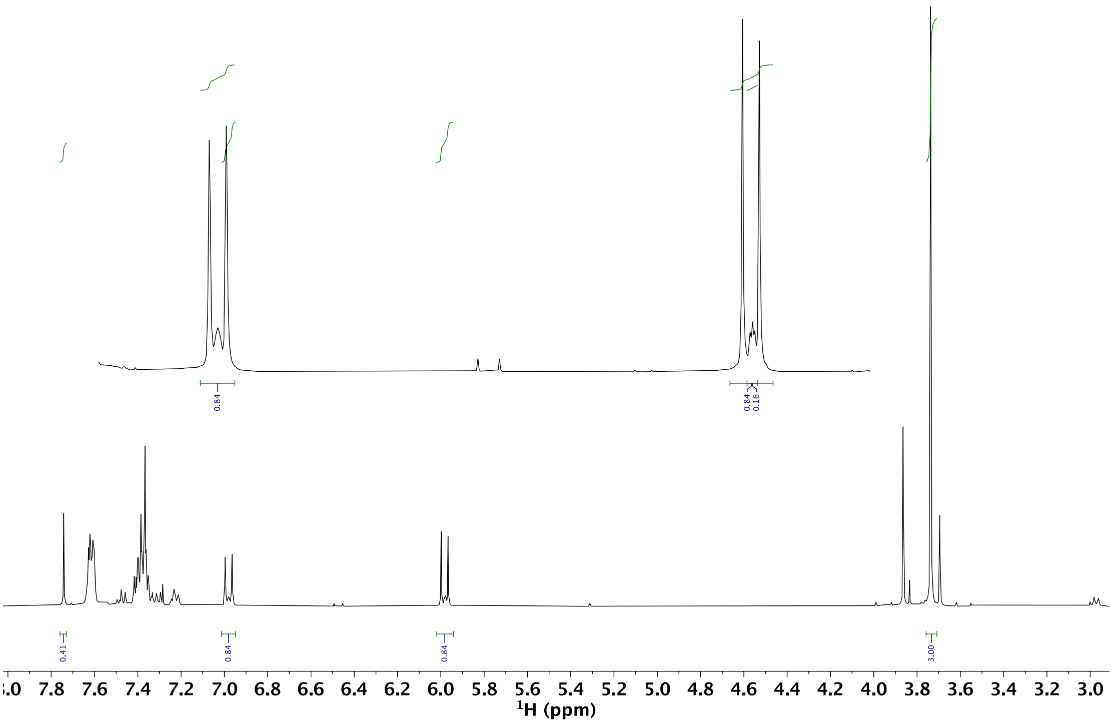

Figure S9. *Competition hydrogenation-deuteration*

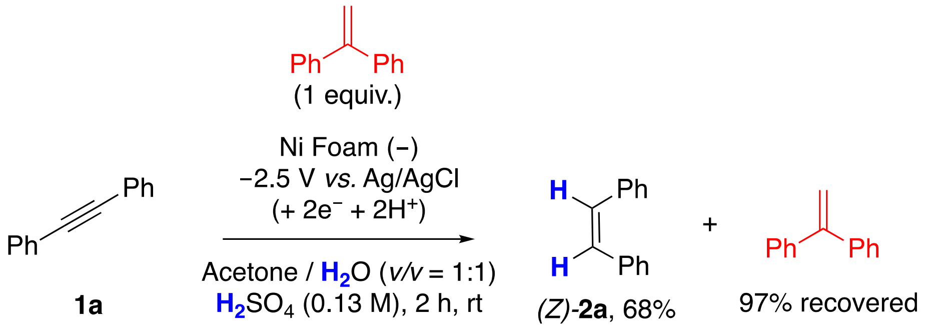

Figure S10. *Radical trapping experiment using 1,1-diphenylethylene*

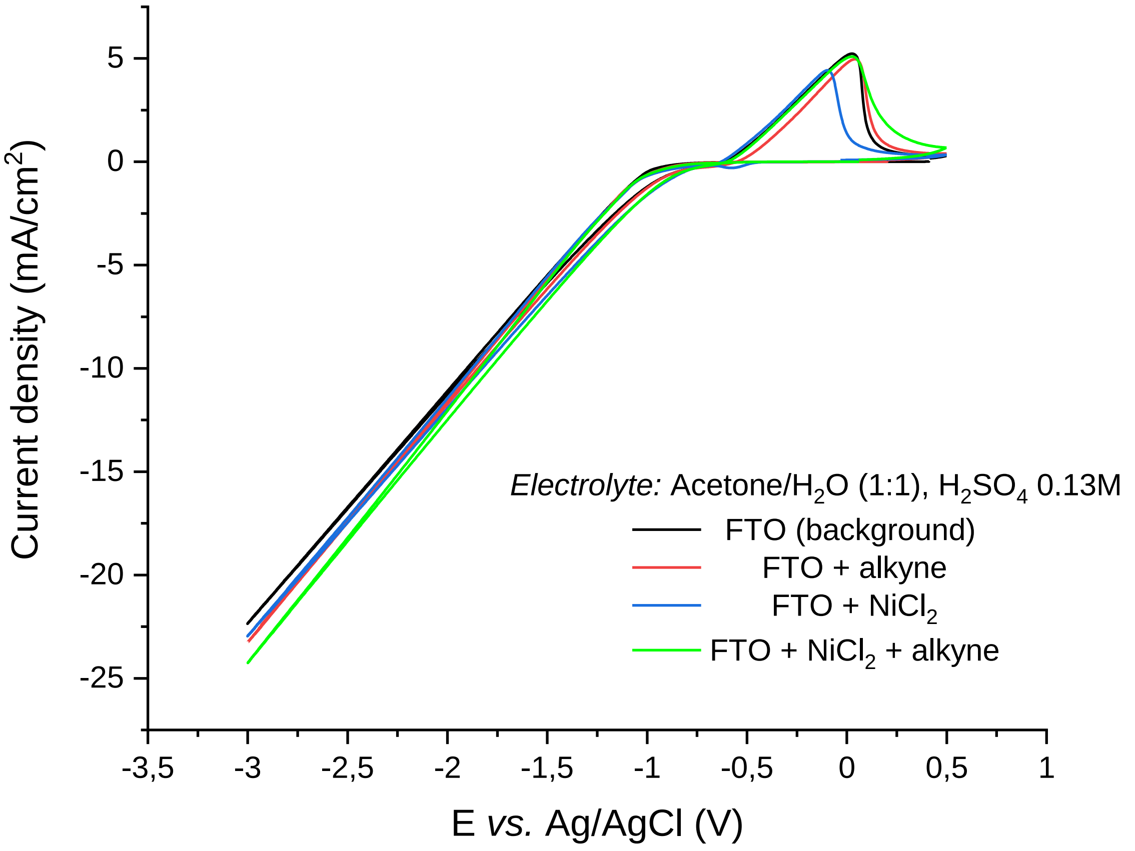

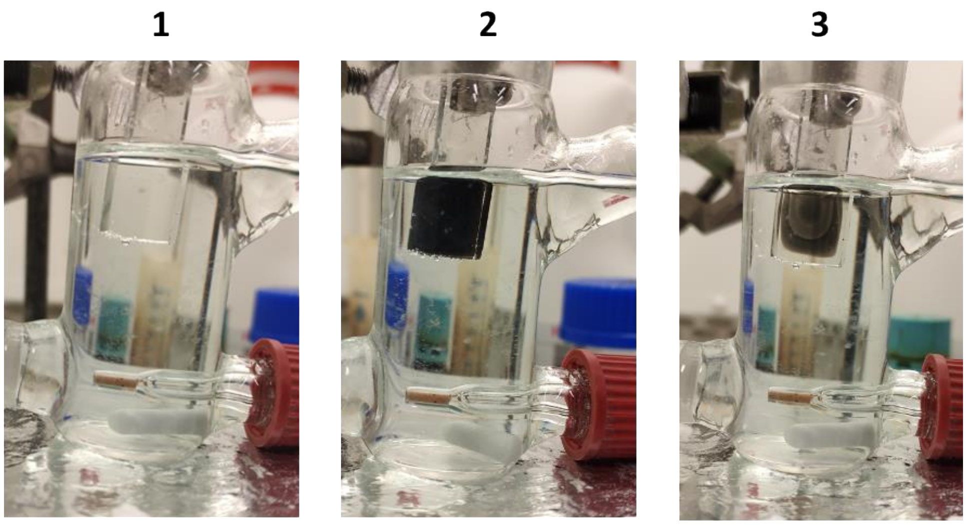

Figure S11. *Cyclic voltammetry (CV) experiments using FTO glass (picture 1) as working electrode under the optimized reaction conditions in Table 1, entry 10 and pictures of electrodeposition and corrosion of Ni on FTO (pictures 2 and 3 respectively). Conclusions: Green and blue: Electrodeposition of Ni on the FTO electrode is observed (picture 2), the substrate is not reduced. Red: No reduction of alkyne is observed. Black: background.*

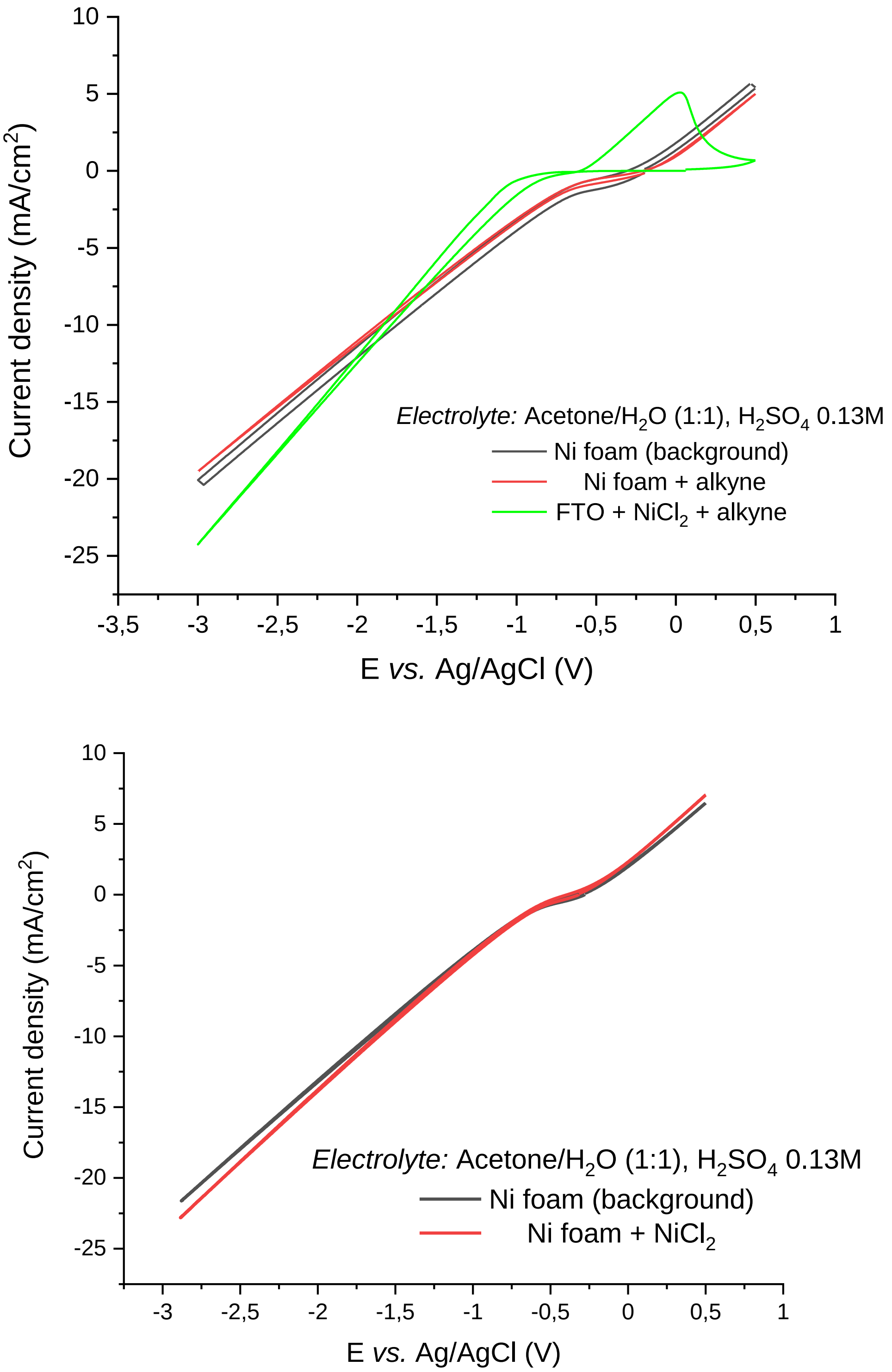

Figure S12. *Cyclic voltammetry (CV) experiments with Ni foam under the optimized reaction conditions in Table 1, entry 10. Top plot shows curves using Ni foam with and without alkyne. Curve corresponding to FTO, $NiCl_2$ and alkyne has been superimposed for comparison. In the bottom plot, curves with Ni foam with and without $NiCl_2$ are shown. At negative potentials, Ni(II) electrodeposition occurs, however both curves look identical.*

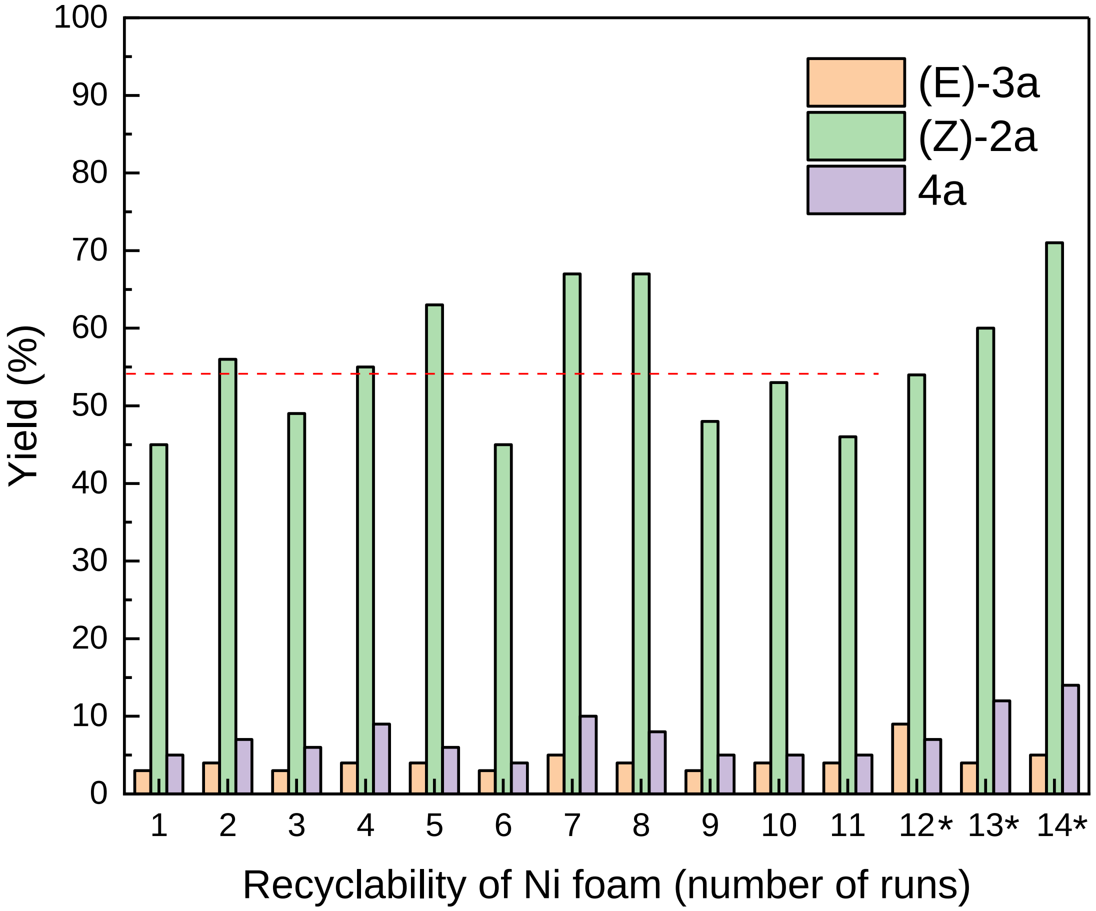

Figure S13. *Recyclability of Ni foam using model substrate diphenylacetylene. Red dotted line shows the average yield of (Z)-2a for 11 cycles. *Run 12, 13 and 14 were performed without washing the catalyst.*

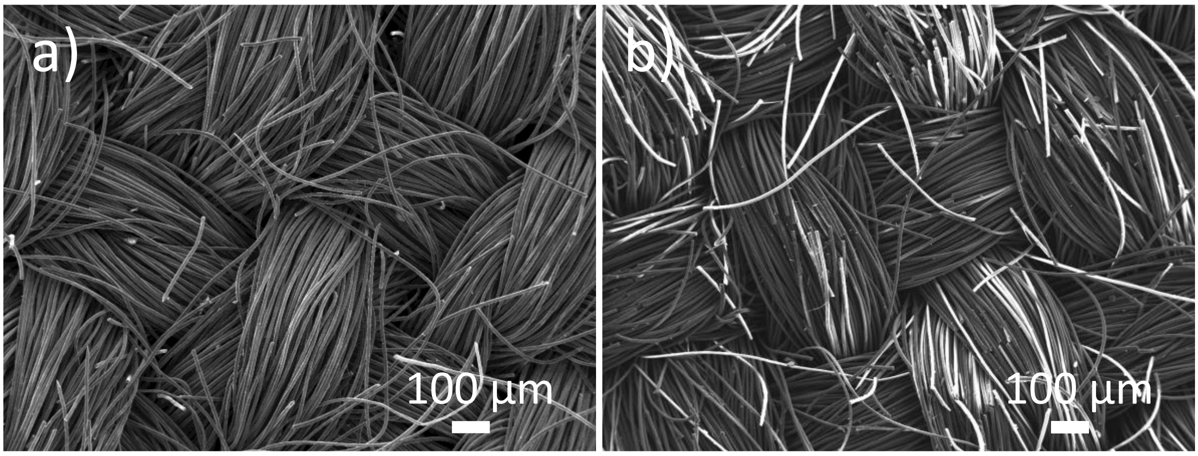

Figure S14. *SEM image of carbon cloth a) fresh and b) used for 14 times*

5. Characterization of compounds 2a – 2ac / 2d-[D] – 2x-[D]

(*Z*)-1,2-Diphenylethane (**2a**)

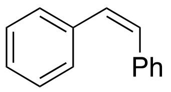

NMR yield 76% using 2,3,5,6-tetrachloronitrobenzene as internal standard. Compound **2a** was purified with column chromatography using pentane as eluent. Obtained 48.2 mg (66%) from 71.3 mg. ^{1}H and ^{13}C NMR spectra were in agreement with those reported in the literature.[5] **^{1}H NMR** (400 MHz, $CDCl_3$) δ 7.27-7.16 (10H, m, Ar), 6.60 (2H, s, CH=CH). **^{13}C NMR** (100 MHz, $CDCl_3$) δ 137.2 (C), 130.2 (CH=CH), 128.9 (CH), 128.2 (CH). 127.1 (CH).

(*Z*)-1-Methoxy-4-styrylbenzene (**2b**)

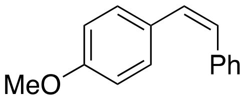

NMR yield 80% using 2,3,5,6-tetrachloronitrobenzene as internal standard. Compound **2b** was purified with column chromatography using pentane as eluent. Obtained 63.1 mg (75%) from 84.1 mg. ^{1}H and ^{13}C NMR spectra were in agreement with those reported in the literature.[5] **^{1}H NMR** (400 MHz, $CDCl_3$) δ 7.32-7.21 (7H, m, Ar), 6.79 (2H, Ar, CH), 6.56 (2H, s, CH=CH). **^{13}C NMR** (100 MHz, $CDCl_3$) δ 158.6 (C), 137.6 (C), 130.1 (CH), 129.7 (CH=CH), 128.8 (CH), 128.7 (C), 128.2 (CH), 126.9 (CH=CH), 113.5 (CH), 55.1 (OCH_3).

(*Z*)-(4-Styrylphenyl)methanol (**2c**)

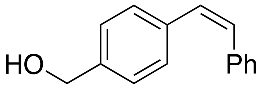

NMR yield 83% using 2,3,5,6-tetrachloronitrobenzene as internal standard. Compound **2c** was purified with column chromatography using pentane:EtOAc (9:1) as eluent. Obtained 50.8 mg (60%) from 83.3 mg. ^{1}H and ^{13}C NMR spectra were in agreement with those reported in the literature.[4] **^{1}H NMR** (400 MHz, $CDCl_3$) δ 7.31-7.19 (9H, m, Ar), 6.64 (1H, d, J = 12 Hz, CH=CH), 6.60 (1H, d, J = 12 Hz, CH=CH), 4.64 (2H, s. CH_2). **^{13}C NMR** (100 MHz, $CDCl_3$) δ 139.6 (C), 137.1 (C), 136.5 (C), 130.3 (CH=CH), 129.8 (CH=CH), 129.0 (CH), 128.8 (CH), 128.2 (CH), 127.1 (CH), 126.8 (CH), 65.0 (CH_2).

(*Z*)-1-Styryl-4-(trifluoromethoxy)benzene (**2d**)

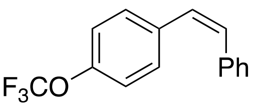

NMR yield 70% using 2,3,5,6-tetrachloronitrobenzene as internal standard. Compound **2d** was purified with column chromatography using pentane as eluent. Obtained 48.1 mg (46%) from 104.9 mg as a mixture between **2d** and **1d**. ^{1}H and ^{13}C NMR spectra were in agreement with those reported in the literature.[6] **^{1}H NMR** (400 MHz, $CDCl_3$) δ 7.26-7.19 (7H, m Ar), 7.06 (2H, d, J = 8.2 Hz, CH), 6.65 (1H, d, J = 12.2 Hz, CH), 6.55 (1H, d, J = 12.2 Hz, CH). **^{13}C NMR** (100 MHz, $CDCl_3$) δ 148.1 (C, q, J_{C-F} = 1.9 Hz), 148.0 (C), 136.8 (C), 135.8 (C), 131.1 (CH), 130.2 (CH), 128.8 (CH), 128.7 (CH), 128.3 (CH), 127.4 (CH), 120.60 (CH), 120.4 (C, q, J_{C-F} = 255 Hz). ^{19}F NMR (376 MHz, $CDCl_3$) δ –57.8 (s, OCF_3)

(*Z*)-1-Styryl-4-(trifluoromethyl)benzene (2e)

NMR yield 51% using 2,3,5,6-tetrachloronitrobenzene as internal standard. Compound **2e** was purified with column chromatography using pentane as eluent. Obtained 32.5 mg (33%) from 98.5 mg as a mixture between **2e** and **1e**. ^{1}H and ^{13}C NMR spectra were in agreement with

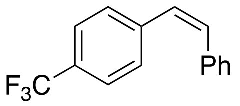

those reported in the literature.[5] **^{1}H NMR** (400 MHz, $CDCl_3$) δ 7.48 (2H, d, J = 8.2 Hz, Ar), 7.34 (2H, d, J = 8.2 Hz, Ar), 7.28 - 7.21 (5H, m, Ar), 6.73 (1H, d, J = 12.2 Hz, CH=CH), 6.60 (1H, d, J = 12.2 Hz, CH=CH). **^{13}C NMR** (100 MHz, $CDCl_3$) δ 140.9 (C, q, J_{C-F} = 1.1 Hz), 132.3 (C), 129.1 (CH), 128.82 (CH), 128.79 (CH), 128.7 (CH?), 128.44 (CH), 128.40 (CH), 127.6 CH), 125.1 (C, q, J_{C-F} = 3.8 Hz). ^{19}F NMR (376 MHz, $CDCl_3$) δ –62.5 (s, CF_3).

(*Z*)-1-(4-Styrylphenyl)ethan-1-one (2f)

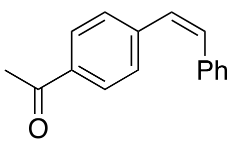

NMR yield 55% using 2,3,5,6-tetrachloronitrobenzene as internal standard. Compound **2f** was purified with column chromatography using pentane:EtOAc (95:5) as eluent. Obtained 47.7 mg (54%) from 88.1 mg as a mixture between **2f** and **4f**. ^{1}H and ^{13}C NMR spectra were in agreement with those reported in the literature.[4] **^{1}H NMR** (400 MHz, $CDCl_3$) δ 7.82 (2H, d, J = 8.1 Hz, Ar), 7.33 (2H, d, J = 8.1 Hz, Ar), 7.25-7.20 (5H, m, Ar), 6.73 (1H, d, J = 12.2 Hz, CH=CH), 6.61 (2H, d, J = 12.2 Hz, CH=CH), 2.57 (3H, s, CH_3). **^{13}C NMR** (100 MHz, $CDCl_3$) δ 197.6 (C), 142.3 (C), 136.6 (C), 135.6 (C), 129.1 (CH=CH), 129.0 (CH), 128.8 (CH), 128.7 (CH=CH), 128.33 (CH), 128.28 (CH), 127.5 (CH), 26.5 (CH_3).

(*Z*)-1-Fluoro-3-styrylbenzene (2h)

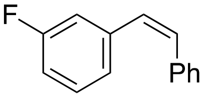

NMR yield 74% using 2,3,5,6-tetrachloronitrobenzene as internal standard. Compound **2h** was purified with column chromatography using pentane as eluent. Obtained 15.3 mg (19%) from 78.5 mg. ^{1}H and ^{13}C NMR spectra were in agreement with those reported in the literature.[7] **^{1}H NMR** (400 MHz, $CDCl_3$) δ 7.53 (1H, dd, J = 6.5, 2.8 Hz, Ar), 7.31 (1H, t, J = 4.9 Hz, Ar), 7.01 (1H, d, J = 7.6 Hz, Ar), 6.87 (1H, dt, J = 8.4, 2 Hz, Ar), 6.65 (1H, d, J = 12.2 Hz, CH=CH), 6.55 (1H, d, J = 12.2 Hz, CH=CH). **^{13}C NMR** (100 MHz, $CDCl_3$) δ 161.4 (C), 139.4 (C), 136.7 (C), 131.4 (CH), 129.6 (CH, d, J_{C-F} = 8.3 Hz), 128.9 (CH, d, J_{C-F} = 2.3 Hz), 128.8 (CH), 128.3 (CH), 127.4 (CH), 124.7 (CH), 124.7 (CH, d, J_{C-F} = 2.7 Hz), 115.5 (CH, d, J_{C-F} = 21.7 Hz), 114.0 (CH, d, J_{C-F} = 21.2 Hz). **^{19}F NMR** (376 MHz, $CDCl_3$) δ –113.6 (F, ddd, J = 10.2, 8.8, 6.1 Hz).

(*Z*)-1-Chloro-3-styrylbenzene (2i)

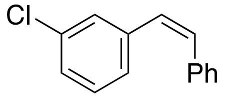

NMR yield 71% using 2,3,5,6-tetrachloronitrobenzene as internal standard. Compound **2i** was purified with column chromatography using pentane as eluent. Obtained 42.6 mg (50%) from 85.1 mg. ^{1}H and ^{13}C NMR spectra were in agreement with those reported in the literature.[8] **^{1}H NMR** (400 MHz, $CDCl_3$) δ 7.29-7.23 (6H, m, Ar), 7.18-7.12 (3H, m, Ar), 6.68 (1H, d, J = 12.2 Hz, CH=CH), 6.55 (1H, d, J = 12.2 Hz, CH=CH). **^{13}C NMR** (100 MHz, $CDCl_3$) δ 139.1 (C), 136.6 (C), 134.0 (C), 131.6 (CH), 129.4 (CH), 128.8 (CH=CH), 128.8 (CH), 128.7 (CH=CH), 128.3 (CH), 127.4 (CH), 127.1 (CH), 127.0 (CH).

(*Z*)-1-Bromo-3-styrylbenzene (2j)

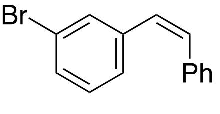

NMR yield 75% using 2,3,5,6-tetrachloronitrobenzene as internal standard. Compound **2j** was purified with column chromatography using pentane as eluent. Obtained 46.4 mg (45%) from 102.9 mg. ^{1}H and ^{13}C NMR spectra were in agreement with those reported in the literature.[9] **^{1}H NMR** (400 MHz, $CDCl_3$) δ 7.44

(1H, bs, Ar), 7.38-7.33 (1H, m, Ar), 7.27-7.25 (5H, m, Ar), 7.10 (1H, t, *J* = 7.9 Hz, Ar), 6.69 (1H, d, J = 12.2 Hz, CH=CH), 6.55 (1H, d, *J* = 12.2 Hz, CH=CH). **^{13}C NMR** (100 MHz, $CDCl_3$) δ 139.4 (C), 136.5 (C), 131.7 (CH), 131.6 (CH), 130.0 (CH), 129.7 (CH), 128.8 (CH), 128.5 (CH), 128.3 (CH), 127.44 (CH), 127.40, 122.2 (C).

(*Z*)-1-Fluoro-2-styrylbenzene (2k)

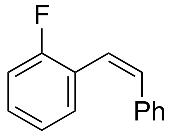

NMR yield 73% using 2,3,5,6-tetrachloronitrobenzene as internal standard. Compound **2** was purified with column chromatography using pentane as eluent. Obtained 23.7 mg (30%) from 78.5 mg. ^{1}H and ^{13}C NMR spectra were in agreement with those reported in the literature.[10] **^{1}H NMR** (400 MHz, $CDCl_3$) δ 7.28-7.20 (7H, m, Ar), 7.06 (1H, t, *J* = 9.2 Hz, Ar), 6.95 (1H, t, *J* = 7.5 Hz, Ar), 6.75 (1H, d, J = 12.2 Hz, CH=CH), 6.64 (1H, d, J = 12.2 Hz, CH=CH). **^{13}C NMR** (100 MHz, $CDCl_3$) δ 160.3 (C, d, $J_{C\text{-}F}$ = 247.7 Hz), 136.8 (C), 132.2 (CH=CH, d, $J_{C\text{-}F}$ = 1.2 Hz), 130.5 (CH, d, $J_{C\text{-}F}$ = 3.5 Hz), 128.9 (C, d, $J_{C\text{-}F}$ = 8.2 Hz), 128.7 (CH), 128.2 (CH), 127.3 (CH), 123.6 (C, d, $J_{C\text{-}F}$ = 3.5 Hz), 122.6 (CH=CH, d, $J_{C\text{-}F}$ = 3.3 Hz) 115.6 (CH, d, $J_{C\text{-}F}$ = 22.0 Hz). **^{19}F NMR** (376 MHz, $CDCl_3$) δ –114.9 (F, dt, *J* = 8.8, 6.2 Hz).

(*Z*)-1-Bromo-2-styrylbenzene (2l)

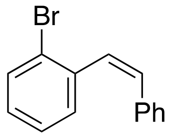

NMR yield 80% using 2,3,5,6-tetrachloronitrobenzene as internal standard. Compound **2l** was purified with column chromatography using pentane as eluent. Obtained 62.1 mg (60%) from 102.9 mg. ^{1}H and ^{13}C NMR spectra were in agreement with those reported in the literature.[10] **^{1}H NMR** (400 MHz, $CDCl_3$) δ 7.61 (1H, dd, *J* = 5.8, 3.5 Hz, Ar), 7.22-7.15 (5H, m, Ar), 7.10 (2H, dd, *J* = 5.9, 3.5 Hz, Ar), 6.70 (1H, d, *J* = 12.2 Hz, CH=CH), 6.63 (1H, d, *J* = 12.2 Hz, CH=CH). **^{13}C NMR** (100 MHz, $CDCl_3$) δ 137.9 (C), 136.3 (C), 132.7 (CH), 131.4 (CH), 130.8 (CH), 129.5 (CH), 128.9 (CH), 128.5 (CH), 128.1 (CH=CH), 127.3 (CH), 127.0 (CH=CH), 123.9 (C).

(*Z*)-1-Styrylnaphthalene (2m)

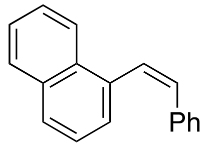

NMR yield 75% using 2,3,5,6-tetrachloronitrobenzene as internal standard. Compound **2m** was purified with column chromatography using pentane as eluent. Obtained 53.4 mg (58%) from 91.3 mg. ^{1}H and ^{13}C NMR spectra were in agreement with those reported in the literature.[7] **^{1}H NMR** (400 MHz, $CDCl_3$) δ 8.09 (1H, d, *J* = 8.7 Hz, Ar), 7.89 (1H, d, *J* = 8.9 Hz, Ar), 7.78 (1H, d, *J* = 7.4 Hz, Ar), 7.53-7.48 (2H, m, Ar), 7.38-7.33 (2H, m, Ar), 7.10 (5H, s, Ar), 7.07 (1H, d, *J* = 12.2 Hz, CH=CH), 6.85 (1H, d, *J* = 12.2 Hz, CH=CH). **^{13}C NMR** (100 MHz, $CDCl_3$) δ 136.7 (C), 135.2 (C), 133.7 (C), 132.0 (CH), 131.6 (CH), 129.0 (CH), 128.5 (CH), 128.4 (CH), 128.0 (CH), 127. 5 (CH), 127.0 (CH), 126.5 (CH), 126.00 (CH), 125.92 (CH), 125.6 (CH), 124.9 (CH).

(*Z*)-2,4-Dichloro-1-styrylbenzene (2n)

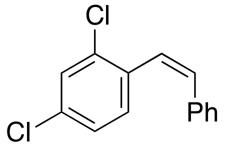

NMR yield 67% using 2,3,5,6-tetrachloronitrobenzene as internal standard. Compound **2n** was purified with column chromatography using pentane as eluent. Obtained 50.2 mg (54%) from 98.8 mg. ^{1}H and ^{13}C NMR spectra were in agreement with those reported in the literature.[11] **^{1}H NMR** (400 MHz, $CDCl_3$) δ 7.42 (1H, d, *J* = 2.1 Hz, Ar), 7.25-7.20 (3H, m, Ar), 7.17-

7.08 (3H, m, Ar), 7.01 (1H, dd, J = 8.4, 2.0 Hz, Ar), 6.74 (1H, d, J = 12.1 Hz, CH=CH), 6.60 (1H, d, J = 12.1 Hz, CH=CH). **^{13}C NMR** (100 MHz, $CDCl_3$) δ 136.1 (C), 134.5 (C), 134.4 (C), 133.4 (C), 132.4 (CH), 131.5 (CH), 129.3 (CH), 128.9 (CH), 128.3 (CH), 127.6 (CH), 126.7 (CH), 126.0 (CH).

(*Z*)-1,2,3,4,5-Pentafluoro-6-styrylbenzene (2o)

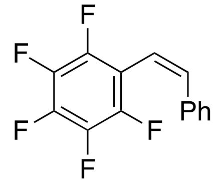

NMR yield 71% using 2,3,5,6-tetrachloronitrobenzene as internal standard. Compound **2o** was purified with column chromatography using pentane as eluent. Obtained 57.1 mg (53%) from 107.3 mg. ^{1}H and ^{13}C NMR spectra were in agreement with those reported in the literature.[12] **^{1}H NMR** (400 MHz, $CDCl_3$) δ 7.30-7.23 (3H, m, Ar), 7.14-7.12 (2H, m, Ar), 6.99 (1H, d, J = 12.0 Hz, CH=CH), 6.26 (1H, d, J = 12.0 Hz, CH=CH). **^{13}C NMR** (100 MHz, $CDCl_3$) δ 137.8 (C), 136.2 (C), 131.9 (CH=CH), 129.6 (C), 129.6 (C), 128.53 (CH), 128.51 (CH). 128.48 (CH=CH), 127.8 (CH), 112.7 (C, $J_{C\text{-}F}$ = 2.1 Hz). **^{19}F NMR** (376 MHz, $CDCl_3$) δ –155.9 (1F, t, J = 20.8 Hz), –162.20- –162.60 (2F, m), –162.2- –162.6 (2F, m)

(*Z*)-3-(2-(Thiophen-3-yl)vinyl)pyridine (2p)

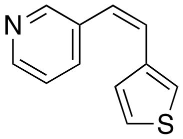

NMR yield 70% using 2,3,5,6-tetrachloronitrobenzene as internal standard. Compound **2p** was purified with column chromatography using pentane:EtOAc (7:3) as eluent. Obtained 44.1 mg (59%) from 107.3 mg. **^{1}H NMR** (400 MHz, $CDCl_3$) δ 8.53 (1H, bs, Ar), 8.46 (1H, d, J = 4.0 Hz, Ar), 7.58 (1H, d, J = 7.9 Hz, Ar), 7.25-7.14 (2H, m, Ar), 7.11 (1H, s, Ar), 6.82 (1H, d, J = 4.9 Hz, Ar), 6.67 (1H, d, J = 12.1 Hz, Ar), 6.48 (1H, d, J = 12.1 Hz, Ar). **^{13}C NMR** (100 MHz, $CDCl_3$) δ 149.9 (CH), 148.2 (CH), 137.5 (C), 135.7 (CH), 133.4 (C), 127.5 (CH), 126.7 (CH), 125.54 (CH), 125.52 (CH), 124.6 (CH), 123.0 (C). **HRMS** (ESI+): *m/z* calcd for [$C_{11}H_9NS$+H^+]: 188.0528 (found: 188.0537)

(*Z*)-4-(4-(2-Hydroxyethyl)styryl)benzonitrile (2q)

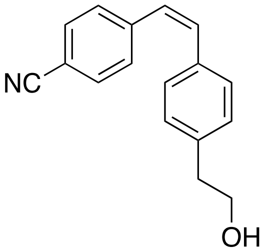

NMR yield 45% using 2,3,5,6-tetrachloronitrobenzene as internal standard. Compound **2q** was purified with column chromatography using pentane:EtOAc (9:1) as eluent. Obtained 22.8 mg (23%) from 98.9 mg as a mixture between **2q** and **1q**. **^{1}H NMR** (400 MHz, $CDCl_3$) δ 7.34 (2H, d, J = 8.2 Hz, Ar), 7.25 (2H, d, J = 8.2 Hz, Ar), 7.15-7.10 (4H, m, Ar), 6.72 (1H, d, J = 12.2 Hz), 6.54 (1H, d, J = 12.2 Hz), 3.90-3.84 (2H, m, CH_2), 2.85 (2H, t, J = 6.6 Hz, CH_2). **^{13}C NMR** (100 MHz, $CDCl_3$) δ 142.2 (C), 138.3 (C), 134.3 (C), 132.0 (CH), 131.9 (CH), 129.5 (CH), 129.1 (CH), 129.0 (CH), 128.0 (CH), 118.5 (CN), 110.4 (C), 63.3 (CH_2), 38.9 (CH_2). **HRMS** (ESI+): *m/z* calcd for [$C_{17}H_{15}NO$+Na^+]: 272.1046 (found: 272.1051)

(*Z*)-4-(3-Hydroxyprop-1-en-1-yl)benzonitrile (2r)

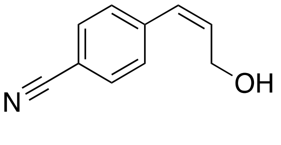

NMR yield 92% using 2,3,5,6-tetrachloronitrobenzene as internal standard. Compound **2r** was purified with column chromatography using pentane:EtOAc (9:1) as eluent. Obtained 27.4 mg (43%) from 62.9 mg. **^{1}H NMR** (400 MHz, $CDCl_3$) δ 7.61 (2H, d, J = 8.4 Hz, Ar), 7.30 (2H, d, J = 8.4 Hz, Ar), 6.55 (1H, d, J = 11.9, CH=CH), 6.02 (1H, dt, J = 11.8, 6.5 Hz,

CH=CH), 4.39 (2H, dd, J = 6.5, 1.7 Hz, CH_2). **^{13}C NMR** (100 MHz, $CDCl_3$) δ 141.1 (C), 134.2 (CH=CH), 132.0 (CH), 129.3 (CH=CH), 129.3 (CH), 118.7 (CN), 110.6 (C), 59.3 (CH_2). **HRMS** (ESI+): *m/z* calcd for [$C_{10}H_8NO–H_2O+K^+$]: 179.0132 (found: 179.0141)

Methyl (*Z*)-3-phenylacrylate (2s)

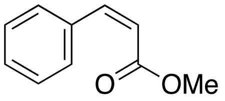

NMR yield 73% using 2,3,5,6-tetrachloronitrobenzene as internal standard. Compound **2s** was purified with column chromatography using pentane:EtOAc (9:1) as eluent. Obtained 32.9 mg (51%) from 64.1 mg. ^{1}H and ^{13}C NMR spectra were in agreement with those reported in the literature.[4] **^{1}H NMR** (400 MHz, $CDCl_3$) δ 7.60 (2H, d, J = 7.6 Hz, Ar), 7.40-7.33 (3H, m, Ar), 6.96 (1H, d, J = 12.6 Hz, CH=CH), 5.96 (1H, d, J = 12.6 Hz, CH=CH), 3.72 (3H, s, OCH_3). **^{13}C NMR** (100 MHz, $CDCl_3$) δ 166.6 (C), 143.4 (CH), 134.7 (C), 129.7 (CH), 129.0 (CH=CH), 128.0 (CH), 119.2 (CH), 51.3 (OCH_3).

Methyl (*Z*)-3-(4-iodophenyl)acrylate (2t)

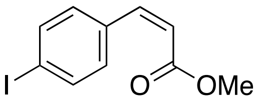

NMR yield 72% using 2,3,5,6-tetrachloronitrobenzene as internal standard. Compound **2t** was purified with column chromatography using pentane:EtOAc (9:1) as eluent. Obtained 66.9 mg (58%) from 114.4 mg. ^{1}H and ^{13}C NMR spectra were in agreement with those reported in the literature.[13] **^{1}H NMR** (400 MHz, $CDCl_3$) δ 7.69 (2H, d, J = 7.7 Hz, Ar), 7.33 (2H, d, J = 7.7 Hz, Ar), 6.85 (1H, d, J = 12.3 Hz, CH=CH), 5.97 (1H, d, J = 12.3 Hz, CH=CH). **^{13}C NMR** (100 MHz, $CDCl_3$) δ 166.2 (C), 142.3 (CH=CH), 137.2 (CH), 134.1 (C), 131.4 (CH), 120.0 (CH=CH), 95.3 (C), 51.4 (OCH_3). **HRMS** (ESI+): *m/z* calcd for [$C_{10}H_9O_2I+Na^+$]: 310.9539 (found: 310.9528).

Methyl (*Z*)-hept-2-enoate (2u)

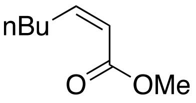

NMR yield 62% using 2,3,5,6-tetrachloronitrobenzene as internal standard. Compound **2u** was purified with column chromatography using pentane:EtOAc (9:1) as eluent. Obtained 17.2 mg (30%) from 56.1 mg as a mixture between **2u**, **1u** and **4u**. ^{1}H and ^{13}C NMR spectra were in agreement with those reported in the literature.[14] **^{1}H NMR** (400 MHz, $CDCl_3$) δ 6.23 (1H, dt, J = 11.5, 7.5 Hz, CH=CH), 5.76 (1H, d, J = 11.5 Hz, CH=CH), 3.70 (3H, s, OCH_3), 2.64 (2H, q, J = 7.4 Hz, CH_2), 2.31 (2H, dt, J = 10.5, 7.4 Hz, CH_2), 1.50-1.25 (4H, m, CH_2), 0.92-0.86 (3H, m, CH_3). **^{13}C NMR** (100 MHz, $CDCl_3$) δ 166.9 (C), 151.0 (CH=CH), 119.1 (CH=CH), 50.9 (OCH_3), 31.4 (CH_2), 29.0 (CH_2), 28.6 (CH_2), 14.0 (CH_3).

((1*R*,8*S*,9*s*,*Z*)-Bicyclo[6.1.0]non-4-en-9-yl)methanol (2v)

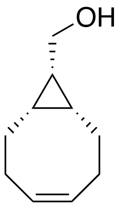

NMR yield 58% using 2,3,5,6-tetrachloronitrobenzene as internal standard. Compound **2v** was purified with column chromatography using pentane:EtOAc (8:2) as eluent. Obtained 44.8 mg (74%) from 60.0 mg. ^{1}H and ^{13}C NMR spectra were in agreement with those reported in the literature.[15] **^{1}H NMR** (400 MHz, $CDCl_3$) δ 5.65 (2H, t, J = 4.5 Hz, CH=CH), 3.73 (2H, d, J = 7.6 Hz, CH_2), 2.37 (2H, ddt, J = 15.1, 7.5, 3.7 Hz, CH_2), 2.16-2.00 (4H, m), 1.70-1.55 (2H, m), 1.42-1.37 (2H, m), 1.27-1.00 (2H, m). **^{13}C NMR** (100 MHz, $CDCl_3$) δ 129.7 (CH=CH), 60.2 (CH_2), 27.6 (CH), 23.8 (CH_2), 20.7 (CH), 19.0 (CH_2).

(*Z*)-Dec-5-en-1-ol (2w)

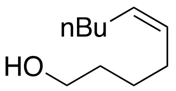

NMR yield 91% using 2,3,5,6-tetrachloronitrobenzene as internal standard. Compound **2w** was purified with column chromatography using pentane:EtOAc (8:2) as eluent. Obtained 48.9 mg (78%) from 61.7 mg. ^{1}H and ^{13}C NMR spectra were in agreement with those reported in the literature.[16] **^{1}H NMR** (400 MHz, $CDCl_3$) δ 5.40-5.30 (2H, m, CH=CH), 3.63 (2H, t, *J* = 6.5 Hz, CH_2), 2.08-2.00 (4H, m), 1.61-1.53 (2H, m), 1.44-1.37 (2H, m), 1.31-1.26 (5H, m), 0.90-0.85 (3H, m). **^{13}C NMR** (100 MHz, $CDCl_3$) δ 130.3 (CH=CH), 129.3 (CH=CH), 62.9 (CH_2), 32.3 (CH_2), 31.9 (CH_2), 26.90 (CH_2), 26.87 (CH_2), 25.8 (CH_2), 22.3 (CH_2), 14.0 (CH_3).

(*Z*)-dec-5-enal (2x)

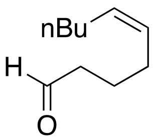

NMR yield 69% using 2,3,5,6-tetrachloronitrobenzene as internal standard. Compound **2x** was purified with column chromatography using pentane:EtOAc (95:5) as eluent. Obtained 25 mg (41%) from 60.1 mg. ^{1}H and ^{13}C NMR spectra were in agreement with those reported in the literature.[17] **^{1}H NMR** (400 MHz, $CDCl_3$) δ 9.77 (1H, bs), 5.36 (2H, m, CH=CH), 2.43 (2H, td, *J* = 7.4, 1.8 Hz), 2.08 (4H, q, *J* = 7.4 Hz), 2.00 (2H, q, *J* = 7.2 Hz), 1.69 (2H, p, *J* = 7.4 Hz), 1.45-1.22 (6H, m), 0.91-0,86 (3H, m). **^{13}C NMR** (100 MHz, $CDCl_3$) δ 202.6 (CHO), 131.3 (CH=CH), 128.2 (CH=CH), 43.3 (CH_2), 31.8 (CH_2), 26.9 (CH_2), 26.4 (CH_2), 22.3 (CH_2), 22.1 (CH_2), 14.0 (CH_3).

Oleic acid (2y)

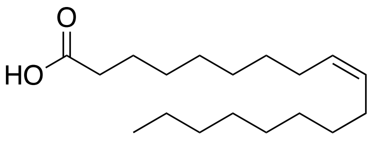

NMR yield 91% using 2,3,5,6-tetrachloronitrobenzene as internal standard. Compound **2x** was purified with column chromatography using pentane:EtOAc (8:2) as eluent. Obtained 48.5 mg (43%) from 112.2 mg. ^{1}H and ^{13}C NMR spectra were in agreement with those reported in the literature.[18] **^{1}H NMR** (400 MHz, $CDCl_3$) δ 5.39-5.30 (2H, m, CH=CH), 2.36-2.33 (2H, m), 2.02-2.00 (5H, m), 1.67-1.60 (3H, m), 1.33-1.26 (19H, m), 0.90-0.86 (3H, m). **^{13}C NMR** (100 MHz, $CDCl_3$) δ 179.9 (C), 130.0 (CH=CH), 129.7 (CH=CH), 34.0 (CH_2), 31.9 (CH_2), 29.8 (CH_2), 29.7 (CH_2), 29.5 (CH_2), 29.3 (CH_2), 29.1 (CH_2), 29.06 (CH_2), 29.03 (CH_2), 27.21 (CH_2), 27.15 (CH_2), 24.6 (CH_2), 22.7 (CH_2), 14.1 (CH_3).

Hex-5-enoic acid (2z)

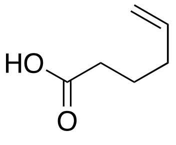

NMR yield 70% using 2,3,5,6-tetrachloronitrobenzene as internal standard. Compound **2y** was purified with column chromatography using pentane:EtOAc (8:2) as eluent. Obtained 11.7 mg (26%) from 44.9 mg. ^{1}H and ^{13}C NMR spectra were in agreement with those reported in the literature.[19] **^{1}H NMR** (400 MHz, $CDCl_3$) δ 5.78 (1H, ddt, *J* = 16.9, 10.2, 6.7 Hz, CH=CH), 5.07-4.98 (2H, m, CH=CH), 2.36 (2H, q, *J* = 8.0 Hz), 2.12 (2H, q, *J* = 7.0 Hz), 1.74 (2H, p, *J* = 7.5 Hz). **^{13}C NMR** (100 MHz, $CDCl_3$) δ 179.8 (C), 137.5 (CH=CH), 115.5 (CH=CH), 32.2 (CH_2), 32.9 (CH_2), 23.7 (CH_2).

1-Methoxy-4-vinylbenzene (2aa)

NMR yield 81% using 2,3,5,6-tetrachloronitrobenzene as internal standard. Compound **2z** was purified with column chromatography using pentane:EtOAc (95:5) as eluent. Obtained 31.8

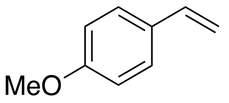

mg (59%) from 52.9 mg. ^{1}H and ^{13}C NMR spectra were in agreement with those reported in the literature.[20] **^{1}H NMR** (400 MHz, $CDCl_3$) δ 7.35 (2H, d, *J* = 8.4 Hz, Ar), 6.87 (2H, d, *J* = 8.4 Hz, Ar), 6.67 (1H, dd, *J* = 17.6, 10.9 Hz, CH=CH), 5.61 (1H, d, *J* = 17.6 Hz, CH=CH), 5.13 (1H, d, *J* = 10.9 Hz, CH=CH), 3.82 (3H, s, OCH_3). **^{13}C NMR** (100 MHz, $CDCl_3$) δ 159.3 (C), 136.2 (CH=CH), 130.4 (C), 127.4 (CH), 113.9 (CH), 111.6 (CH=CH), 55.3 (OCH_3).

1-Bromo-4-vinylbenzene (2ab)

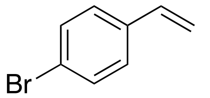

NMR yield 70% using 2,3,5,6-tetrachloronitrobenzene as internal standard. Compound **2aa** was purified with column chromatography using pentane:EtOAc (95:5) as eluent. Obtained 39.7 mg (54%) from 72.4 mg as a mixture between **2aa** and **1aa**. ^{1}H and ^{13}C NMR spectra were in agreement with those reported in the literature.[21] **^{1}H NMR** (400 MHz, $CDCl_3$) δ 7.45 (2H, d, *J* = 8.3 Hz, Ar), 7.27 (2H, d, *J* = 8.3 Hz, Ar), 6.65 (1H, dd, *J* = 17.6, 10.9 Hz, CH=CH), 5.74 (1H, d, *J* = 17.6 Hz, CH=CH), 5.28 (1H, d, *J* = 10.9 Hz, CH=CH). **^{13}C NMR** (100 MHz, $CDCl_3$) δ 136.4 (C), 135.7 (CH=CH), 133.5 (C), 131.6 (CH), 127.7 (CH), 114.6 (CH=CH).

1-Iodo-4-vinylbenzene (2ac)

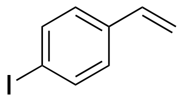

NMR yield 72% using 2,3,5,6-tetrachloronitrobenzene as internal standard. Compound **2ab** was purified with column chromatography using pentane:EtOAc (95:5) as eluent. Obtained 50.9 mg (55%) from 91.2 mg. ^{1}H and ^{13}C NMR spectra were in agreement with those reported in the literature.[22] **^{1}H NMR** (400 MHz, $CDCl_3$) δ 7.65 (2H, d, *J* = 8.4 Hz, Ar), 7.15 (2H, d, *J* = 8.4 Hz, Ar), 6.64 (1H, dd, *J* = 17.6, 10.9 Hz, CH=CH), 5.75 (1H, d, *J* = 18.3 Hz, CH=CH), 5.27 (1H, d, *J* = 11.6 Hz, CH=CH). **^{13}C NMR** (100 MHz, $CDCl_3$) δ 137.6 (CH), 137.5 (C), 135.8 (CH=CH), 128.0 (CH), 114.7 (CH=CH), 93.1 (C).

Vinyl stradiol (2ad)

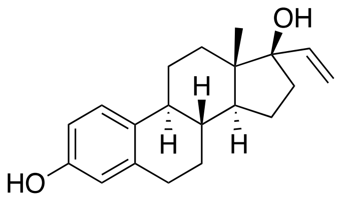

NMR yield 58% using 2,3,5,6-tetrachloronitrobenzene as internal standard. Compound **2ac** was purified with column chromatography using pentane:EtOAc (95:5) as eluent. Obtained 43.4 mg (36%) from 118.6 mg as a mixture between **2ac** and **1ac**. ^{1}H and ^{13}C NMR spectra were in agreement with those reported in the literature.[23] **^{1}H NMR** (400 MHz, $CDCl_3$) δ 7.04 (1H, d, *J* = 8.3 Hz, Ar), 6.54-6.51 (1H, m, Ar), 6.47 (1H, bs, Ar), 6.10 (1H, dd, *J* = 17.3, 10.9 Hz, CH=CH), 5.15 (1H, d, *J* = 17.3 Hz, CH=CH), 5.10 (1H, d, *J* = 10.9 Hz, CH=CH), 2.78-2.74 (2H, m), 2.35-2.15 (2H, m), 2.11-1.76 (3H, m), 1.60-1.52 (4H, m), 1.40-1.25 (6H, m), 0.93 (3H, s, CH_3). **^{13}C NMR** (100 MHz, $CDCl_3$) δ 155.8 (C), 144.6 (CH), 138.8 (CH), 132.6 (C), 127.2 (CH=CH), 116.0 (CH=CH), 113.7 (CH), 112.3 (C), 85.0 (C), 50.2, 45.15, 41.1, 41.0, 36.4, 33.4, 30.7, 28.7, 27.6, 24.2, 14.8.

(*Z*)-1-(2-Phenylvinyl-1,2-*d*2)-4-(trifluoromethoxy)benzene (2d-[D])

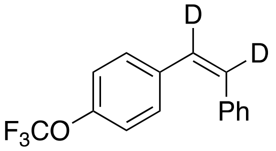

NMR yield 65% using 2,3,5,6-tetrachloronitrobenzene as internal standard. **^{1}H NMR** (400 MHz, $CDCl_3$) δ 7.27-7.19 (7H, m, Ar), 7.06 (2H, d, J = 8.0 HZ, Ar). **^{13}C NMR** (100 MHz, $CDCl_3$) δ 148.0 (C, q, J_{C-F} = 1.6 Hz), 136.2 (CH, d, J_{C-F} = 93.2 Hz), 132.4 (CH, d, J_{C-F} = 146.5 Hz), 130.2 (CH), 128.8 (CH), 128.4 (CH), 127.4 (CH), 120.6 (CH), 120.4 (C, q, J_{C-F} = 257.1 Hz). **^{19}F NMR** (376 MHz, $CDCl_3$) δ -57.8 (3F, s, CF_3). No useful peak could be found by HRMS

(*Z*)-1-Bromo-3-(2-phenylvinyl-1,2-*d*2)benzene (2j-[D])

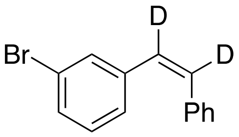

NMR yield 84% using 2,3,5,6-tetrachloronitrobenzene as internal standard. **^{1}H NMR** (400 MHz, $CDCl_3$) δ 7.41 (1H, s, Ar), 7.33 (1H, d, J = 7.9 Hz, Ar), 7.28-7.16 (5H, m, Ar), 7.17 (2H, d, J = 7.6 Hz, Ar), 7.08 (1H, t, J = 7.8 Hz, Ar). **^{13}C NMR** (100 MHz, $CDCl_3$) δ 139.3 (C), 136.4 (C), 131.7 (CH), 130.0 (CH), 129.7 (CH), 128.8 (CH), 128.4 (C), 128.3 (CH), 127.5 (CH), 127.4 (CH), 122.2 (CH). **HRMS** (ESI+): *m/z* calcd for $[C_{14}H_9BrD_2+Na^+]$: 283.0062 (found: 283.0071), D incorporation (HRMS) = 97%

(*Z*)-1-Bromo-2-(2-phenylvinyl-1,2-*d*2)benzene (2l-[D])

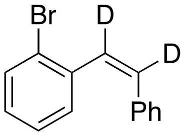

NMR yield 70% using 2,3,5,6-tetrachloronitrobenzene as internal standard. **^{1}H NMR** (400 MHz, $CDCl_3$) δ 7.62 (1H, dd, J = 5.7, 3.6 Hz, Ar), 7.22-7.16 (6H, m, Ar), 7.10 (1H, dd, J = 5.9, 3.6 Hz, Ar). **^{13}C NMR** (100 MHz, $CDCl_3$) δ 137.9 (C), 136.2 (C), 132.7 (CH), 130.8 (CH), 129.0 (CH), 128.7 (CH), 128.1 (CH), 127.3 (CH), 127.0 (CH), 129.9 (C). No useful peak could be found by HRMS

(*Z*)-2,4-Dichloro-1-(2-phenylvinyl-1,2-*d*2)benzene (2n-[D])

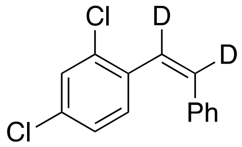

NMR yield 85% using 2,3,5,6-tetrachloronitrobenzene as internal standard. **^{1}H NMR** (400 MHz, $CDCl_3$) δ 7.42 (1H, d, J = 2.2 Hz, Ar), 7.22-7.19 (3H, m, Ar), 7.16-7.11 (3H, m, Ar), 7.01 (1H, dd, J = 8.4, 2.1 Hz, Ar). **^{13}C NMR** (100 MHz, $CDCl_3$) δ 136.0 (C), 134.4 (C), 133.4 (C), 131.5 (CH), 129.3 (CH), 128.9 (CH), 128.3 (CH), 127.6 (CH), 126.7 (CH). No useful peak could be found by HRMS

(*Z*)-3-(2-(Thiophen-3-yl)vinyl-1,2-*d*2)pyridine (2p-[D])

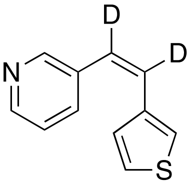

NMR yield 68% using 2,3,5,6-tetrachloronitrobenzene as internal standard. Obtained 22.1 mg (30%) from 74.1 mg. **^{1}H NMR** (400 MHz, $CDCl_3$) δ 8.53 (1H, s, Ar), 8.47 (1H, dd, J = 5.0, 1.7 Hz, Ar), 7.59 (1H, ddd, J = 7.9, 2.3, 1.7 Hz, Ar), 7.22-7.18 (1H, m, Ar), 7.17 (1H, dd, J = 5.0, 3.0 Hz, Ar), 7.12 (1H, dd, J = 3.0, 1.3 Hz, Ar), 6.83 (1H, dd, J = 5.0, 1.3Hz, Ar). **^{13}C NMR** (100 MHz, $CDCl_3$) δ 152.1 (CH), 150.0 (CH), 137.5 (C), 135.8 (CH), 133.3 (C), 127.5 (CH), 125.6 (CH), 124.6 (CH), 123.1 (CH). **HRMS** (ESI+): *m/z* calcd for $[C_{11}H_8NSD_2+H^+]$: 190.0654 (found: 190.0658), D incorporation (HRMS) = 92%

Methyl (*Z*)-3-phenylacrylate-*d*2 (2s-[D])

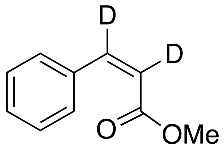

NMR yield 86% using 2,3,5,6-tetrachloronitrobenzene as internal standard. Obtained 55.8 mg (85%) from 64.1 mg. **^{1}H NMR** (400 MHz, $CDCl_3$) δ 7.63-7.60 (2H, m, Ar), 7.39-7.32 (3H, m, Ar), 3.72 (3H, s, OCH_3). **^{13}C NMR** (100 MHz, $CDCl_3$) δ 166.5 (C), 134.6 (C), 129.7 (CH), 129.1 (CH), 128.0 (CH), 51.3 (OCH_3). **HRMS** (ESI+): *m/z* calcd for [$C_{10}H_8D_2O_2$+Na^+]: 187.0699 (found: 187.0700), D incorporation (HRMS) = 96%

Methyl (*Z*)-3-(4-iodophenyl)acrylate-*d*2 (2t-[D])

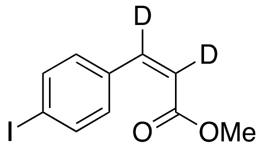

NMR yield 67% using 2,3,5,6-tetrachloronitrobenzene as internal standard. Obtained 55.9 mg (48%) from 114.4 mg. **^{1}H NMR** (400 MHz, $CDCl_3$) δ 7.69 (2H, d, *J* = 8.5 Hz, Ar), 7.34 (2H, d, *J* = 8.5 Hz, Ar), 3.71 (3H, s, OCH_3). **^{13}C NMR** (100 MHz, $CDCl_3$) δ 166.3 (C), 141.9 (CD=CD), 137.2 (CH), 134.1 (C), 131.4 (CH), 119.5 (CD=CD), 95.4 (C), 51.4 (OCH_3). **HRMS** (ESI+): *m/z* calcd for [$C_{10}H_7D_2O_2I$+Na^+]: 312.9665 (found: 312.9657), D incorporation (HRMS) = 97%

(*E*)-Hex-5-enoic-5,6-*d*2 acid (2y-[D])

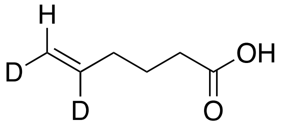

NMR yield 80% using 2,3,5,6-tetrachloronitrobenzene as internal standard. **^{1}H NMR** (400 MHz, $CDCl_3$) δ 5.01 (1H, ddd, *J* = 4.1, 2.8, 1.6 Hz, CH=CD), 2.44-2.29 (2H, m, CH_2), 2.11 (2H, t, *J* = 7.3 Hz, CH_2), 1.74 (2H, p, *J* = 7.5 Hz, CH_2). **^{13}C NMR** (100 MHz, $CDCl_3$) δ 180.2 (C), 137.0 (CD=CD), 115.1 (CD=CD), 33.3 (CH_2), 32.7 (CH_2), 26.7 (CH_2). **HRMS** (ESI–): *m/z* calcd for [$C_6H_8D_2O_2$–H^+]: 115.0734 (found: 115.0734), D incorporation (HRMS) = 93%

Oleic acid *d*2 (2x-[D])

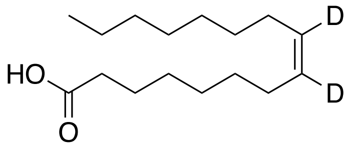

NMR yield 81% using 2,3,5,6-tetrachloronitrobenzene as internal standard. **^{1}H NMR** (400 MHz, $CDCl_3$) δ 2.34 (2H, t, *J* = 7.5 Hz, CH_2), 2.01 (2H, t, *J* = 6.6 Hz, CH_2), 1.63 (2H, p, *J* = 7.2 Hz, CH_2), 1.35-1.27 (20H, m), 0.87 (3H, t, *J* = 7.5 Hz, CH_3). **^{13}C NMR** (100 MHz, $CDCl_3$) δ 180.5 (C), 129.9-129.0 (CD=CD, m), 34.1 (CH_2), 31.9 (CH_2), 29.8 (CH_2), 29.7 (CH_2), 29.5 (CH_2), 29.3 (CH_2), 29.1 (CH_2), 29.06 (CH_2), 29.03 (CH_2), 27.07 (CH_2), 27.00 (CH_2), 24.6 (CH_2), 22.7 (CH_2), 14.1 (CH_3). **HRMS** (ESI–): *m/z* calcd for [$C_{18}H_{32}D_2O_2$–H^+]: 283.2612 (found: 283.2609), D incorporation (HRMS) = 94%

6. NMR spectra

(*Z*)-1,2-Diphenylethene (2a)

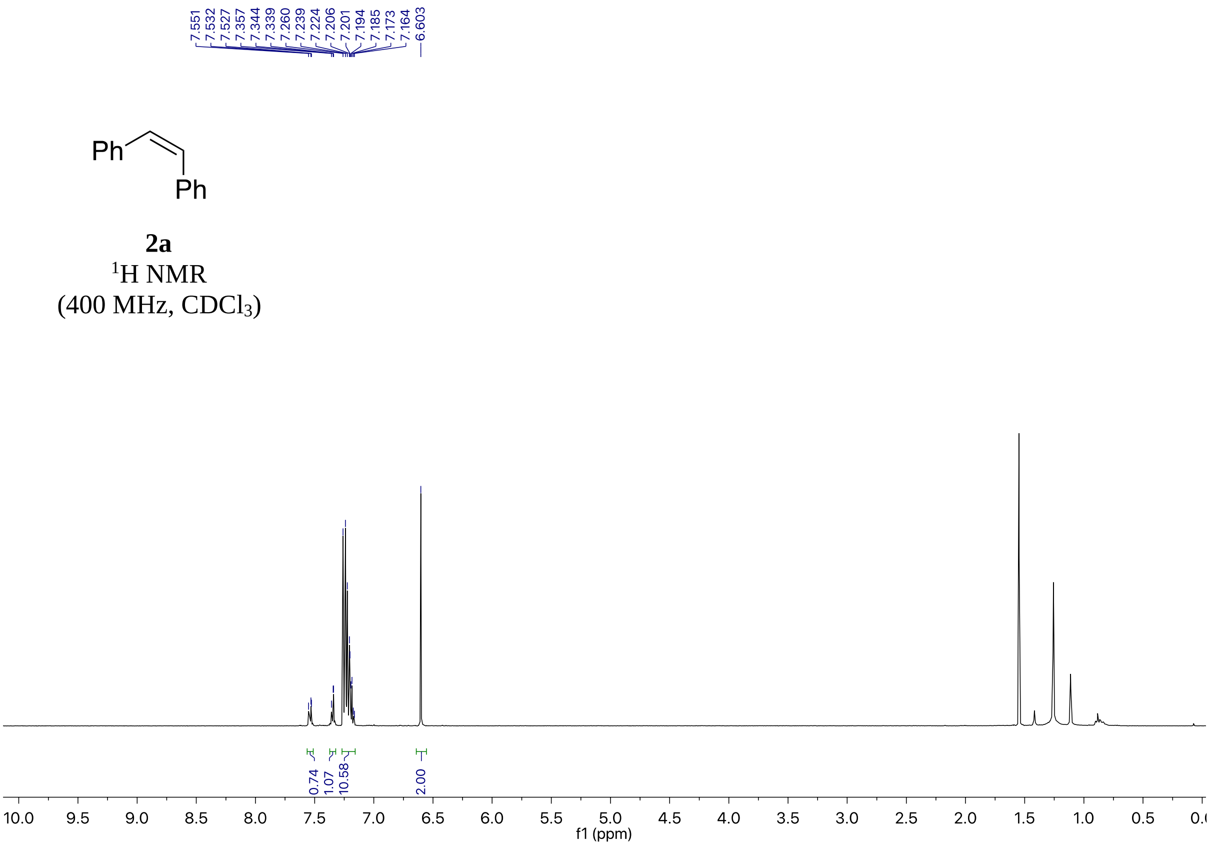

2a
^{1}H NMR
(400 MHz, $CDCl_3$)

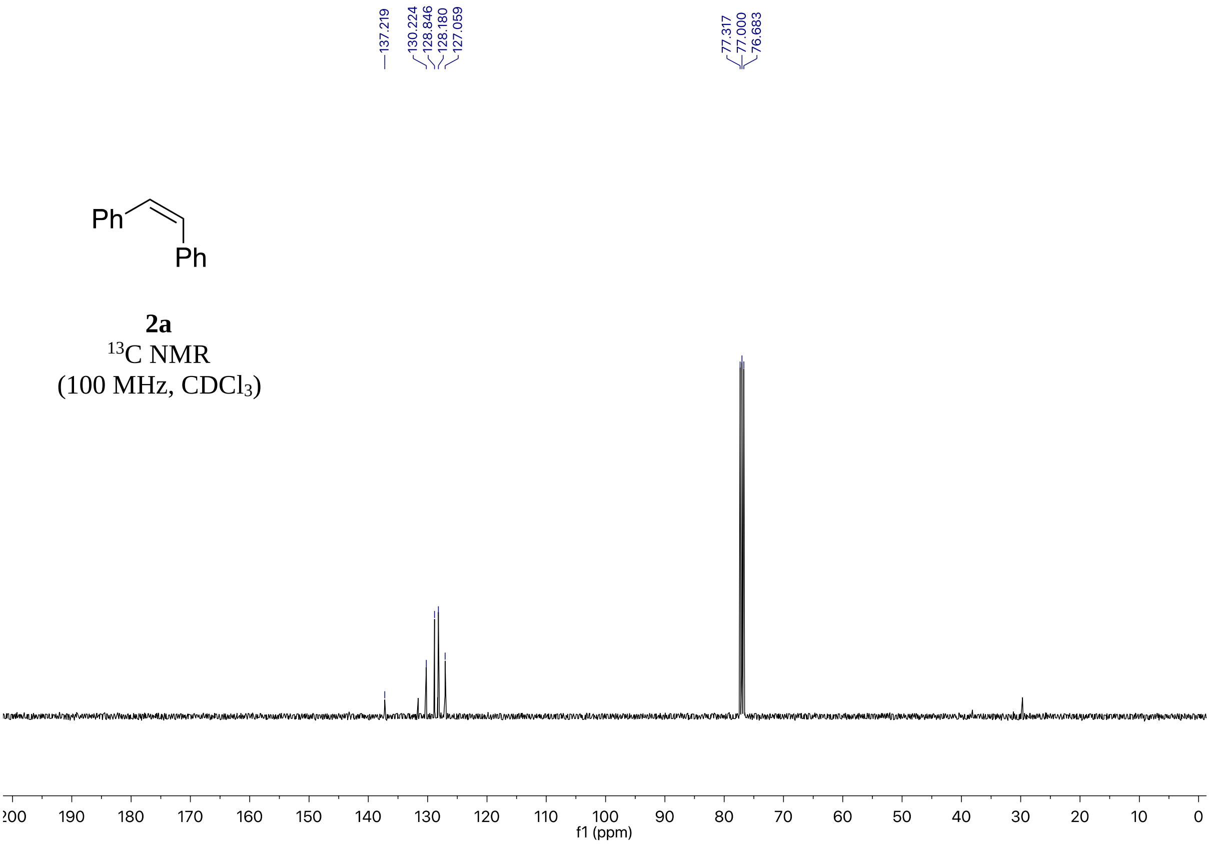

2a
^{13}C NMR
(100 MHz, $CDCl_3$)

(*Z*)-1-Methoxy-4-styrylbenzene (2b)

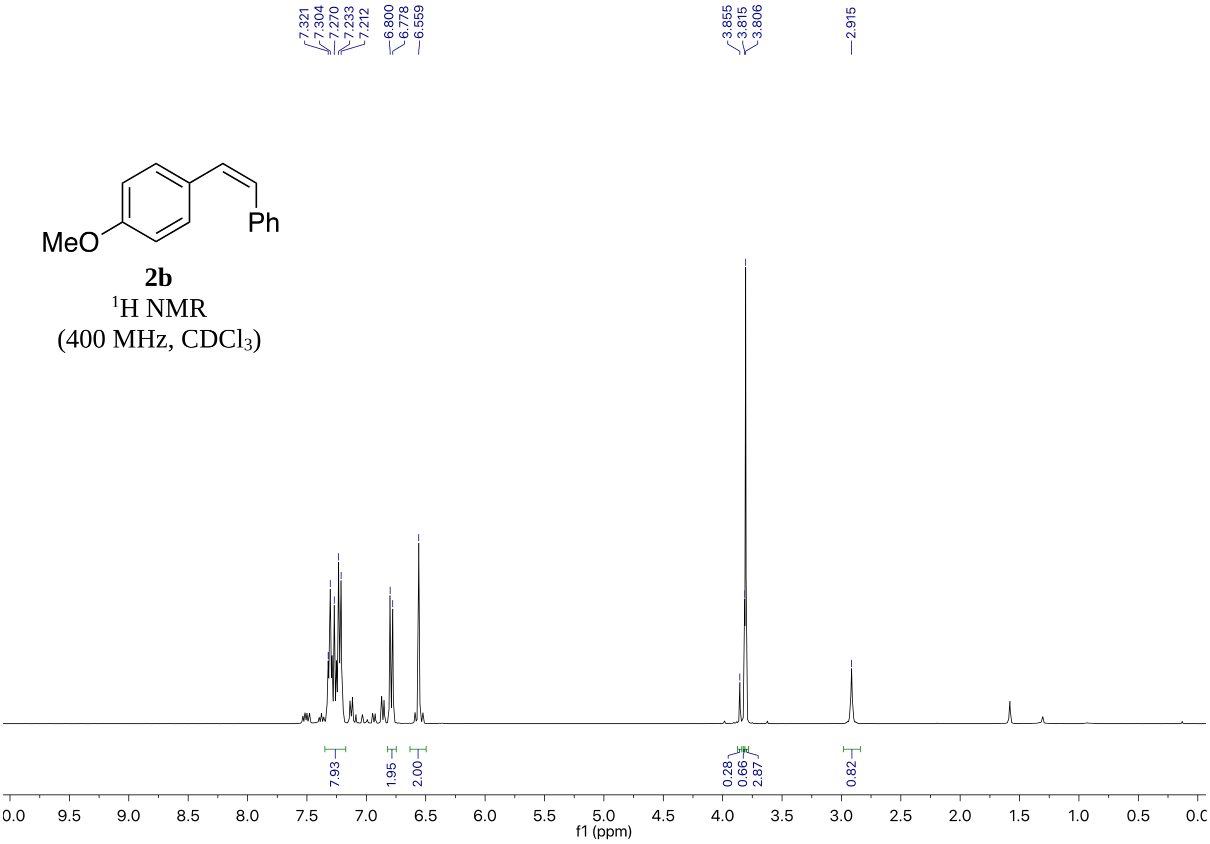

2b
^{1}H NMR
(400 MHz, $CDCl_3$)

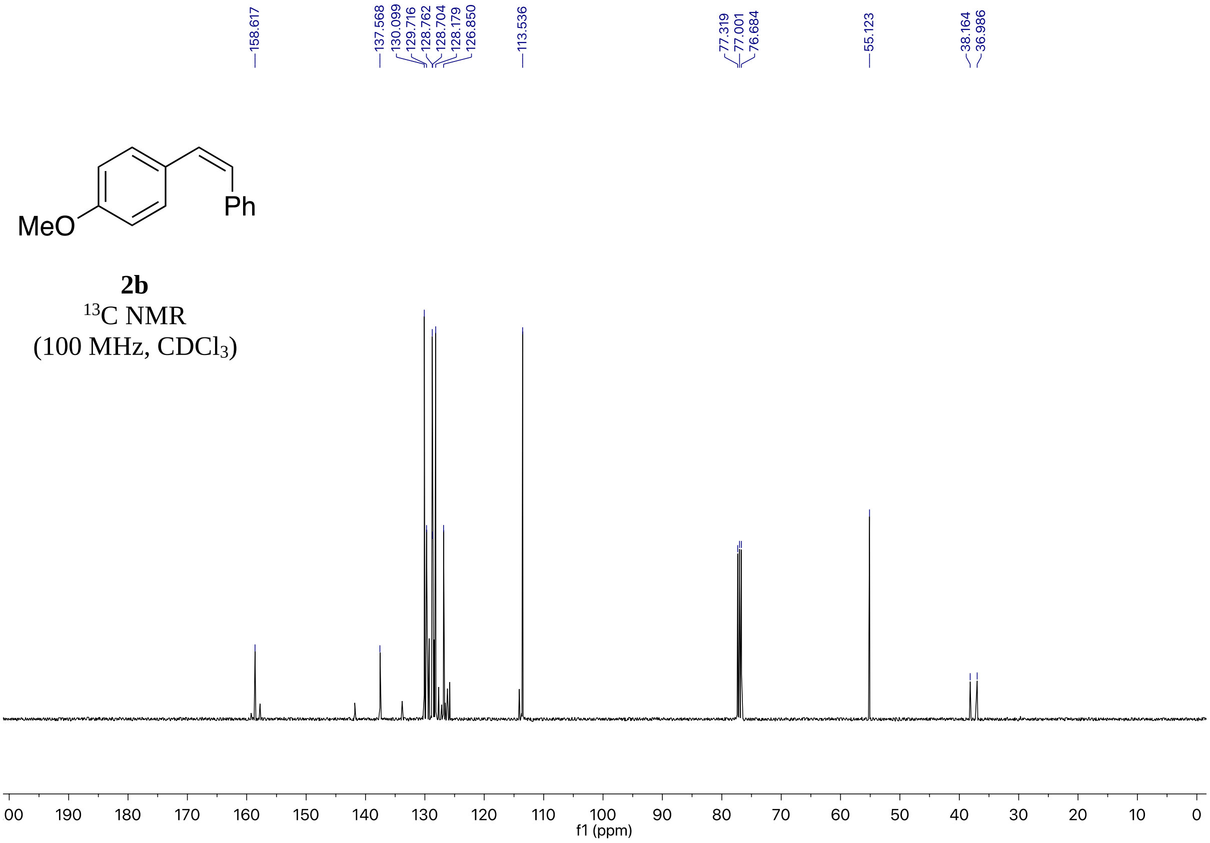

2b
^{13}C NMR
(100 MHz, $CDCl_3$)

(*Z*)-(4-Styrylphenyl)methanol (2c)

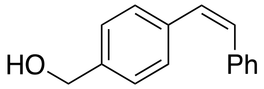

2c
^{1}H NMR
(400 MHz, $CDCl_3$)

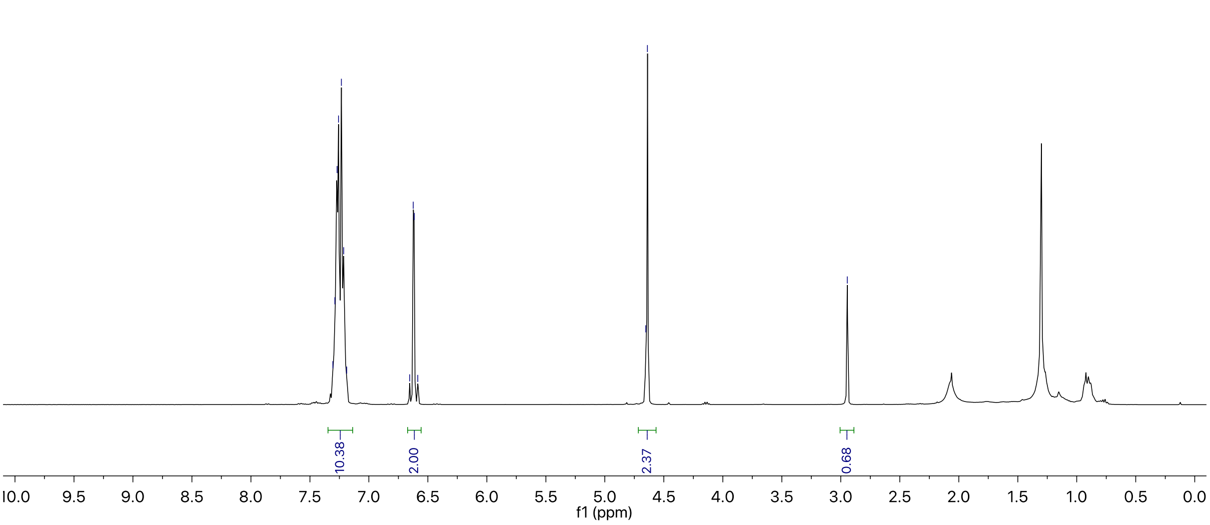

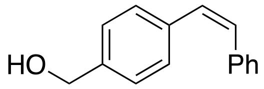

2c
^{13}C NMR
(100 MHz, $CDCl_3$)

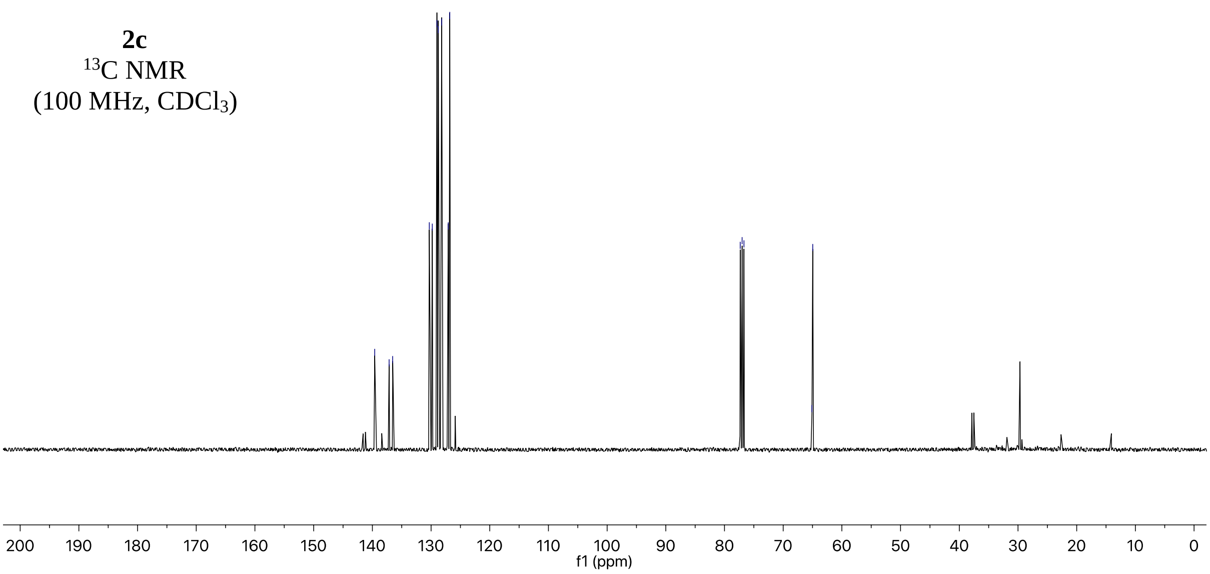

(*Z*)-1-Styryl-4-((trifluoromethoxy)methyl)benzene (2d) and (1d)

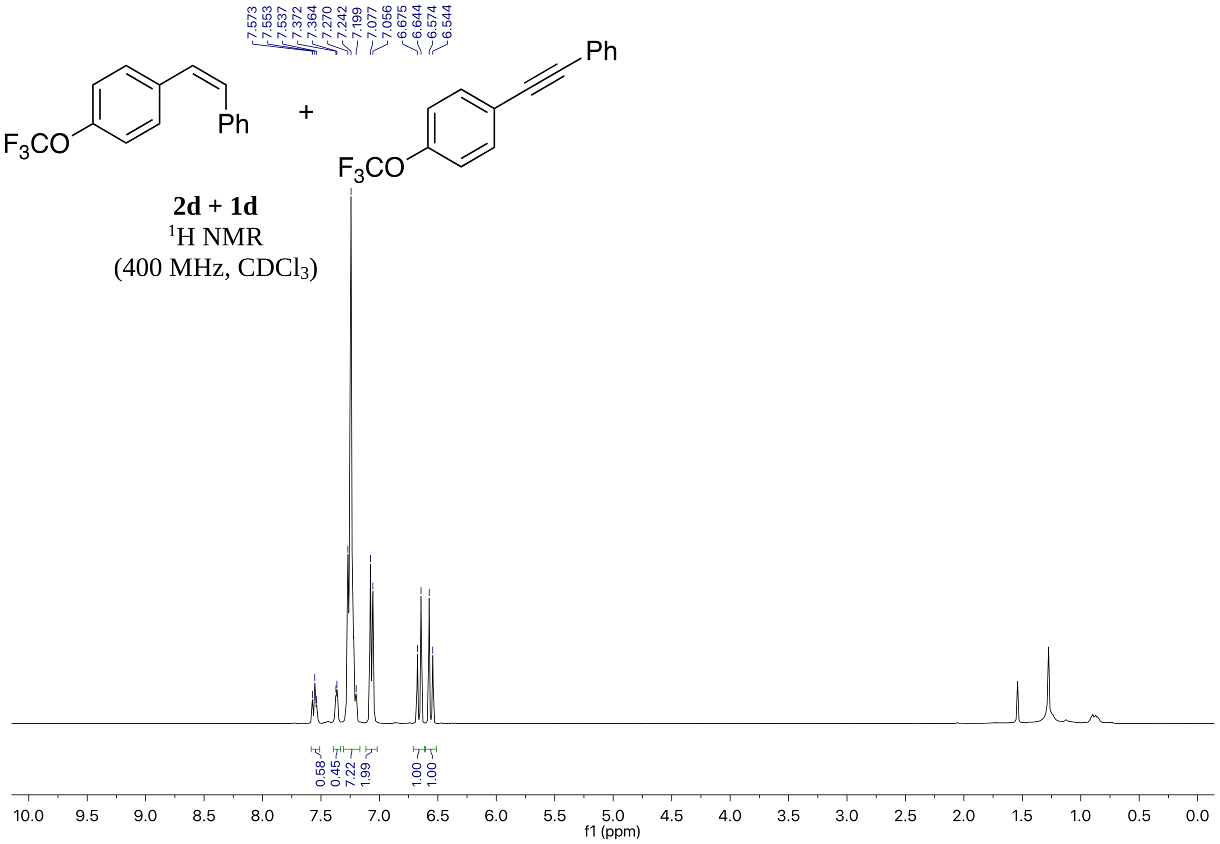

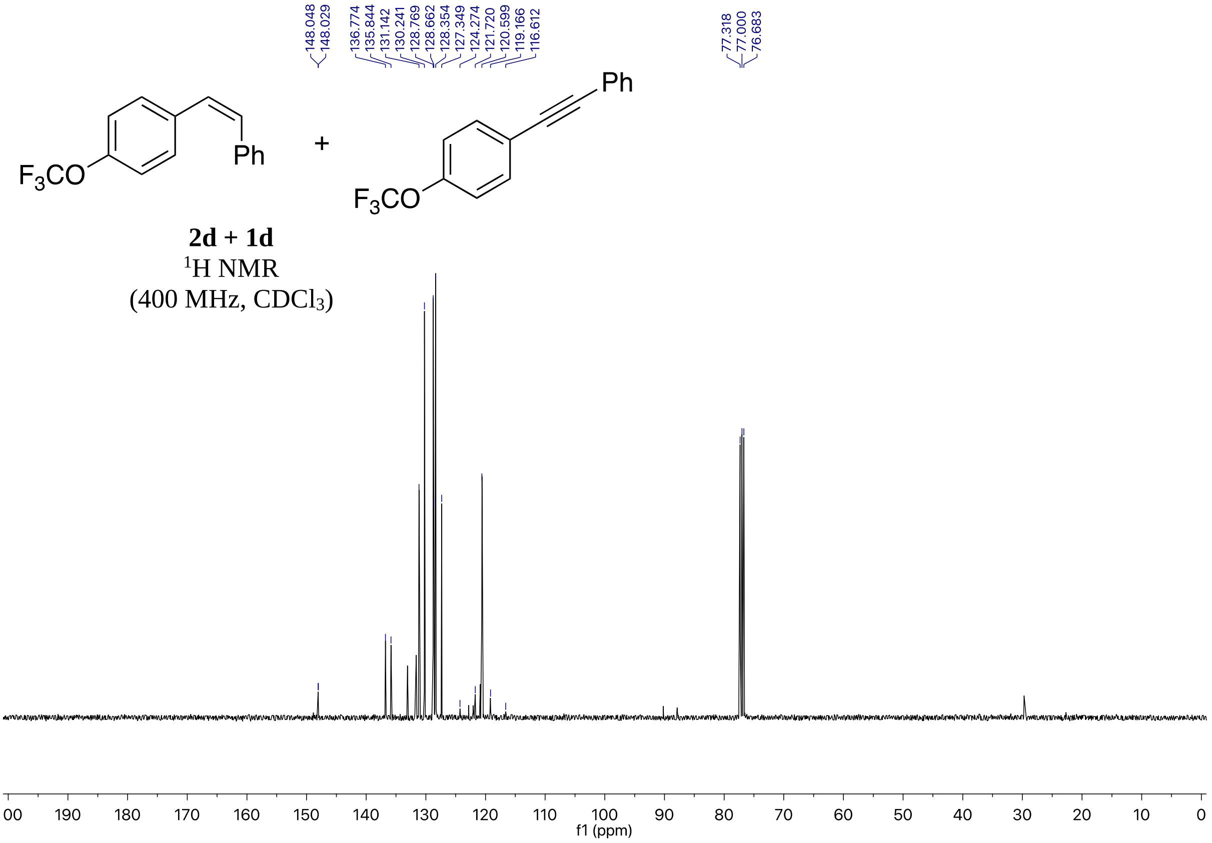

(*Z*)-1-Styryl-4-(2,2,2-trifluoroethyl)benzene (2e) and (1e)

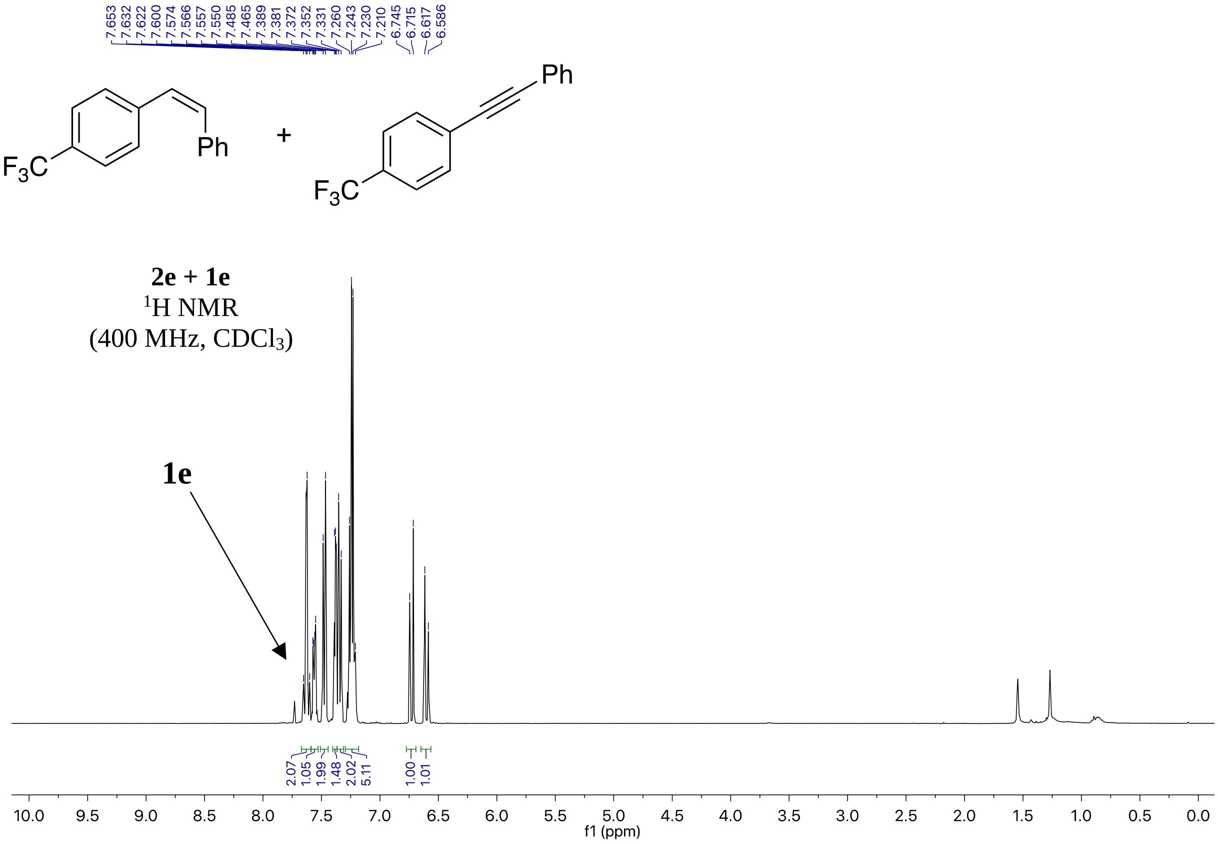

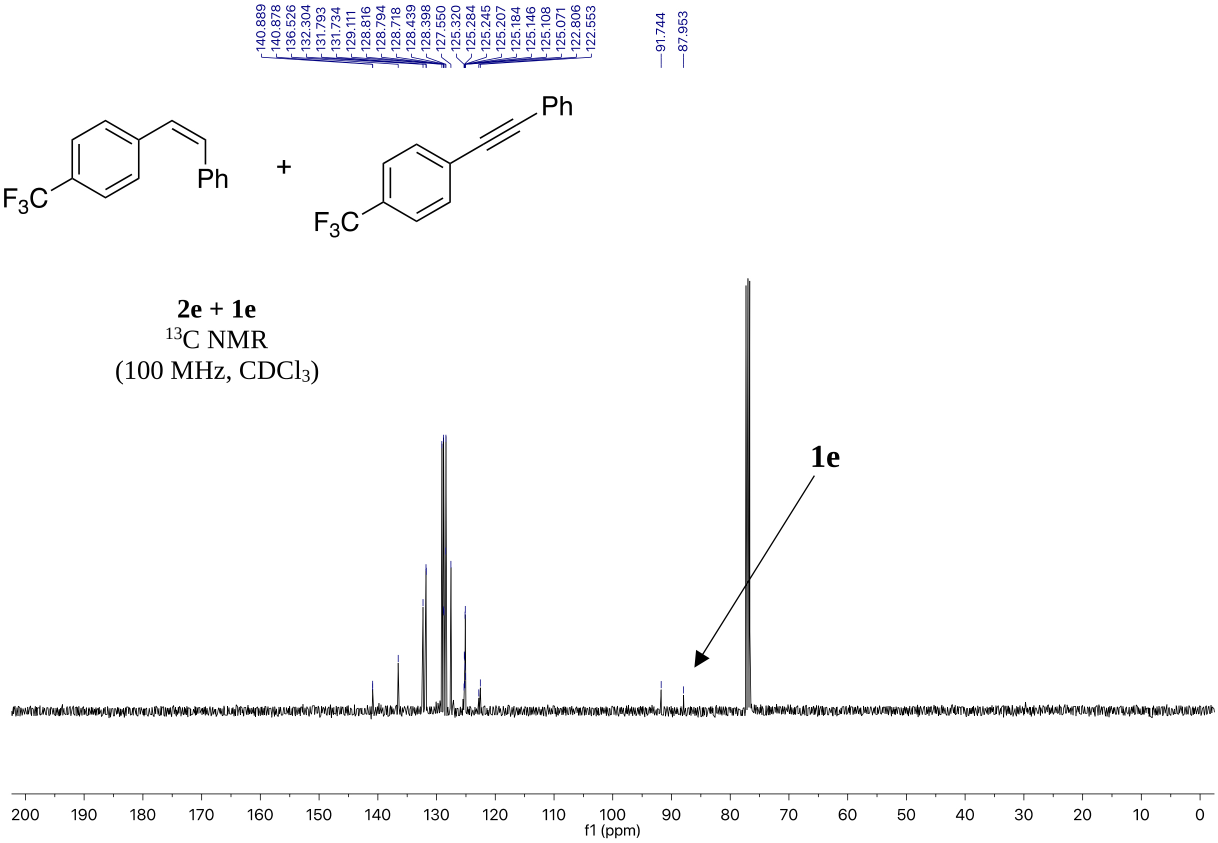

(*Z*)-1-(4-Styrylphenyl)ethan-1-one (2f) and (4f)

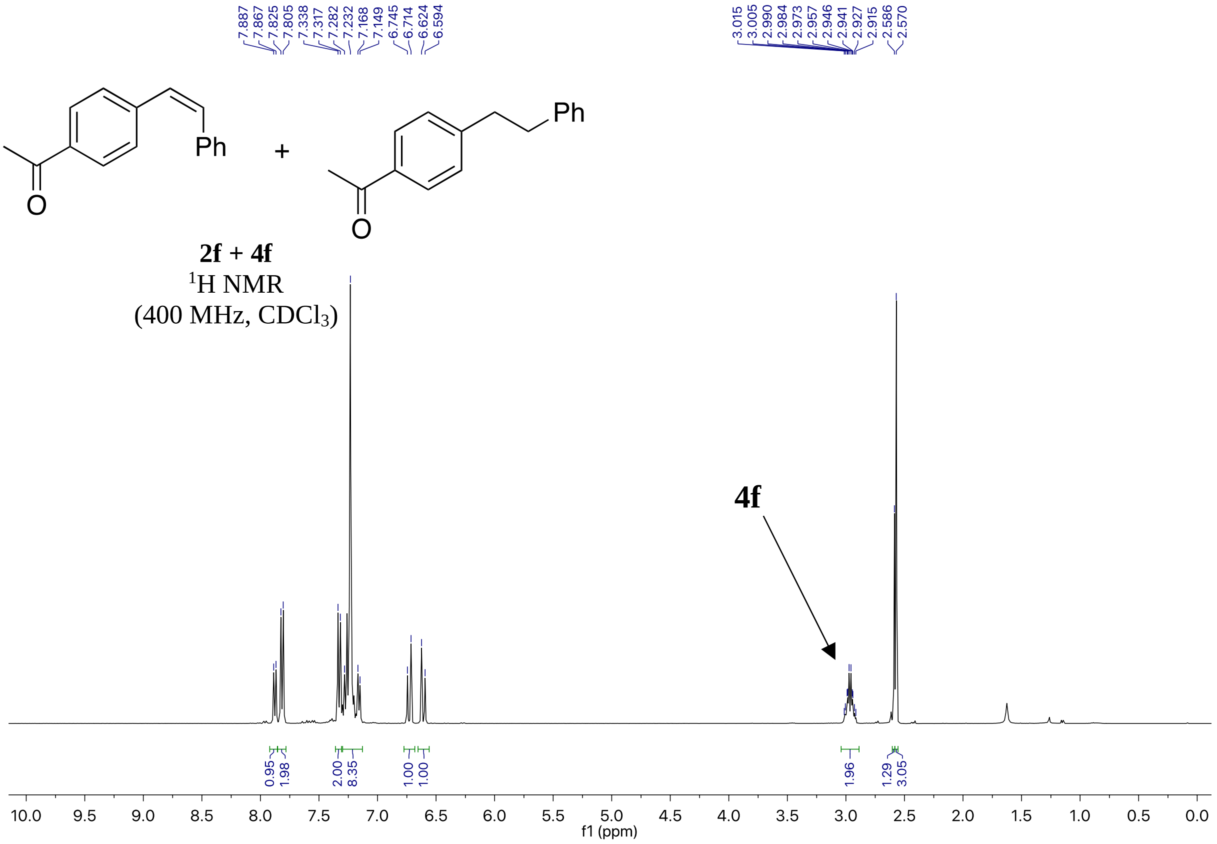

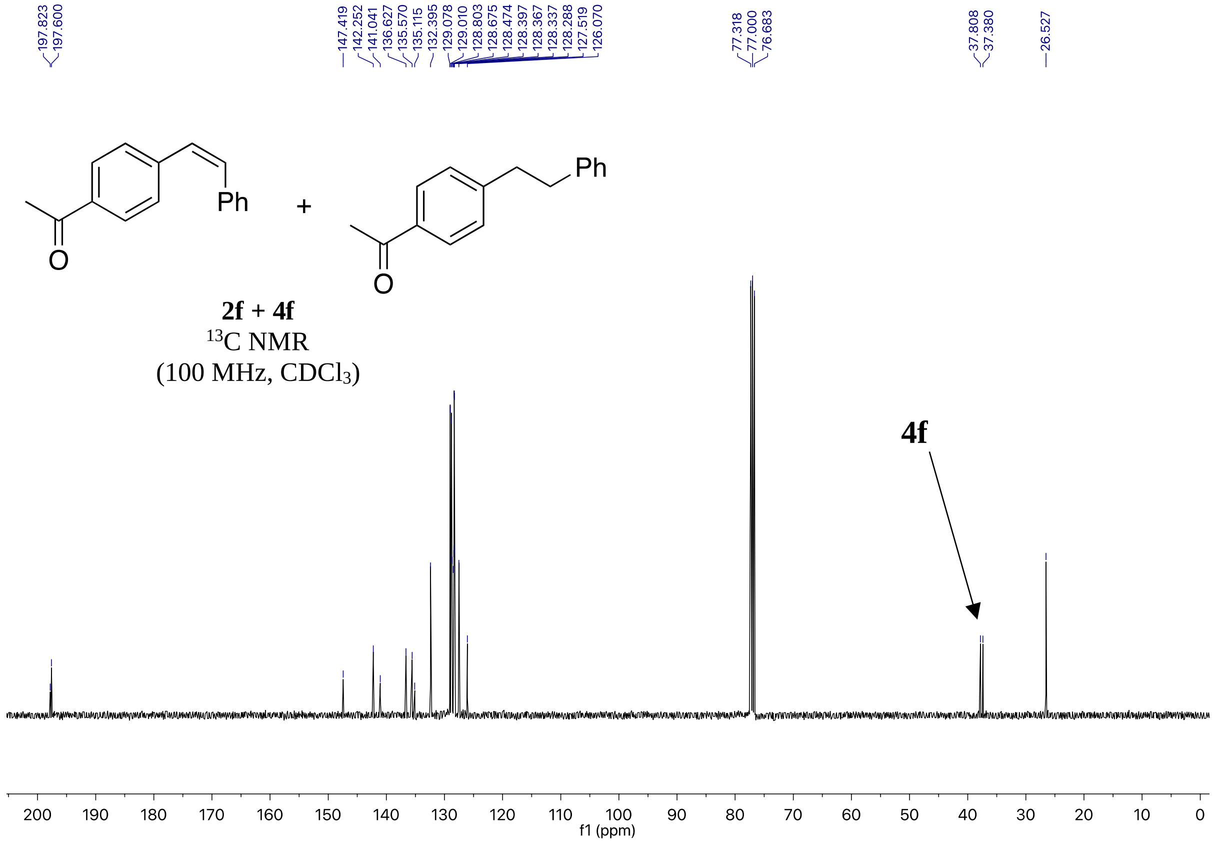

(*Z*)-1-Fluoro-3-styrylbenzene (2h) and (1h)

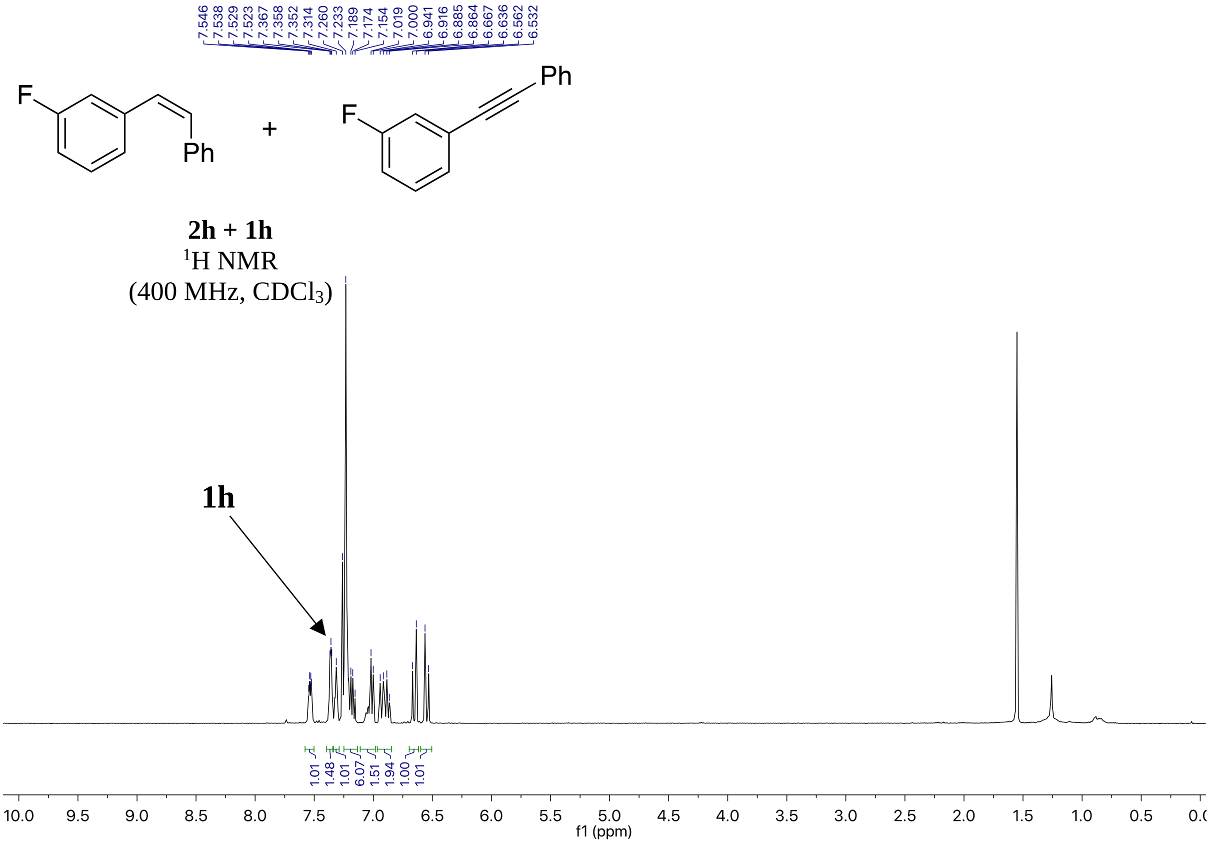

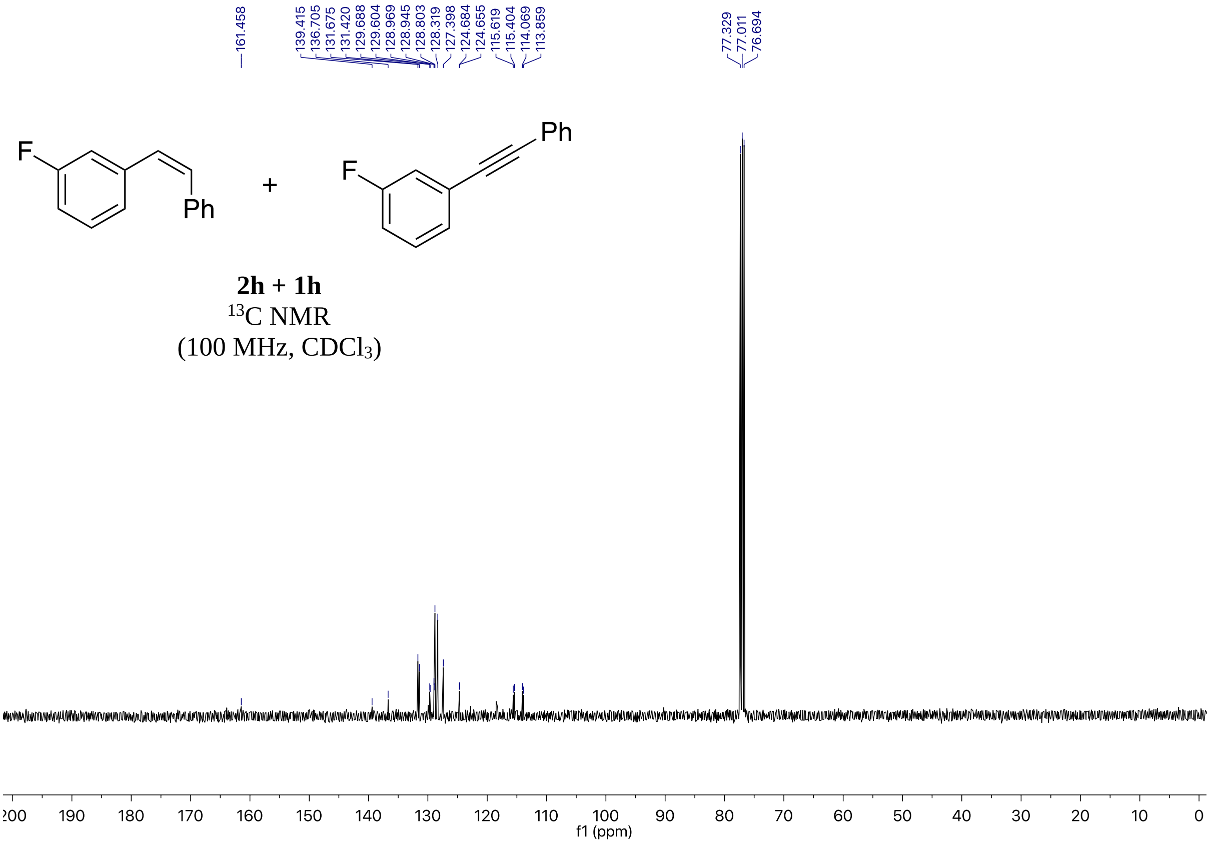

(*Z*)-1-Chloro-3-styrylbenzene (2i)

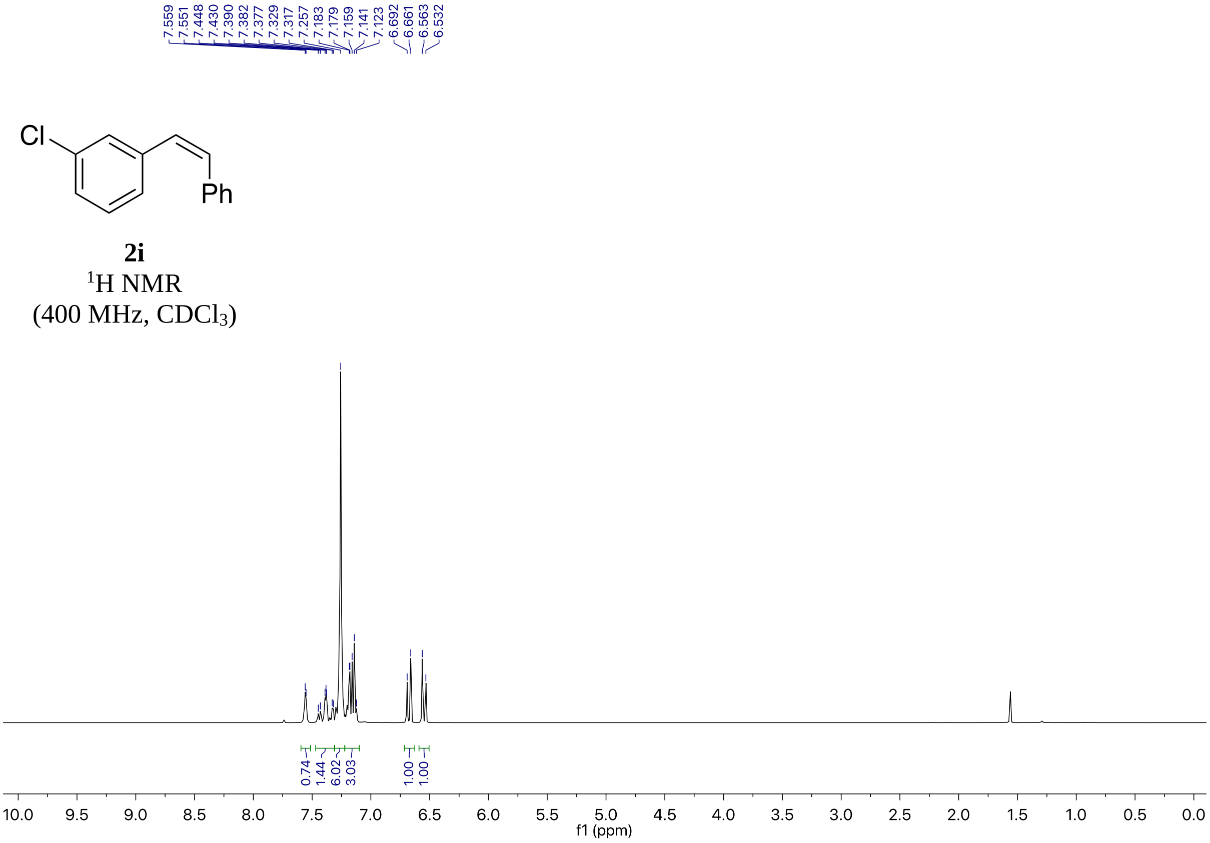

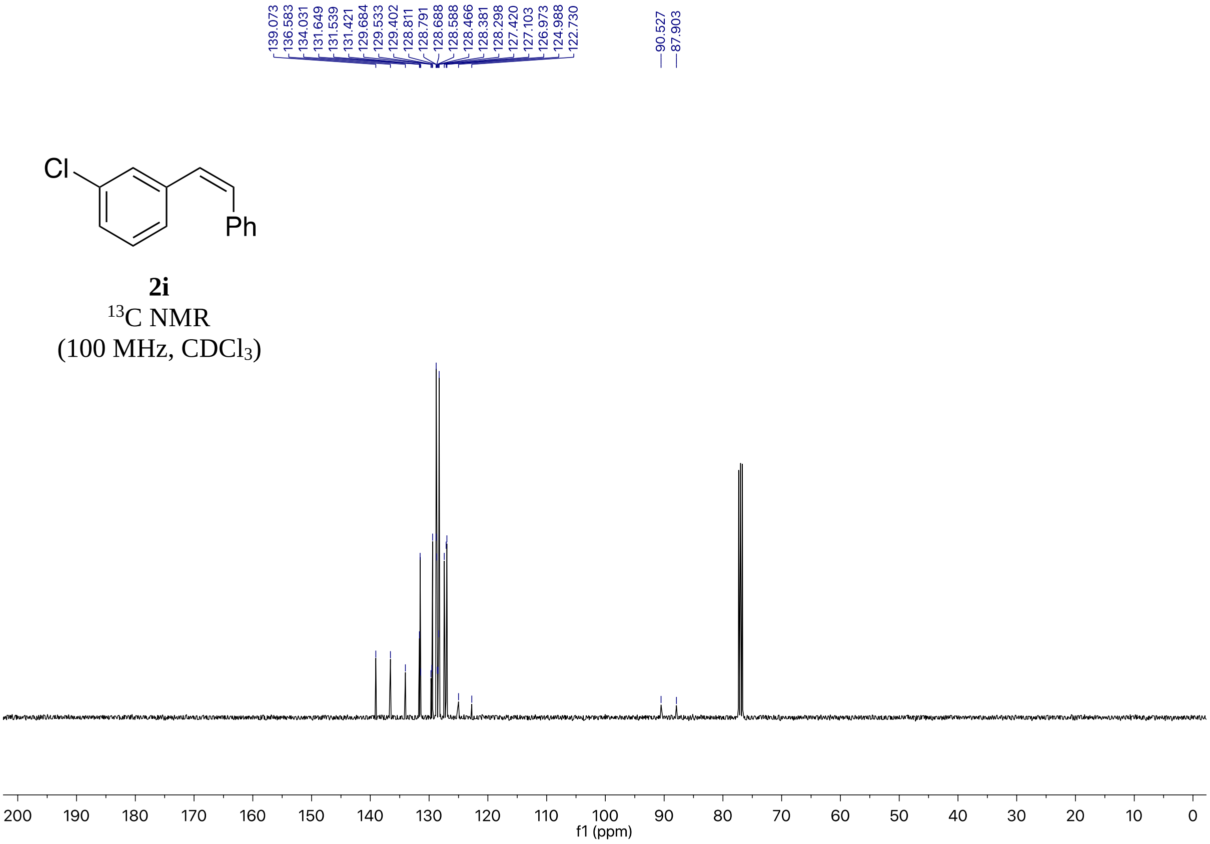

(*Z*)-1-Bromo-3-styrylbenzene (2j)

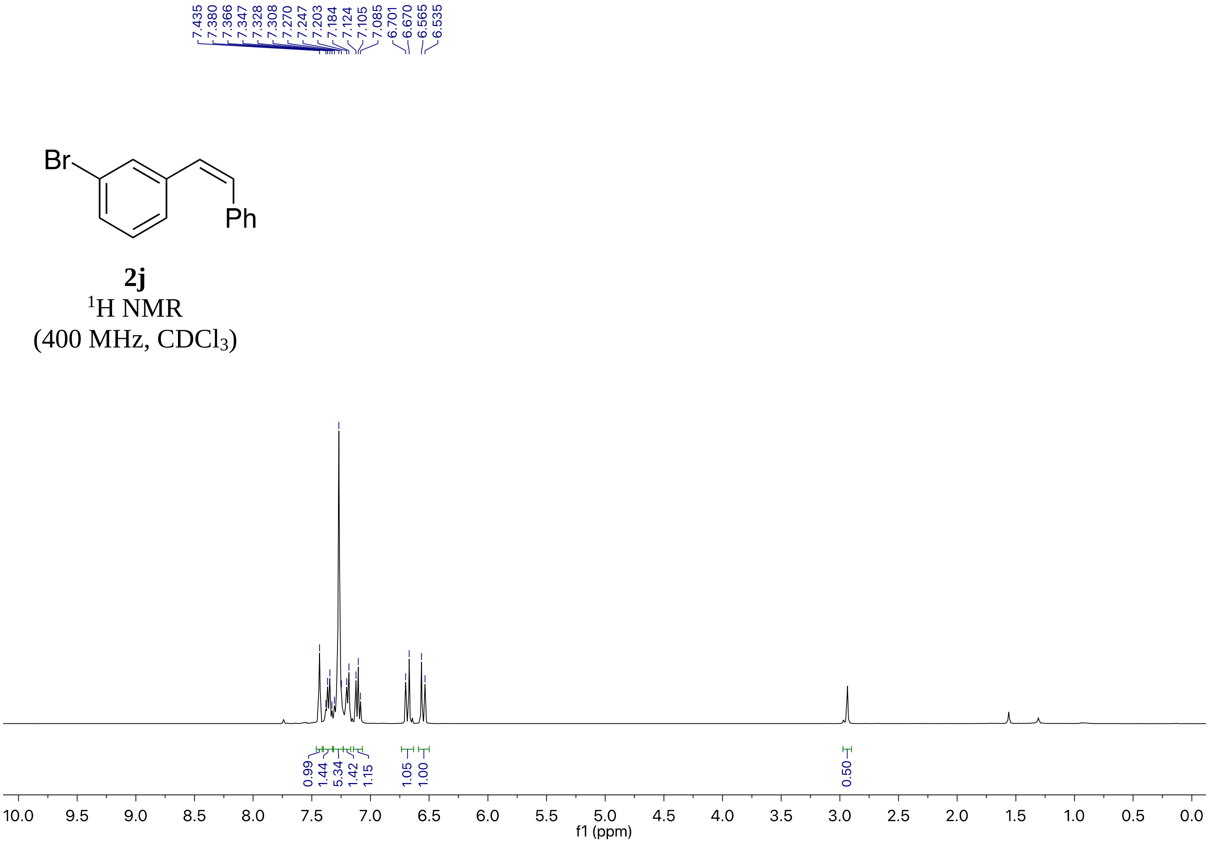

2j
^{1}H NMR
(400 MHz, $CDCl_3$)

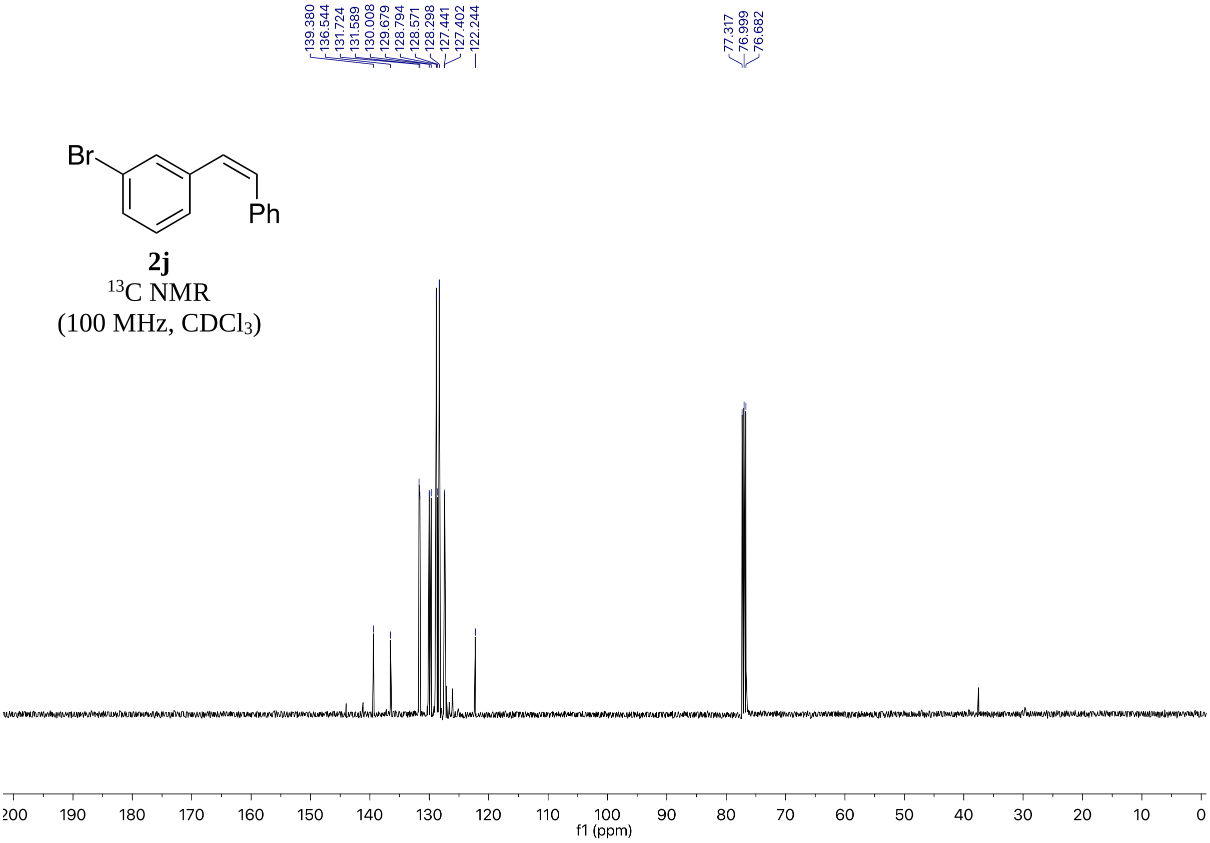

2j
^{13}C NMR
(100 MHz, $CDCl_3$)

(*Z*)-1-Fluoro-2-styrylbenzene (2k)

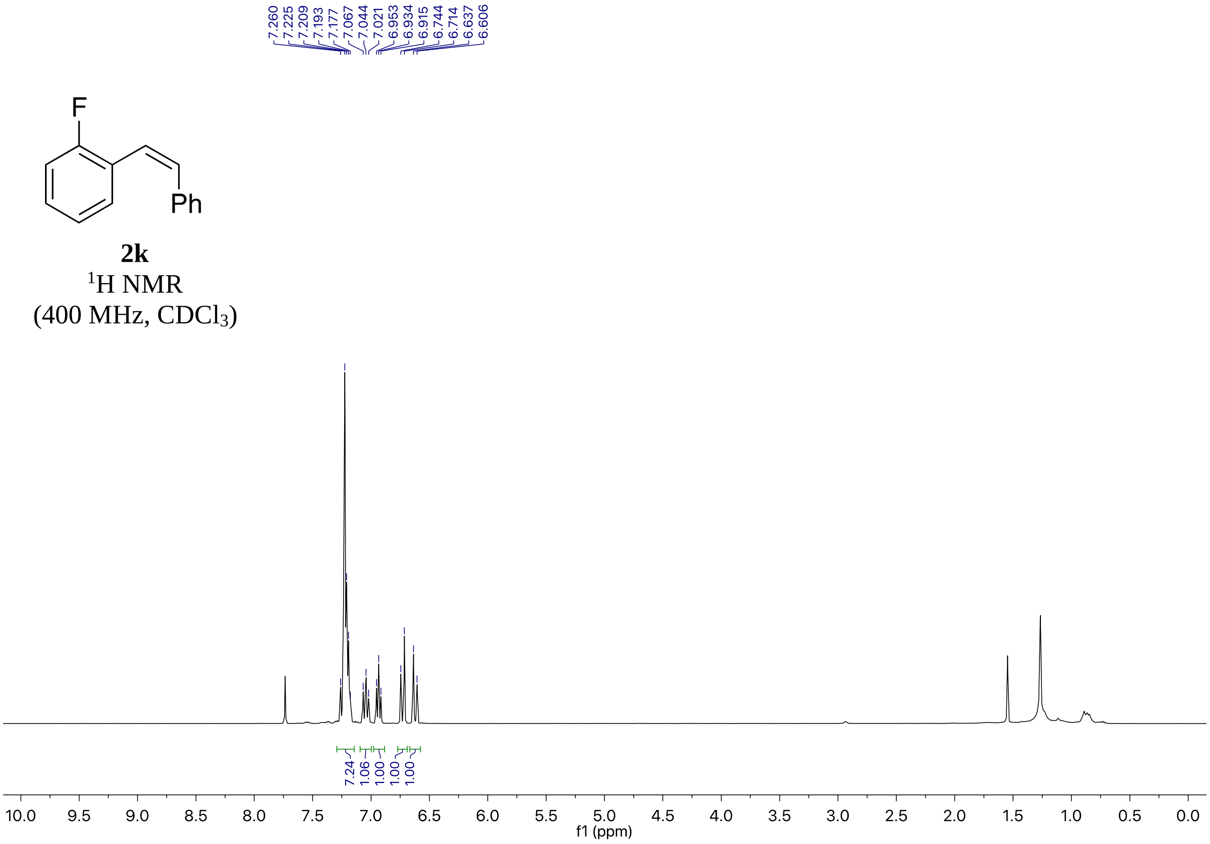

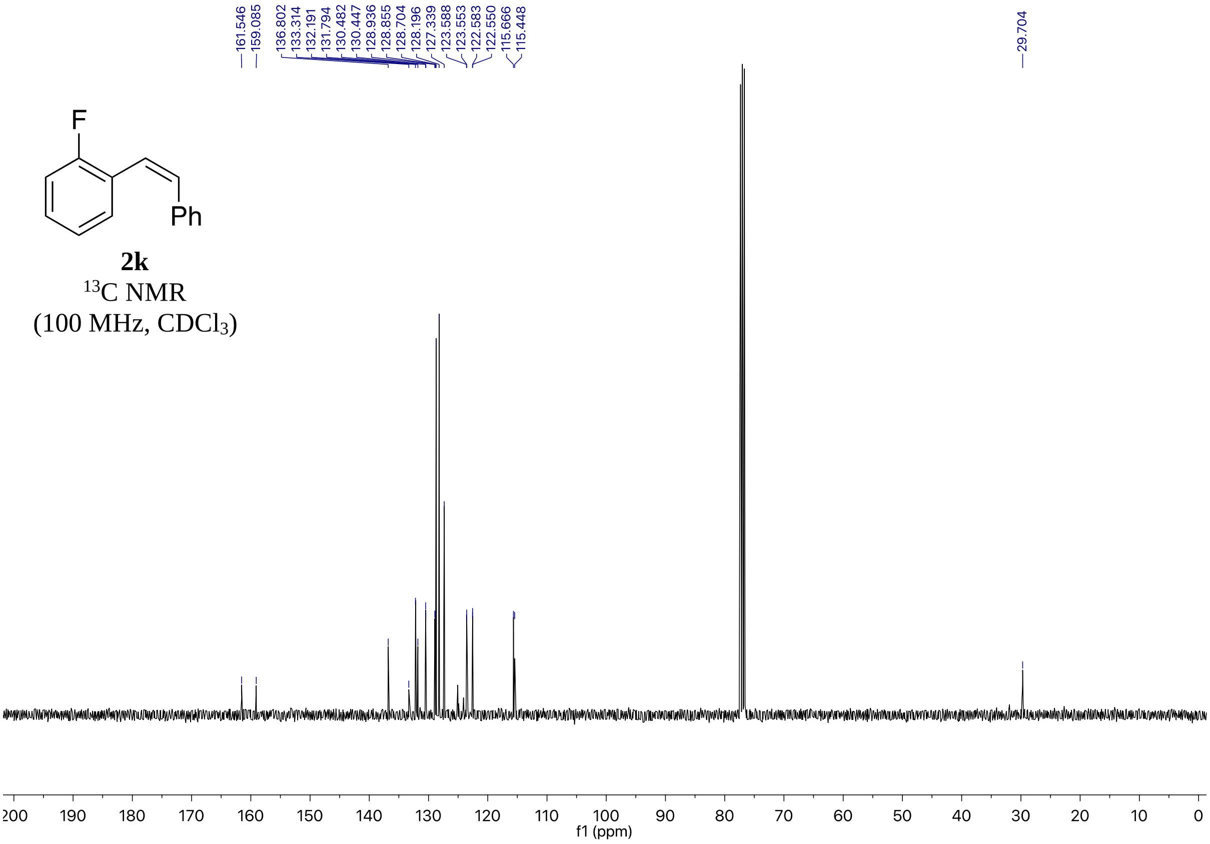

(*Z*)-1-Bromo-2-styrylbenzene (2l)

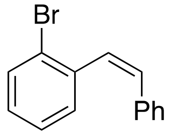

2l
^{1}H NMR
(400 MHz, $CDCl_3$)

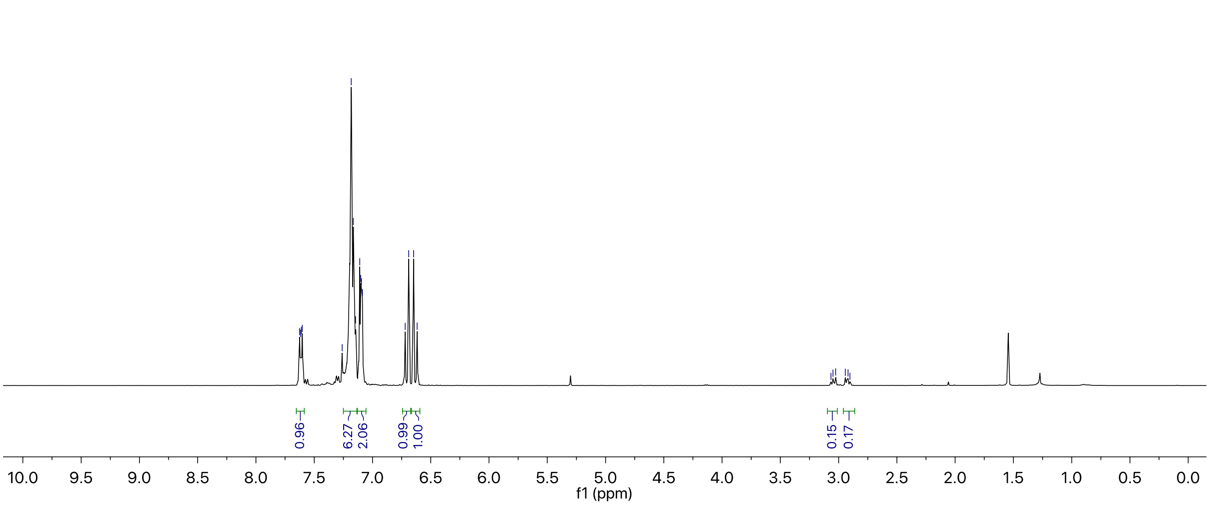

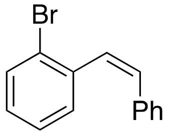

2l
^{13}C NMR
(100 MHz, $CDCl_3$)

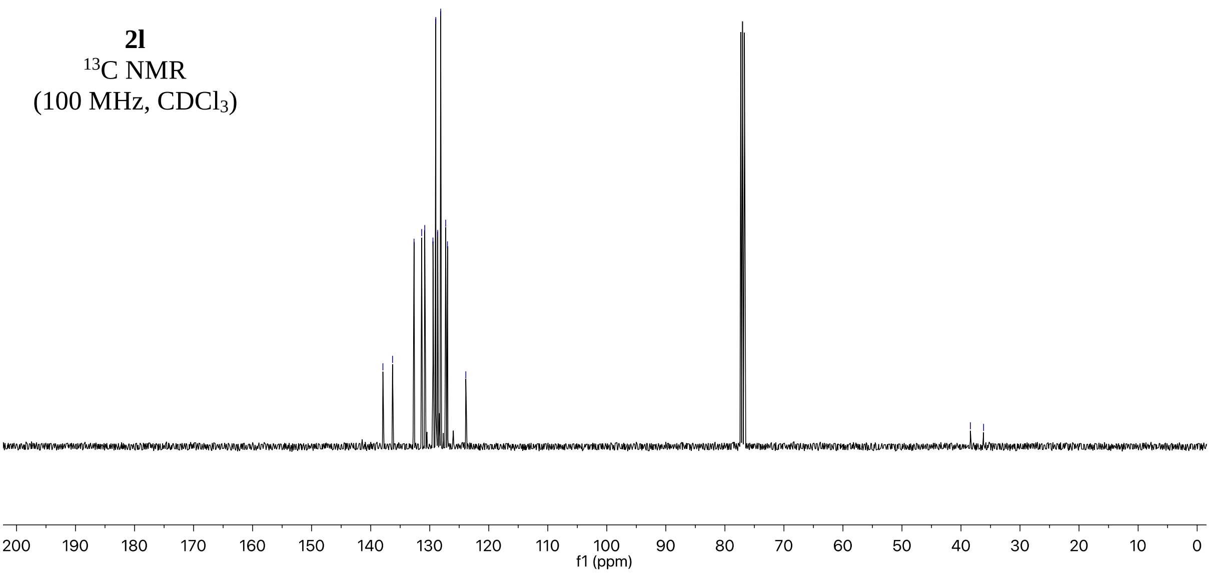

(*Z*)-1-Styrylnaphthalene (2m)

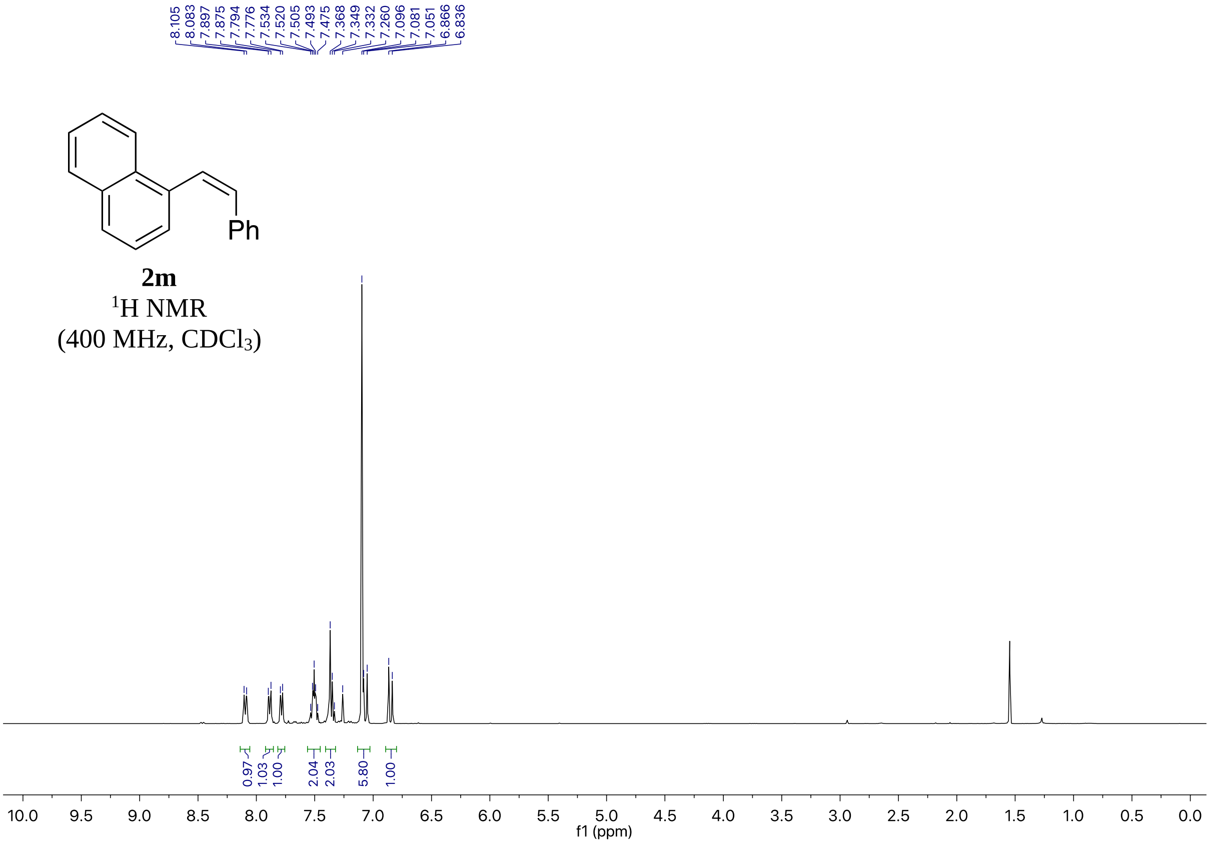

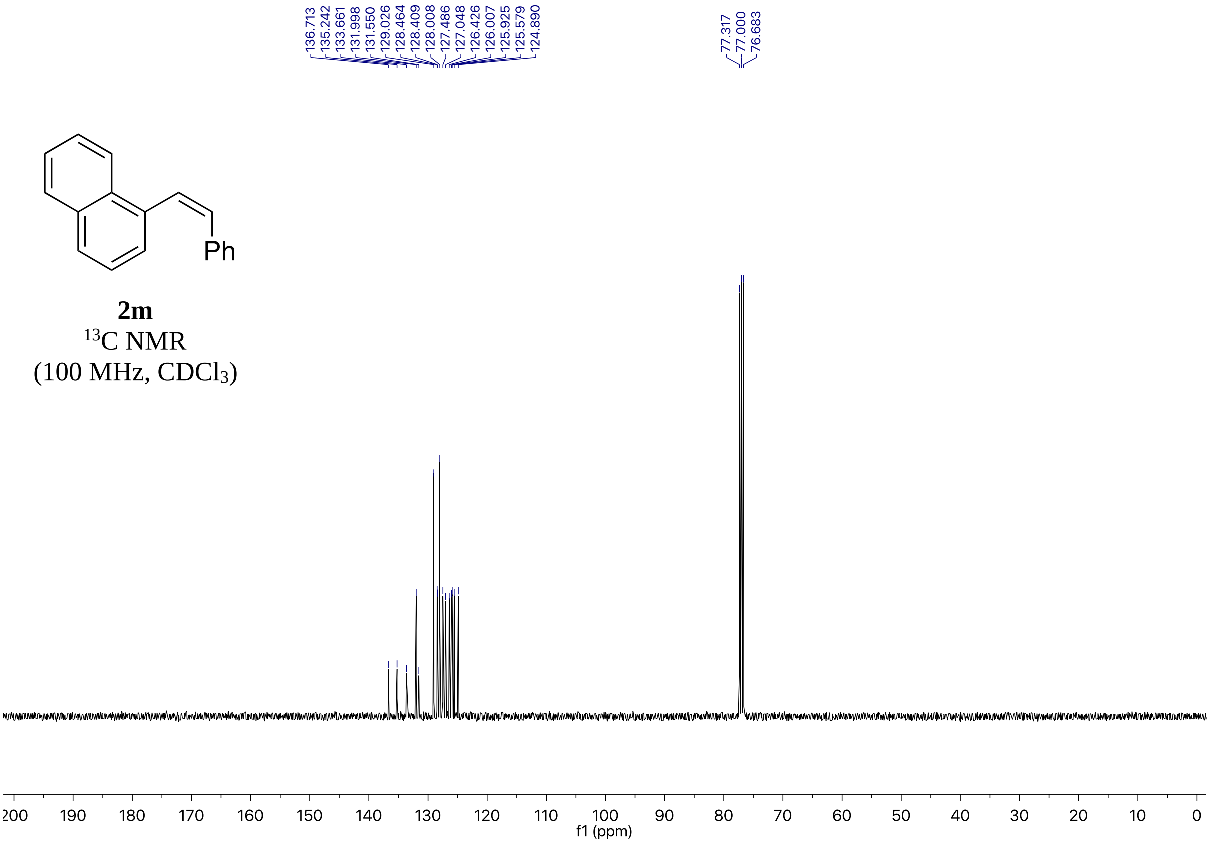

(*Z*)-2,4-Dichloro-1-styrylbenzene (2n)

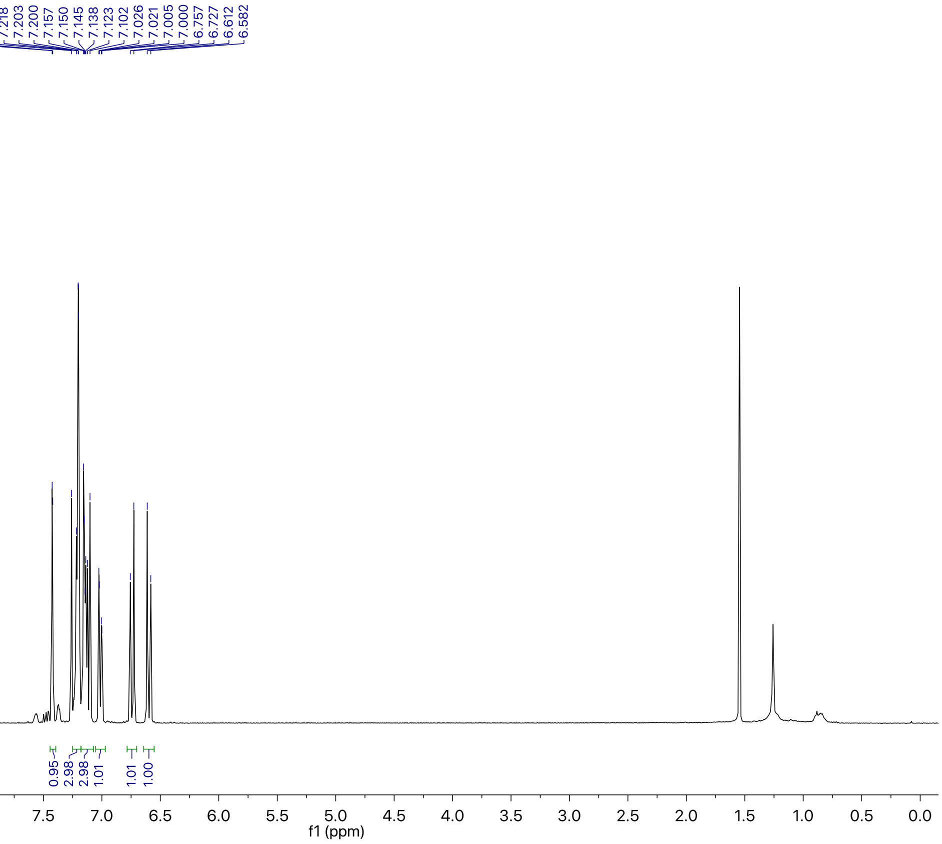

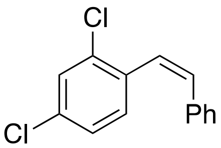

2n
^{1}H NMR
(400 MHz, $CDCl_3$)

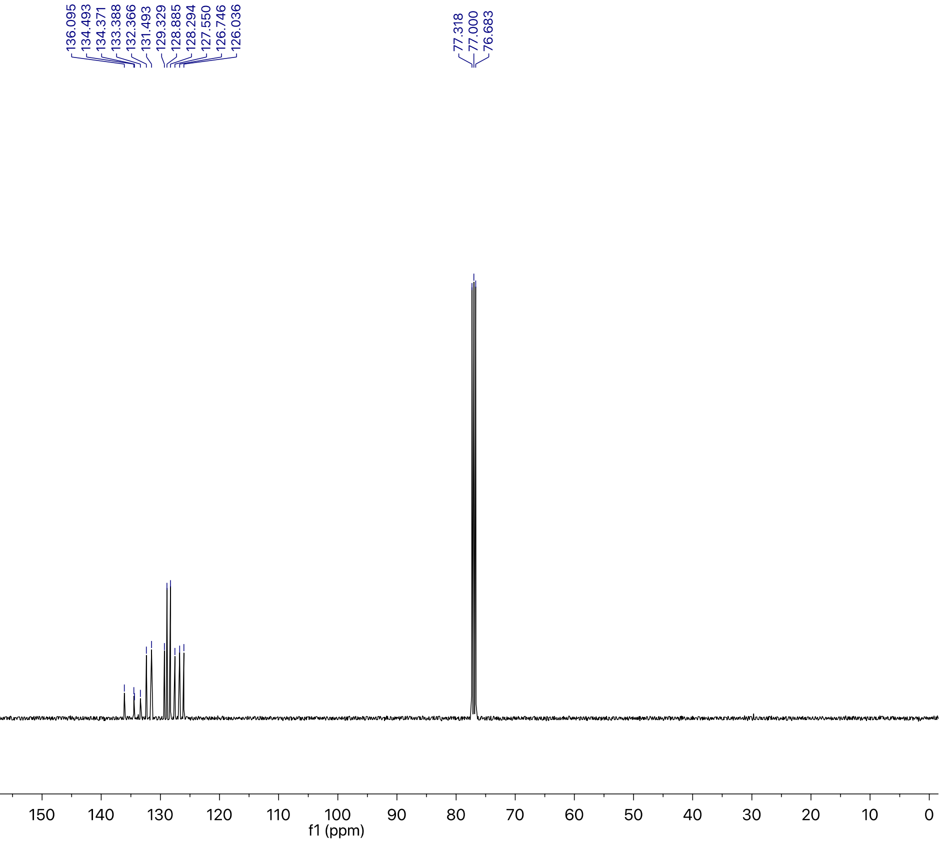

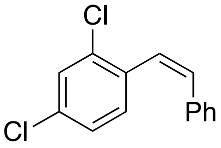

2n
^{13}C NMR
(100 MHz, $CDCl_3$)

(*Z*)-1,2,3,4,5-Pentafluoro-6-styrylbenzene (2o)

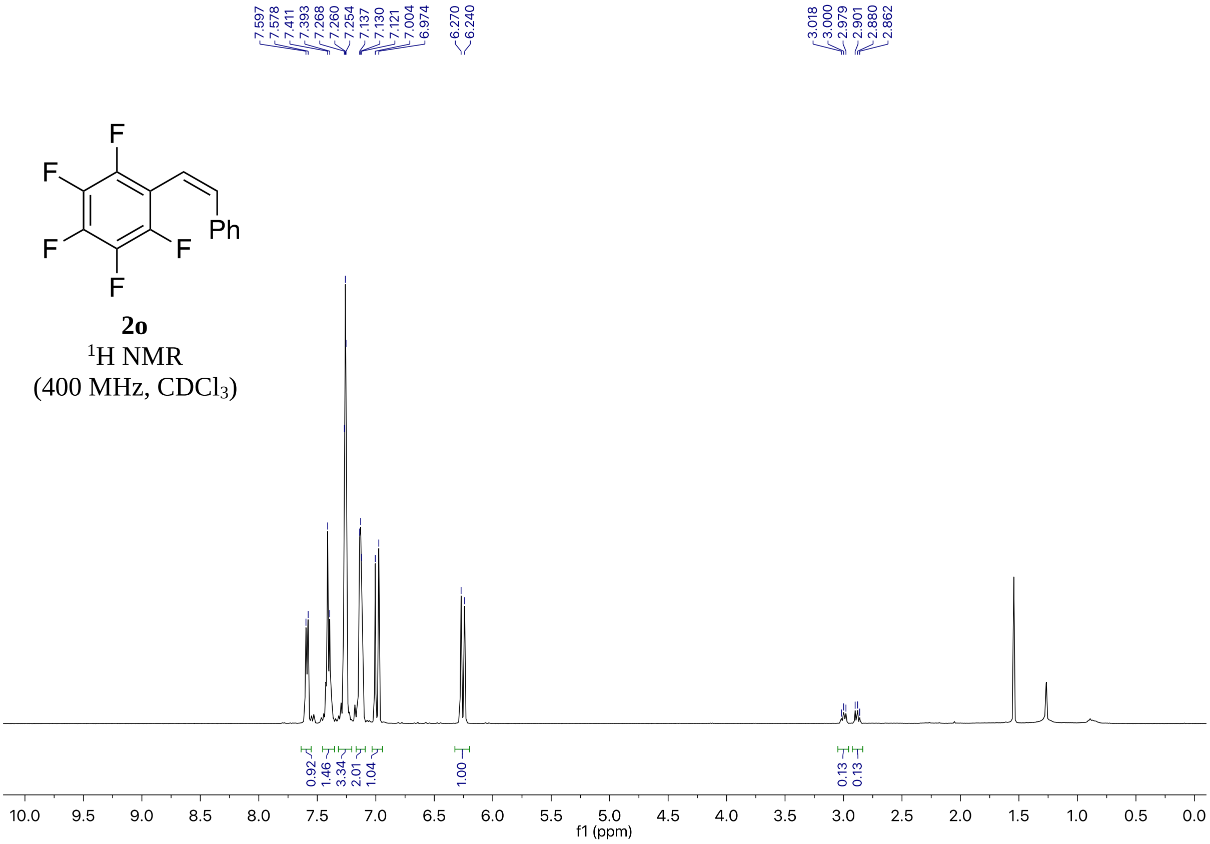

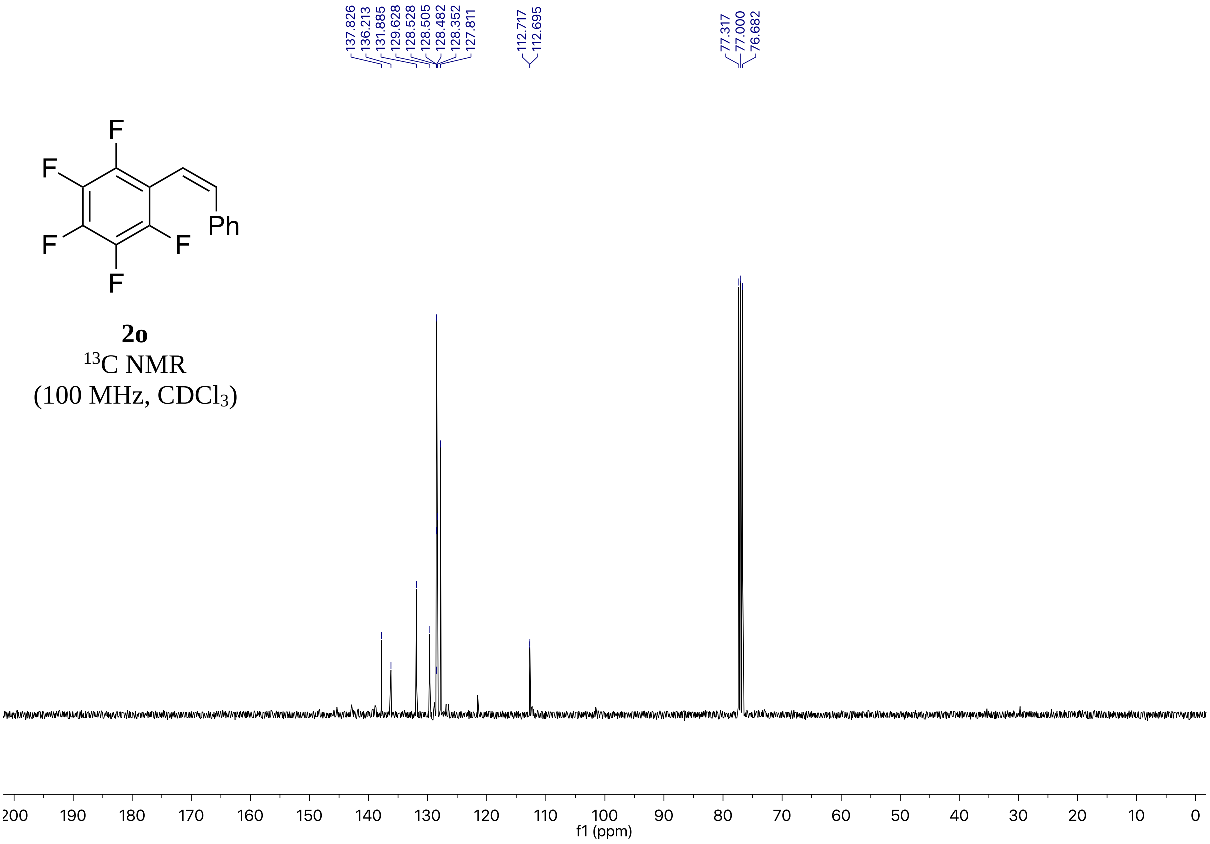

(*Z*)-3-(2-(Thiophen-3-yl)vinyl)pyridine (2p)

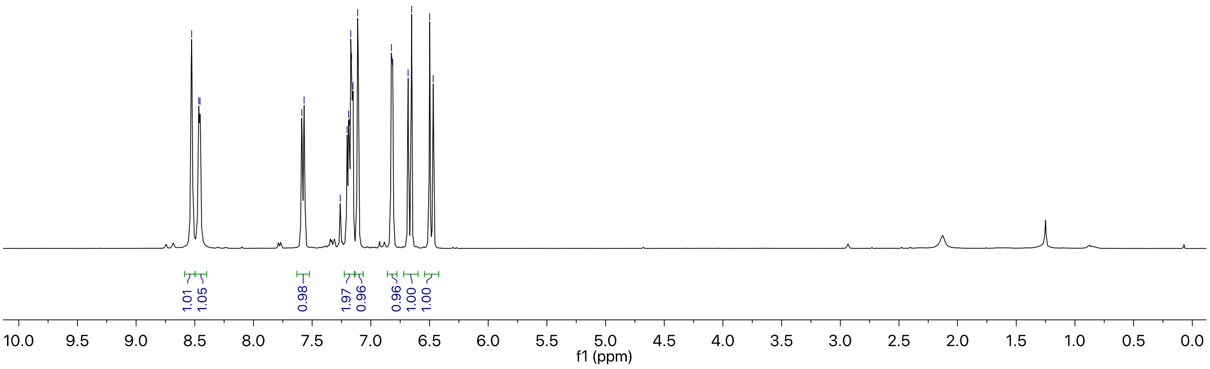

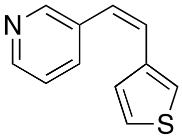

2p
^{1}H NMR
(400 MHz, $CDCl_3$)

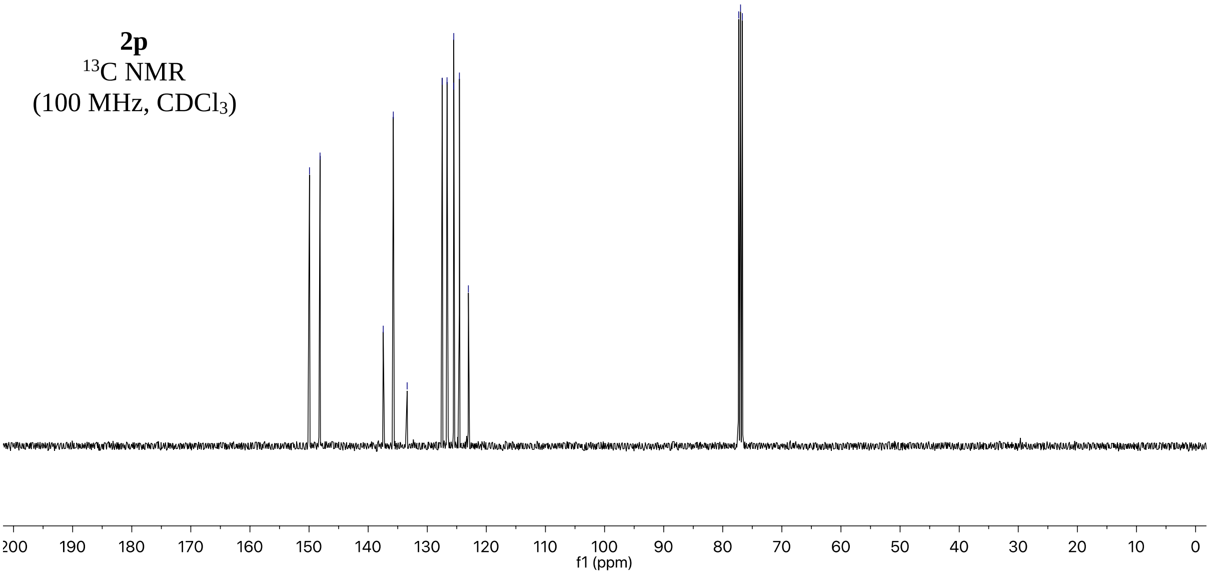

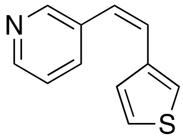

2p
^{13}C NMR
(100 MHz, $CDCl_3$)

(*Z*)-4-(4-(2-Hydroxyethyl)styryl)benzonitrile (2q) and (1q)

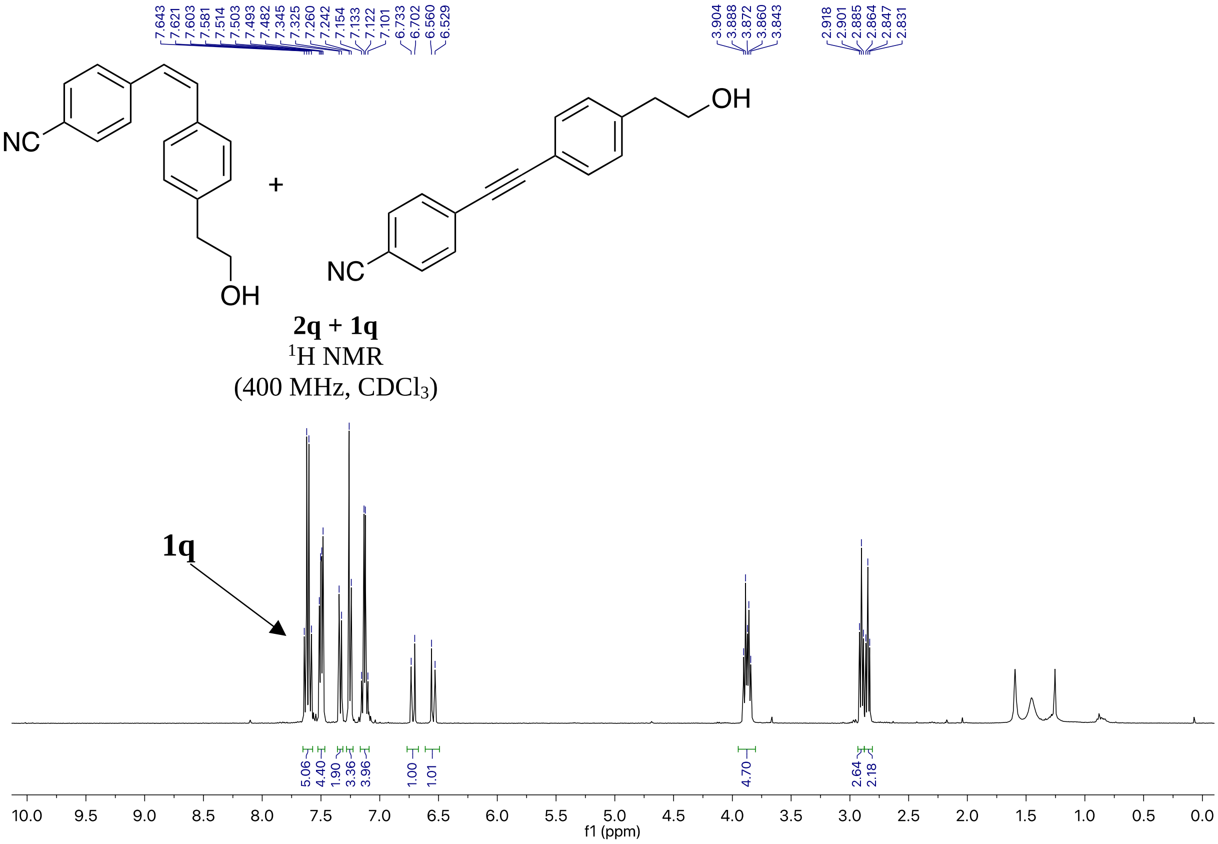

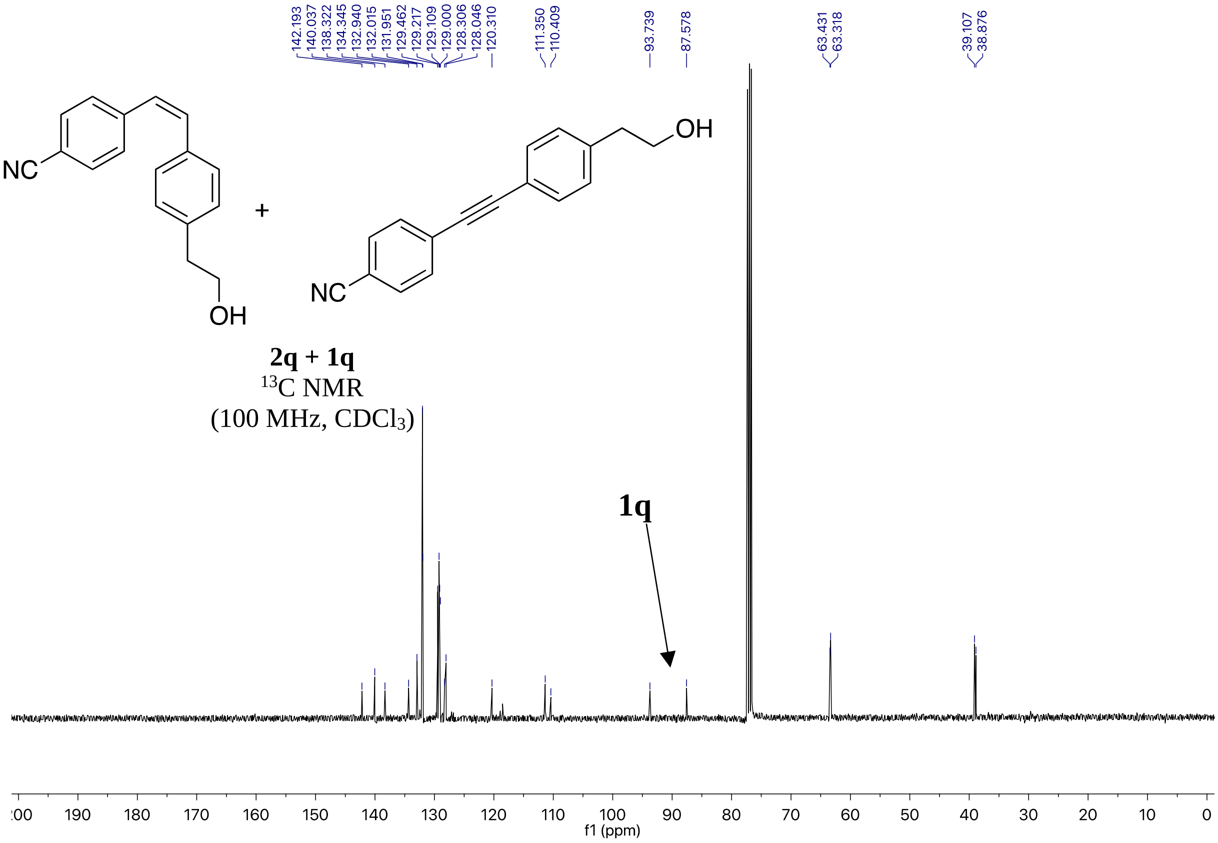

(*Z*)-4-(3-Hydroxyprop-1-en-1-yl)benzonitrile (2r)

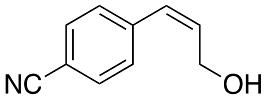

2r
^{1}H NMR
(400 MHz, $CDCl_3$)

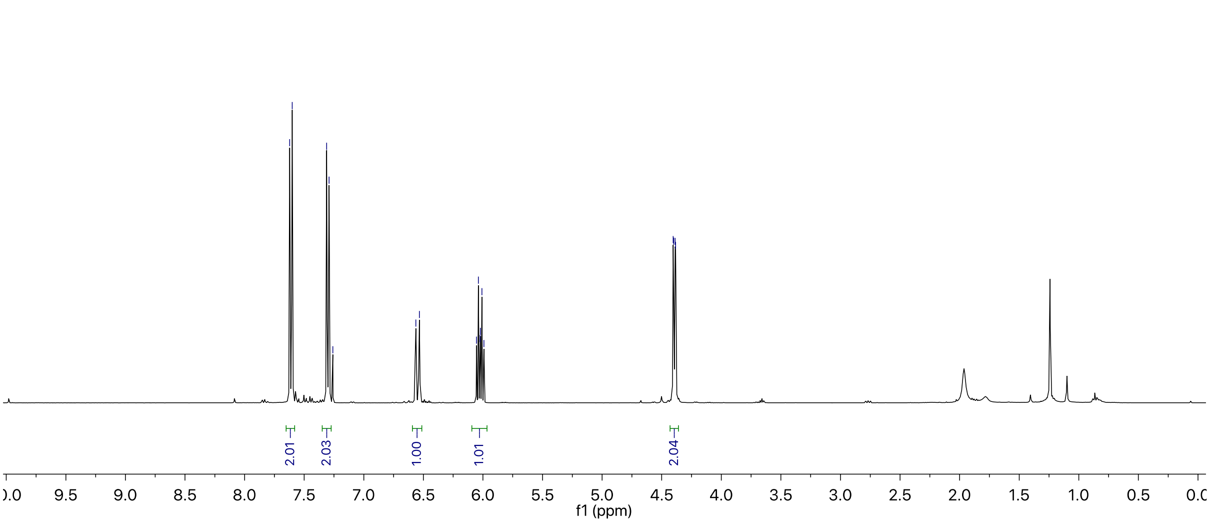

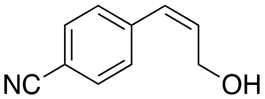

2r
^{13}C NMR
(100 MHz, $CDCl_3$)

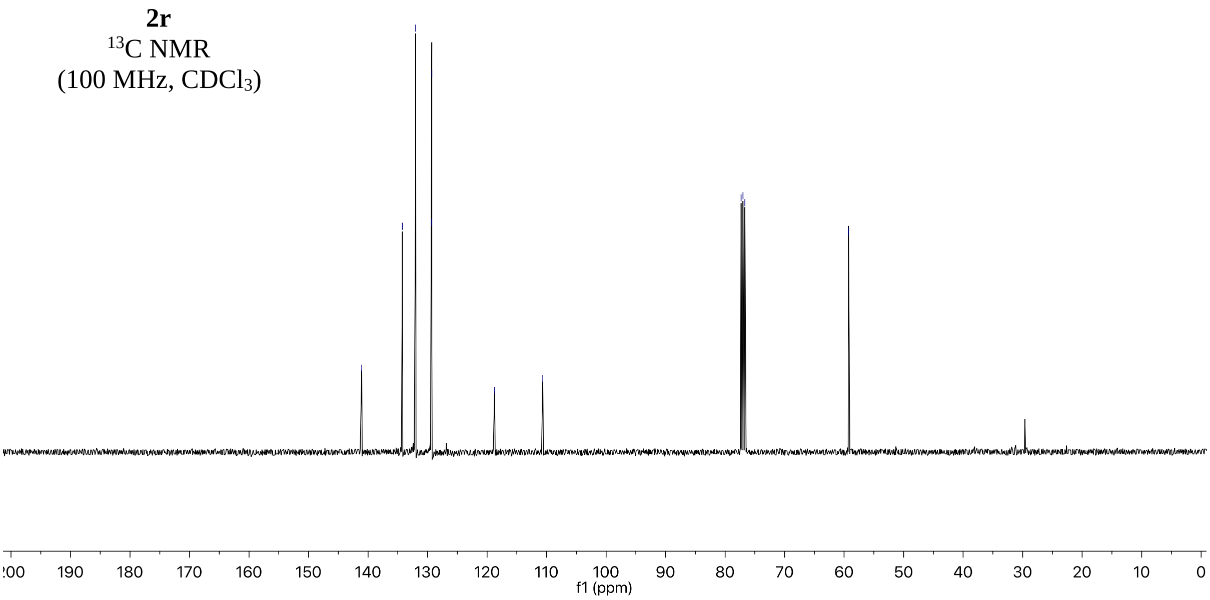

Methyl (*Z*)-3-phenylacrylate (2s)

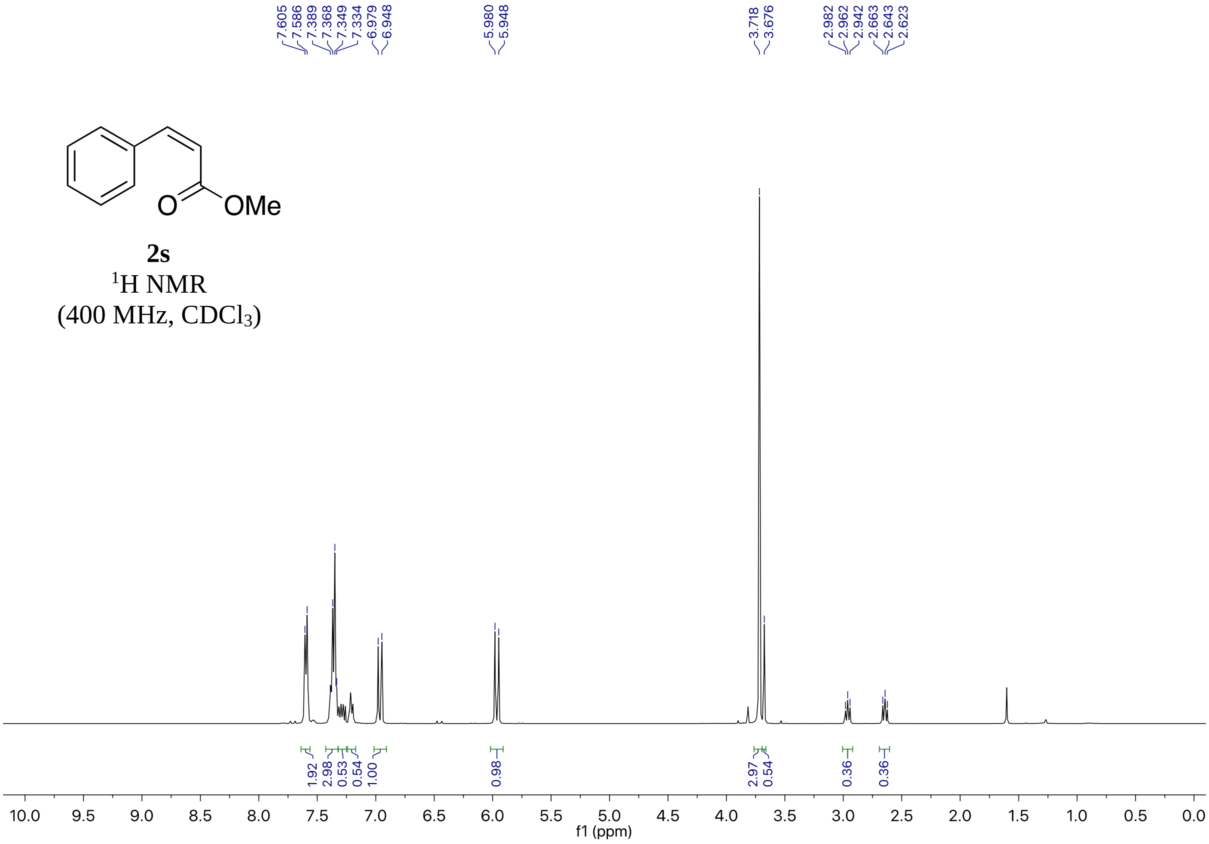

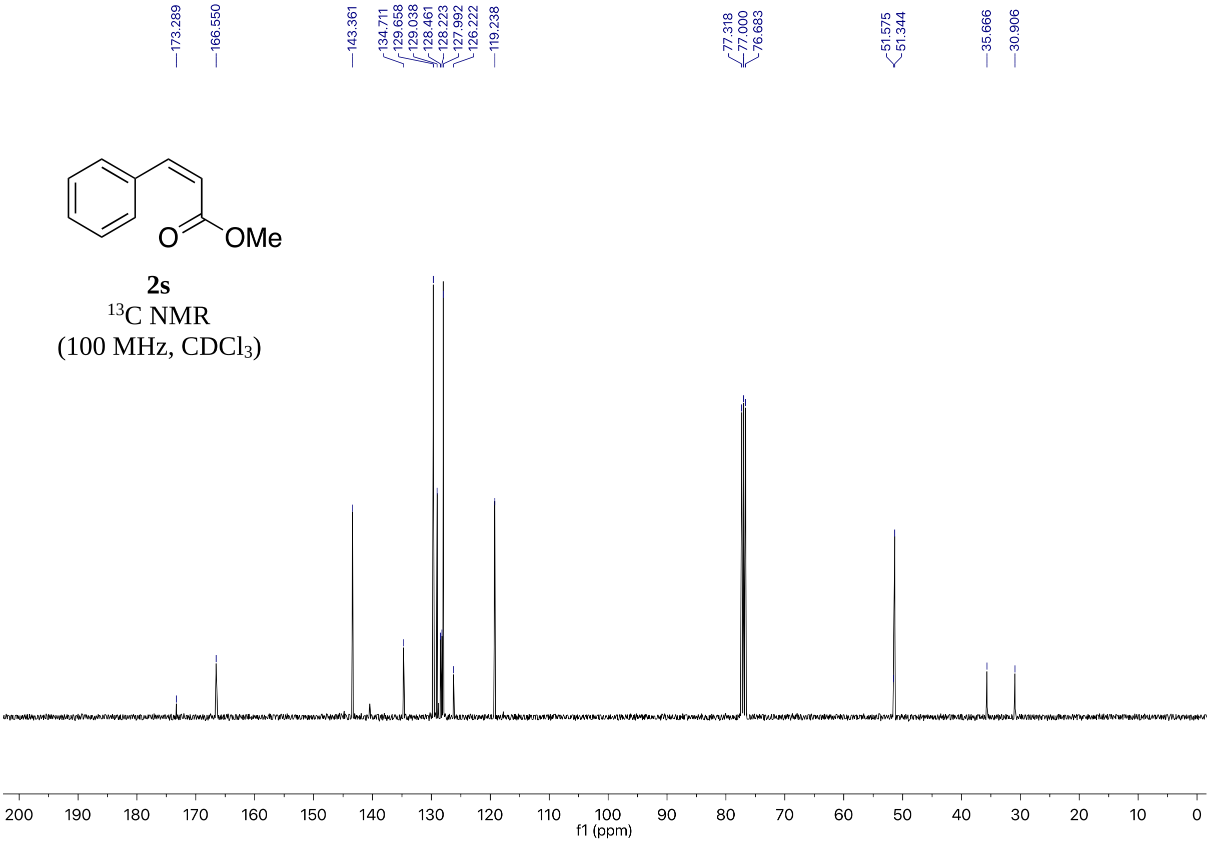

Methyl (*Z*)-3-(4-iodophenyl)acrylate (2t)

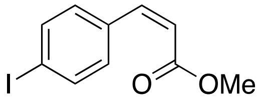

2t
^{1}H NMR
(400 MHz, $CDCl_3$)

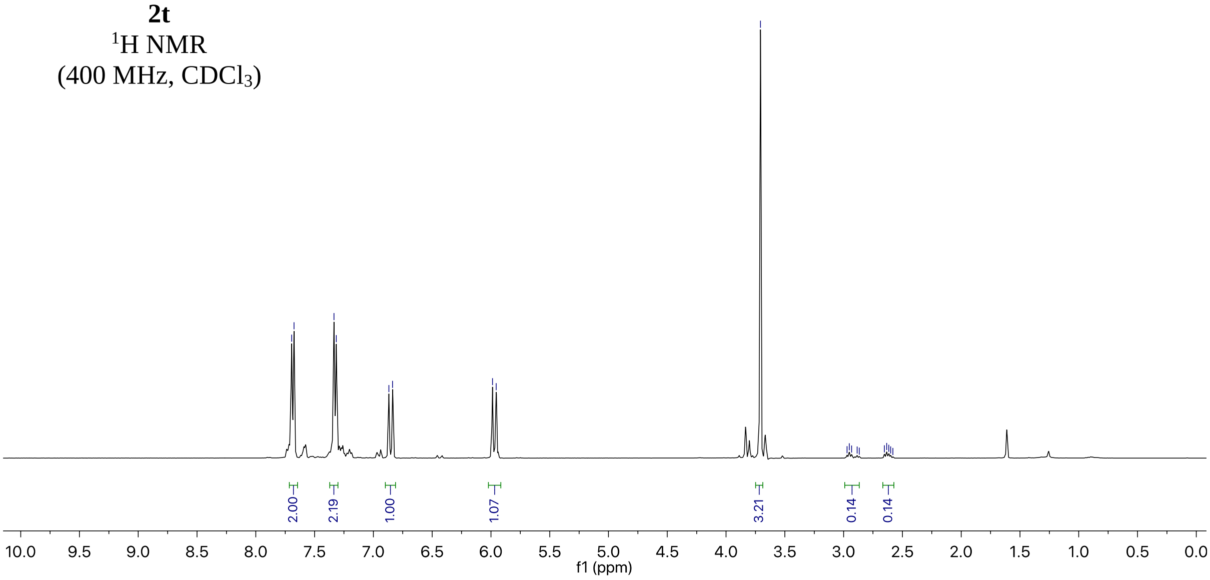

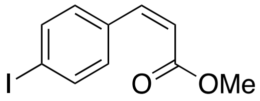

2t
^{13}C NMR
(100 MHz, $CDCl_3$)

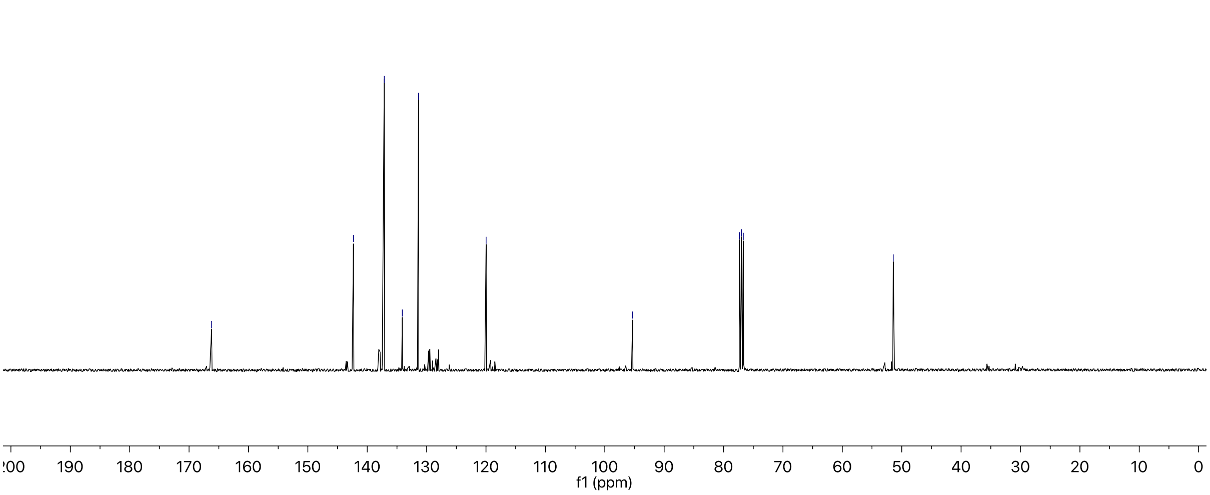

Methyl (*Z*)-hept-2-enoate (2u), (1u) and (4u)

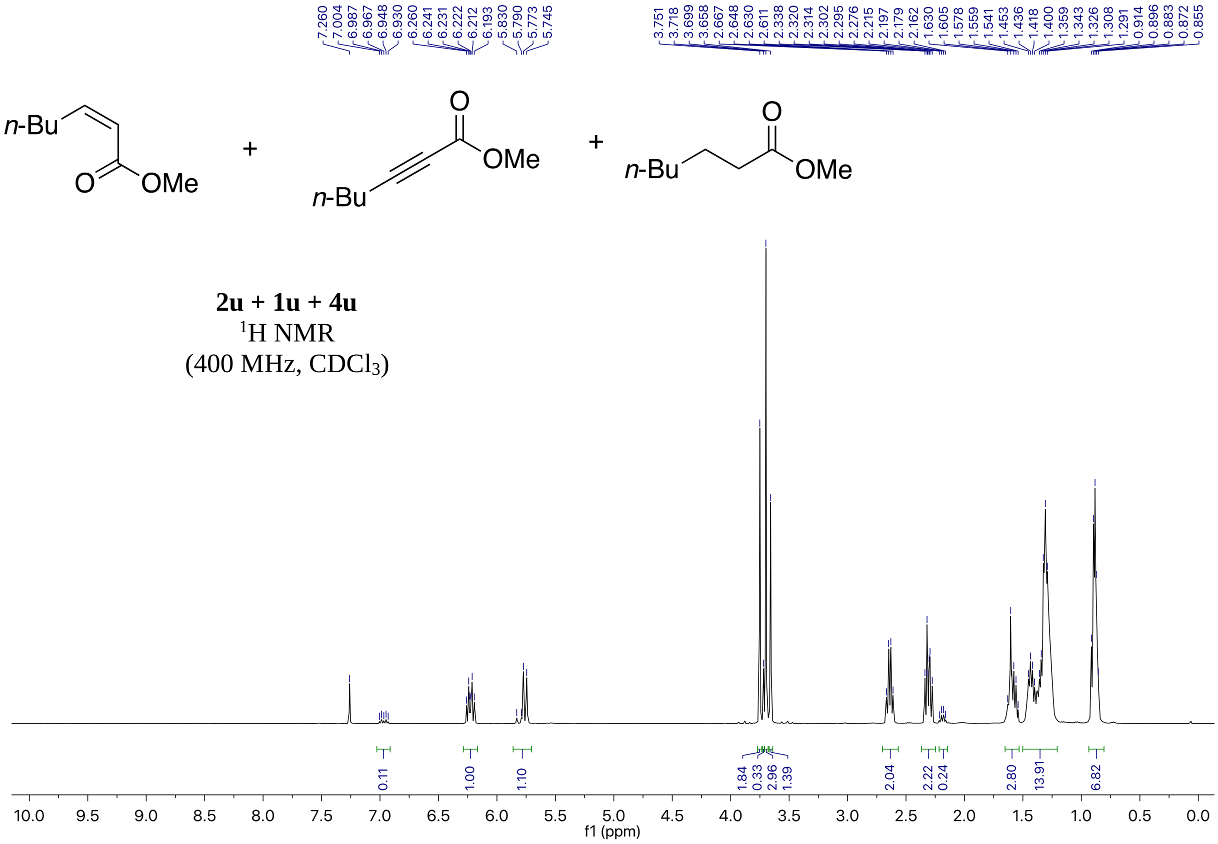

^{1}H NMR of the reaction crude. The purified mixture doesn't correspond to the crude due to the volatile nature of some of the compounds.

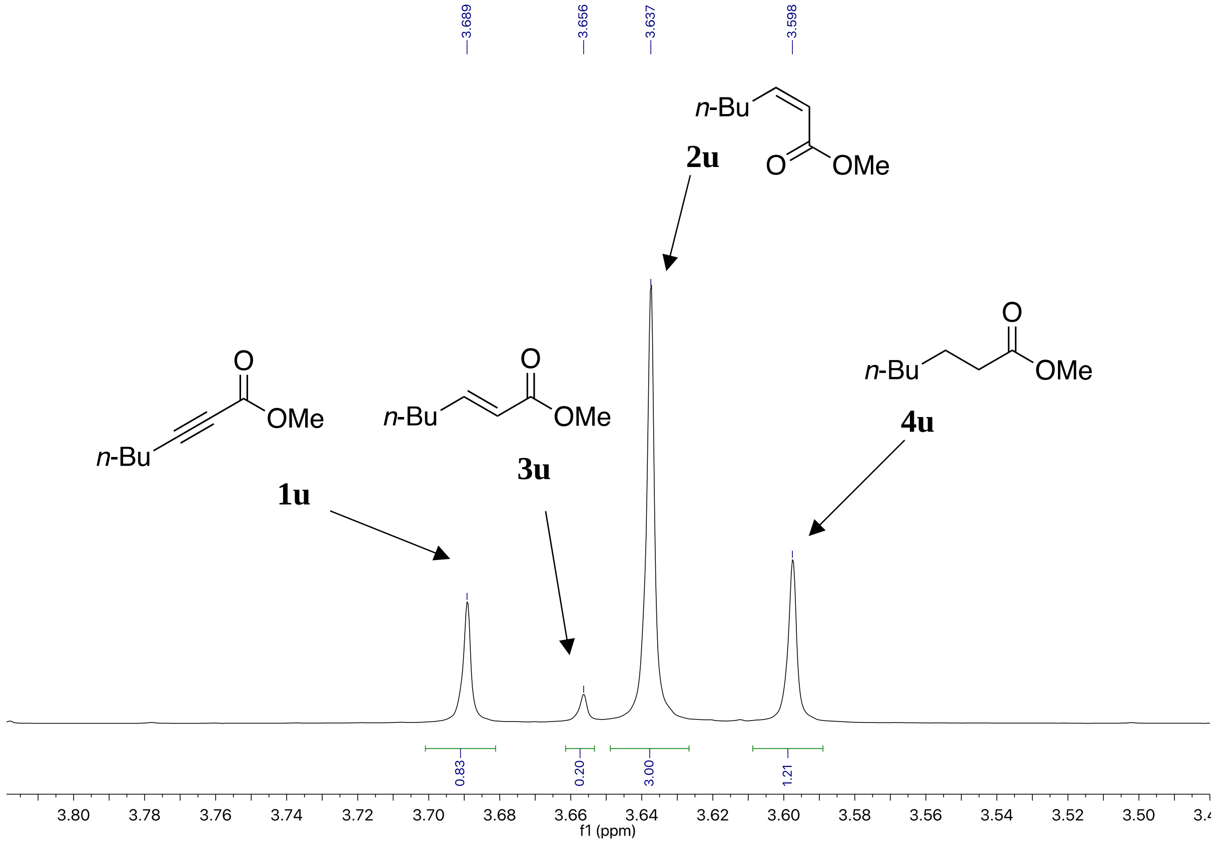

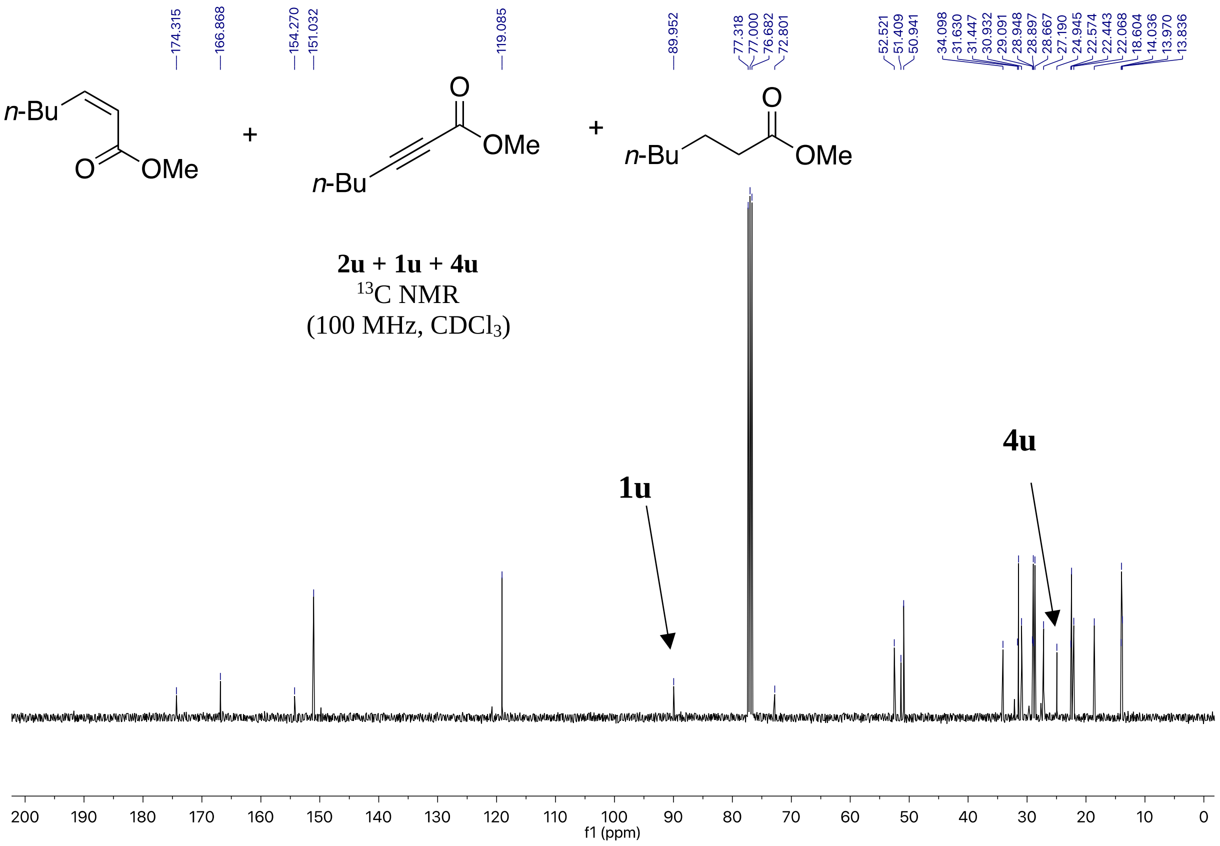
n-Bu
OMe
+
n-Bu
OMe
+
n-Bu
OMe
2u + 1u + 4u
^{13}C NMR
(100 MHz, $CDCl_3$)
1u
4u
f1 (ppm)

((1*R*,8*S*,9*s*,*Z*)-Bicyclo[6.1.0]non-4-en-9-yl)methanol (2v)

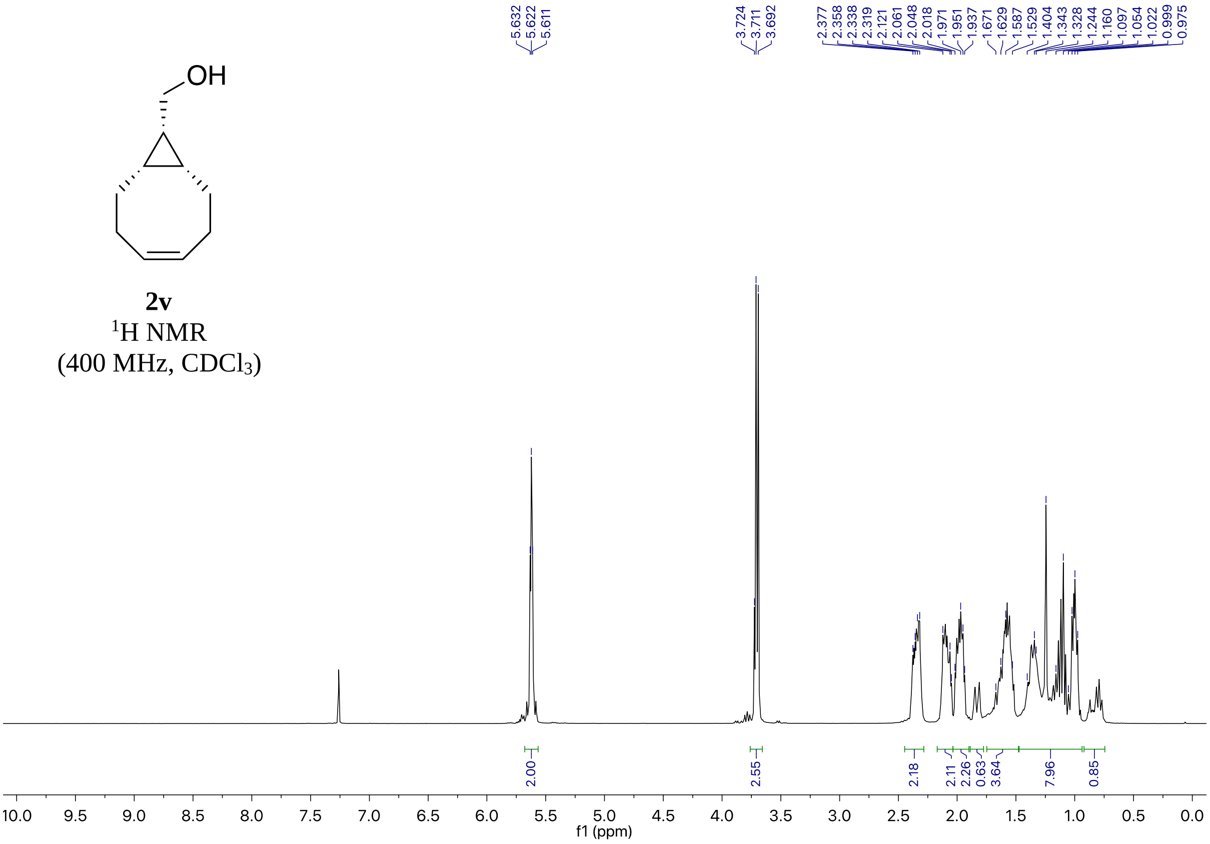

2v
^{1}H NMR
(400 MHz, $CDCl_3$)

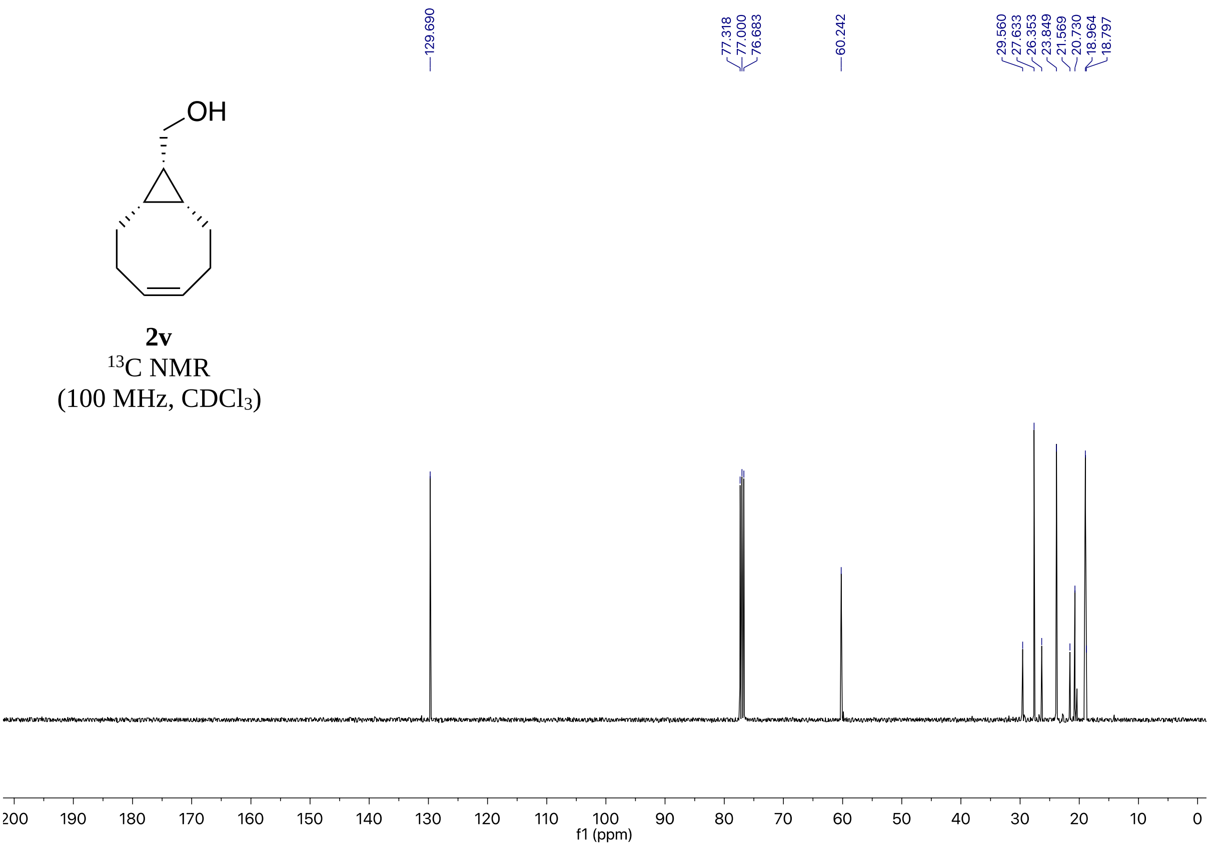

2v
^{13}C NMR
(100 MHz, $CDCl_3$)

(*Z*)-Dec-5-en-1-ol (2w)

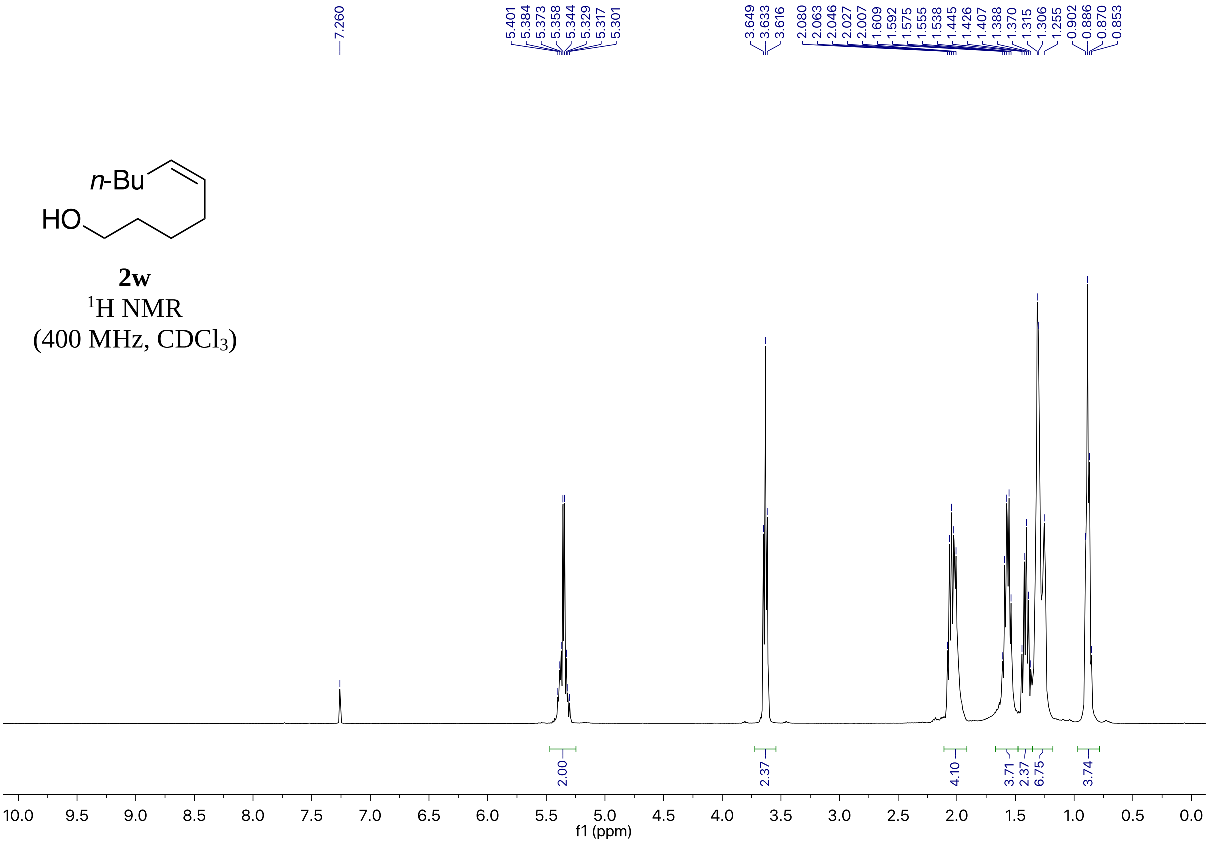

2w
^{1}H NMR
(400 MHz, $CDCl_3$)

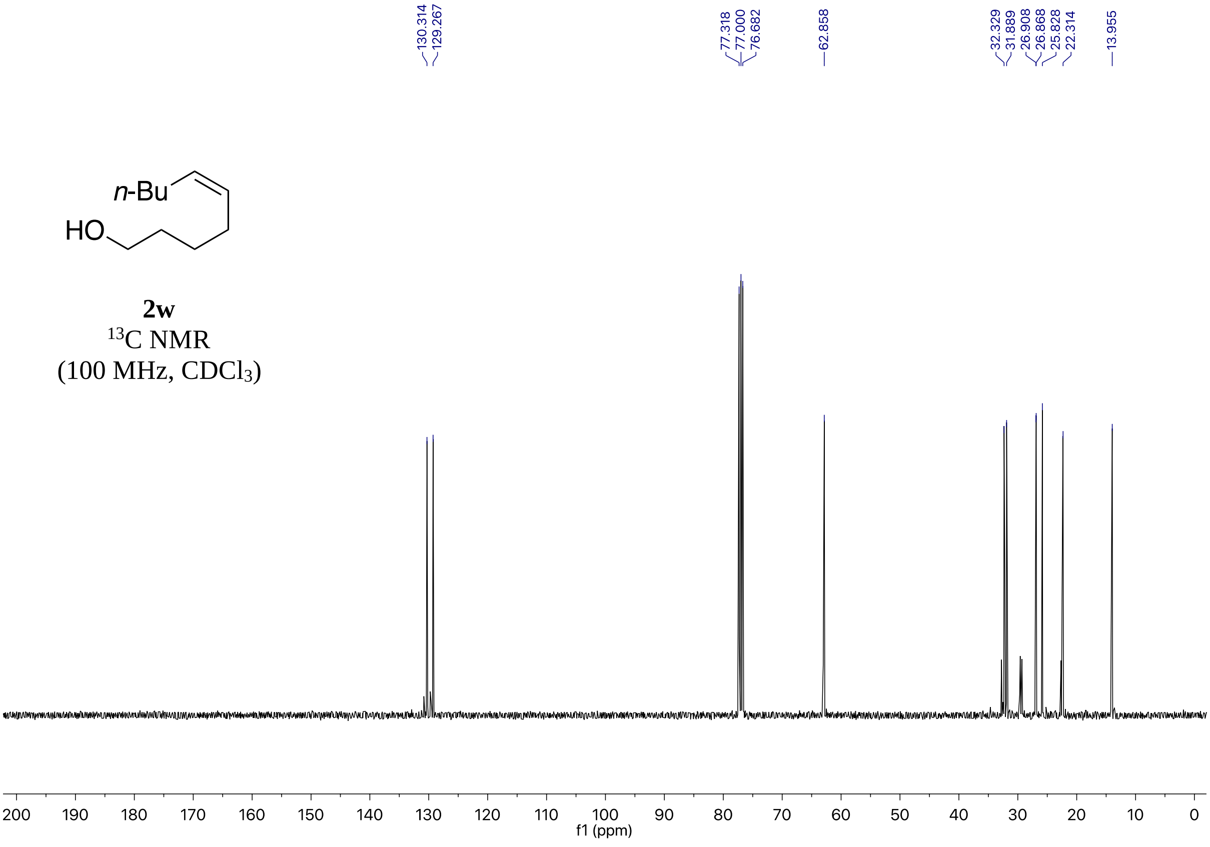

2w
^{13}C NMR
(100 MHz, $CDCl_3$)

(*Z*)-dec-5-enal (2x)

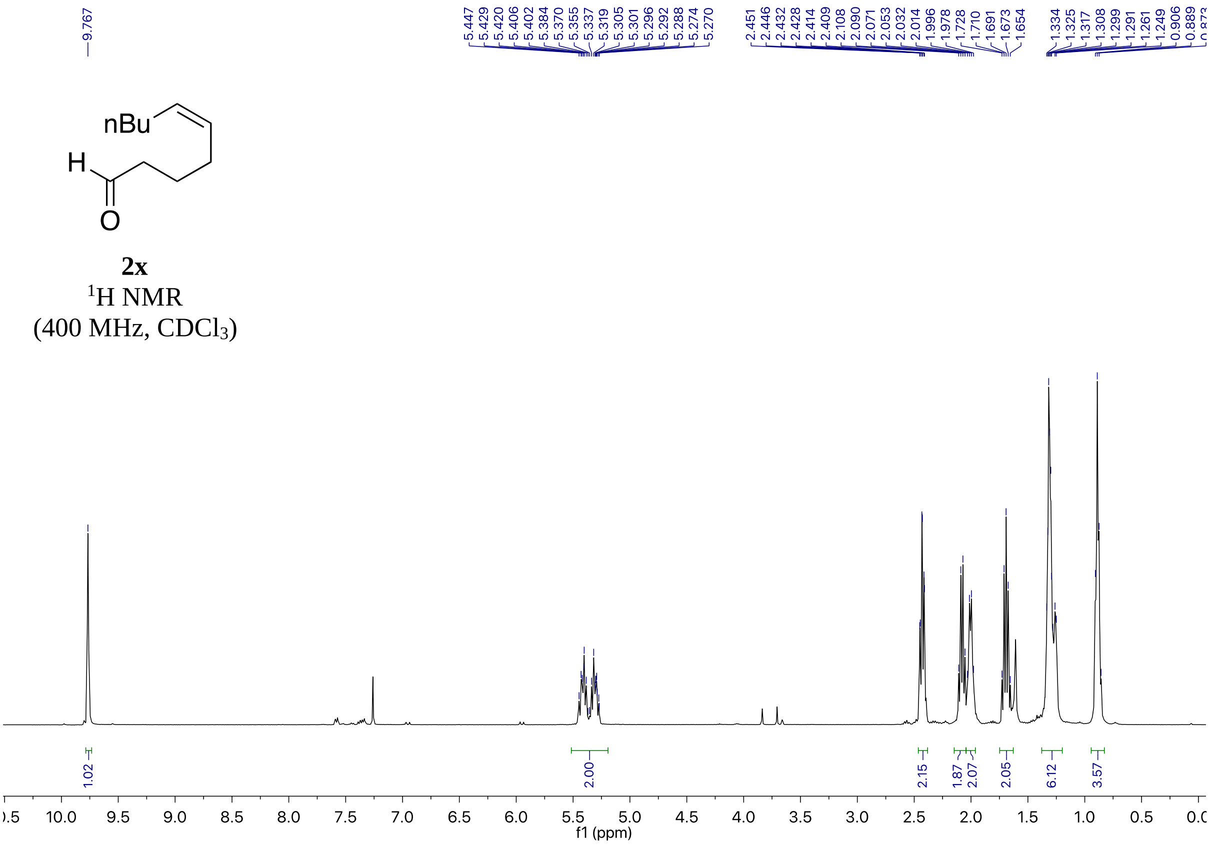

2x
^{1}H NMR
(400 MHz, $CDCl_3$)

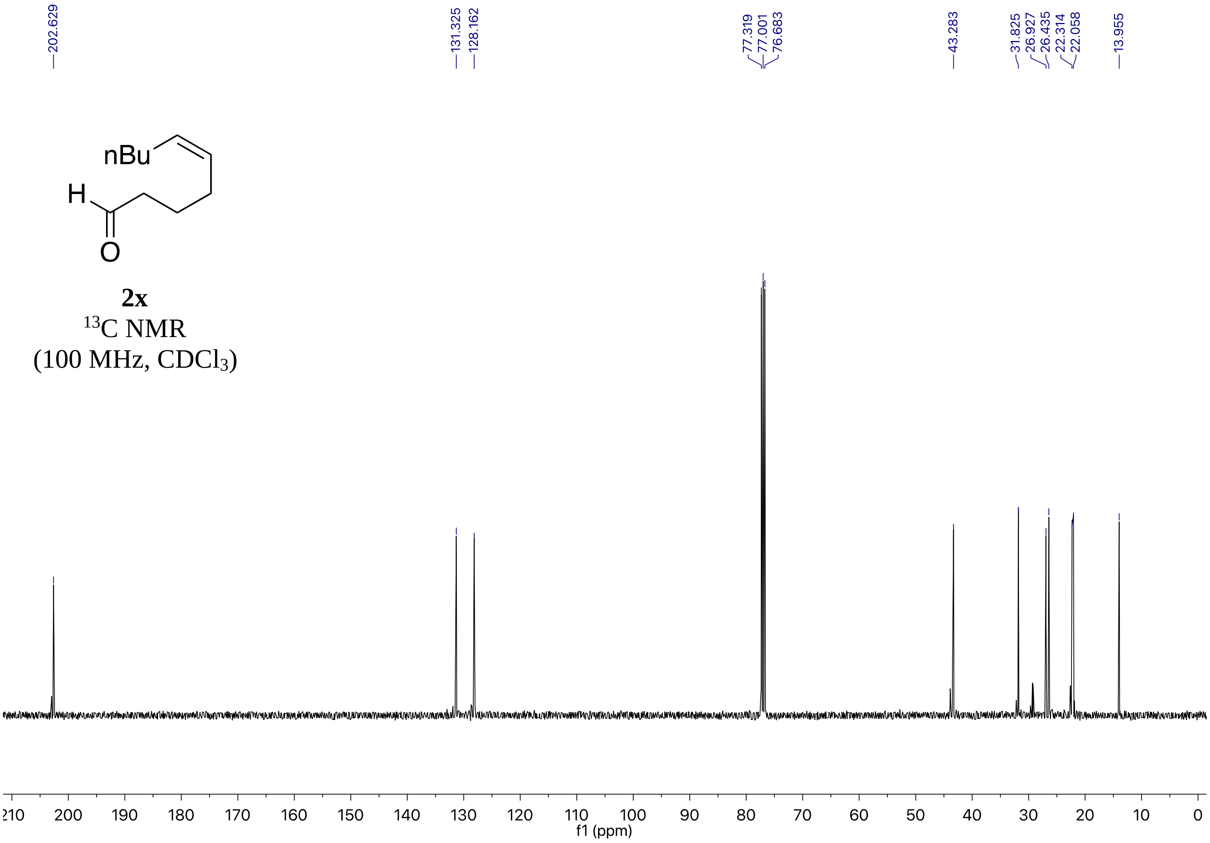

2x
^{13}C NMR
(100 MHz, $CDCl_3$)

Oleic acid (2y)

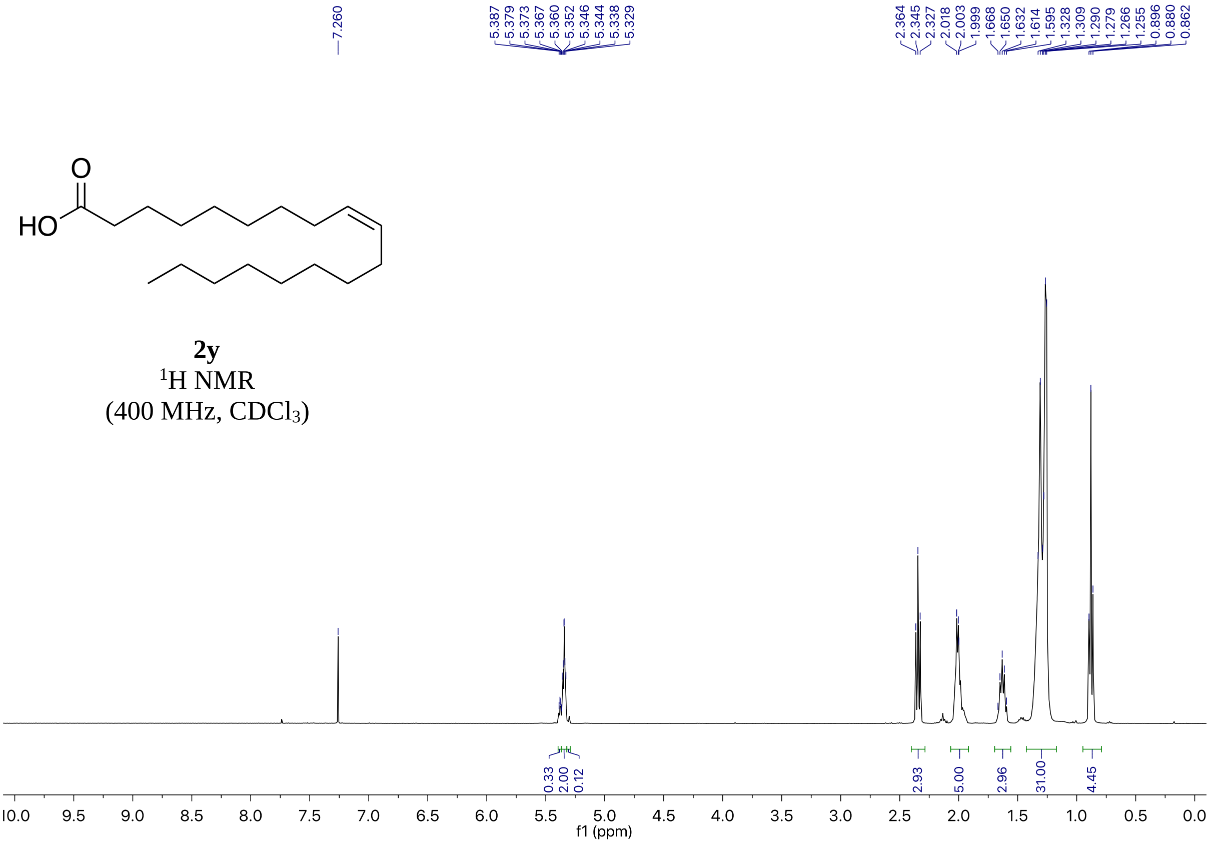

2y
^{1}H NMR
(400 MHz, $CDCl_3$)

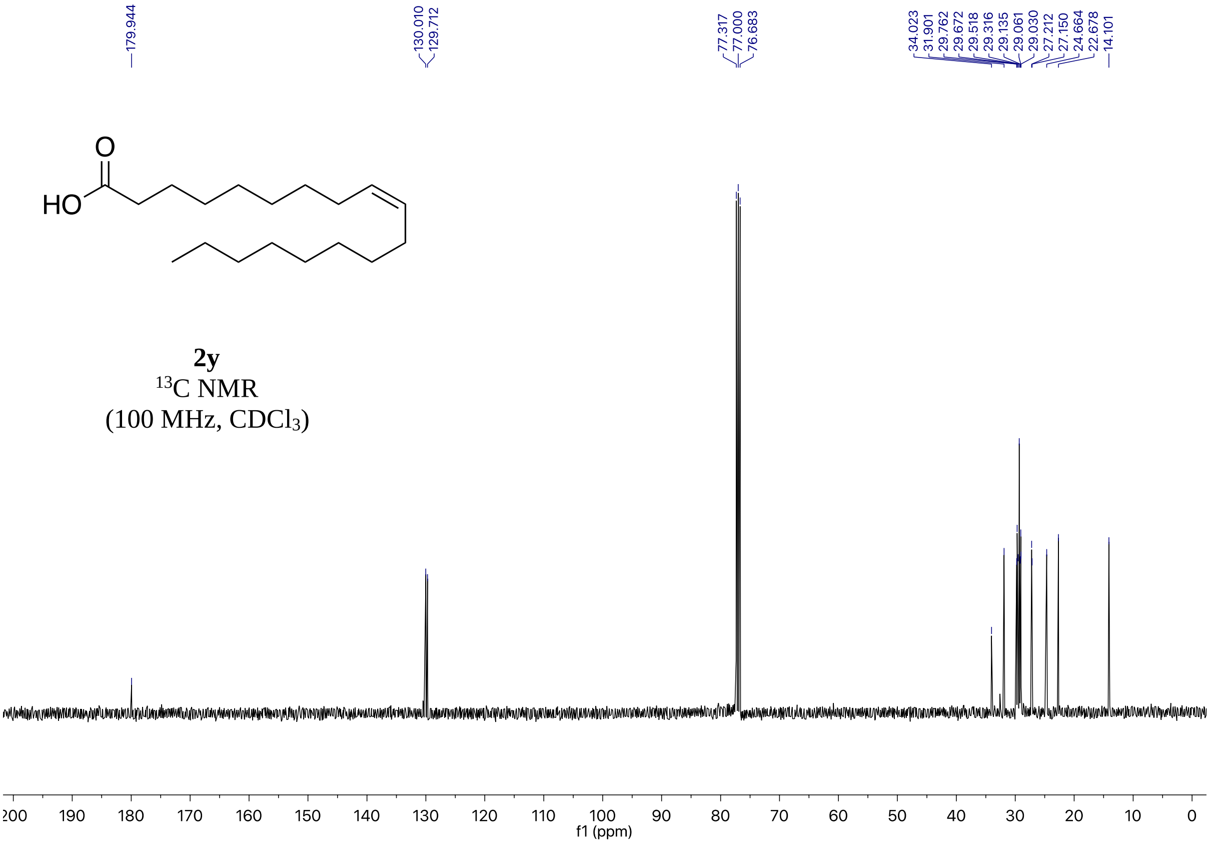

2y
^{13}C NMR
(100 MHz, $CDCl_3$)

Hex-5-enoic acid (2z)

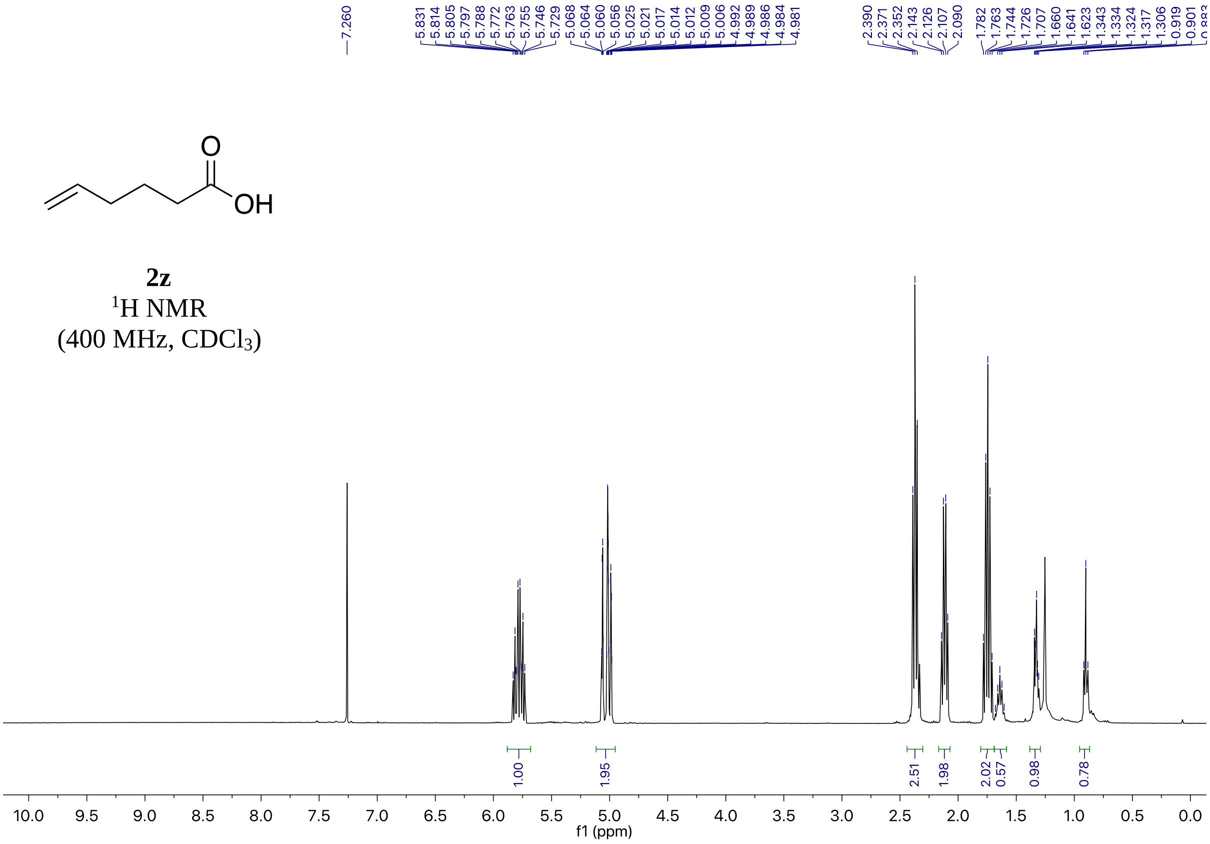

2z
^{1}H NMR
(400 MHz, $CDCl_3$)

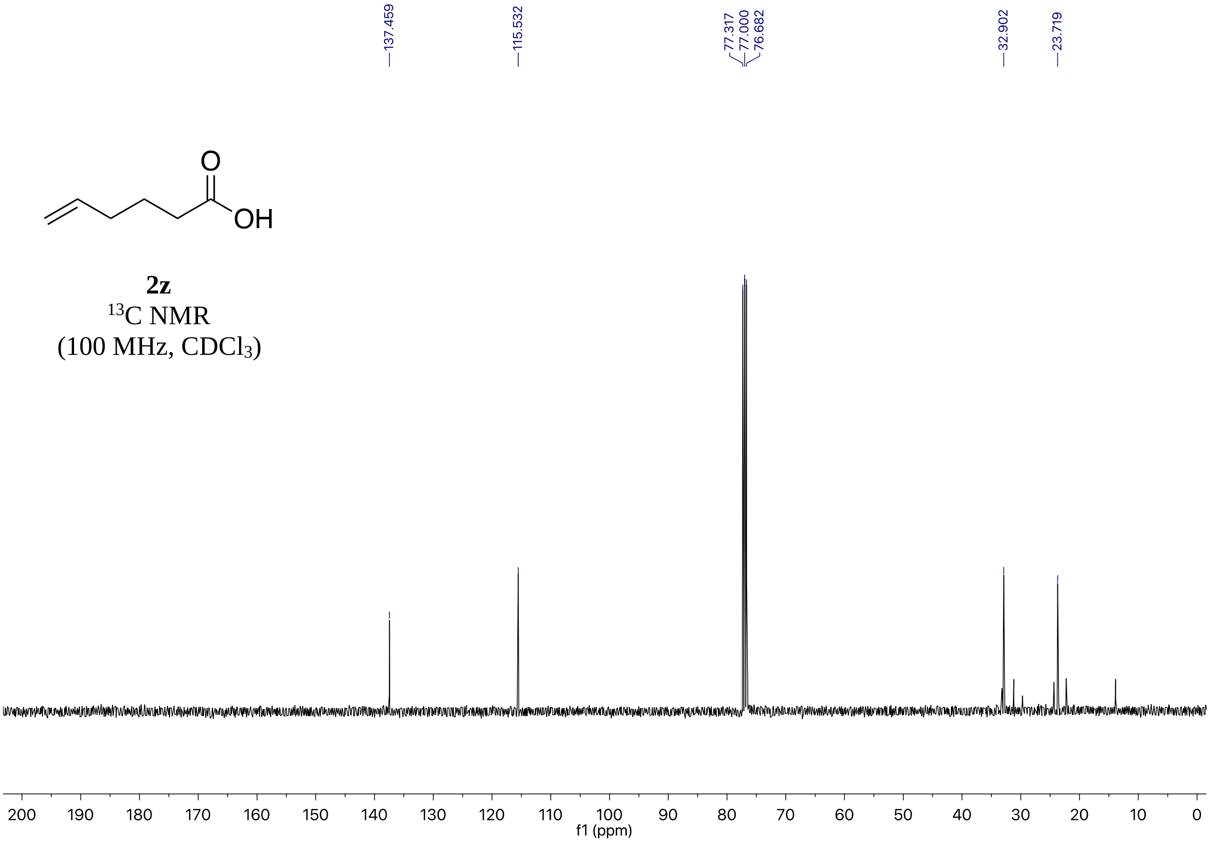

2z
^{13}C NMR
(100 MHz, $CDCl_3$)

1-Methoxy-4-vinylbenzene (2aa)

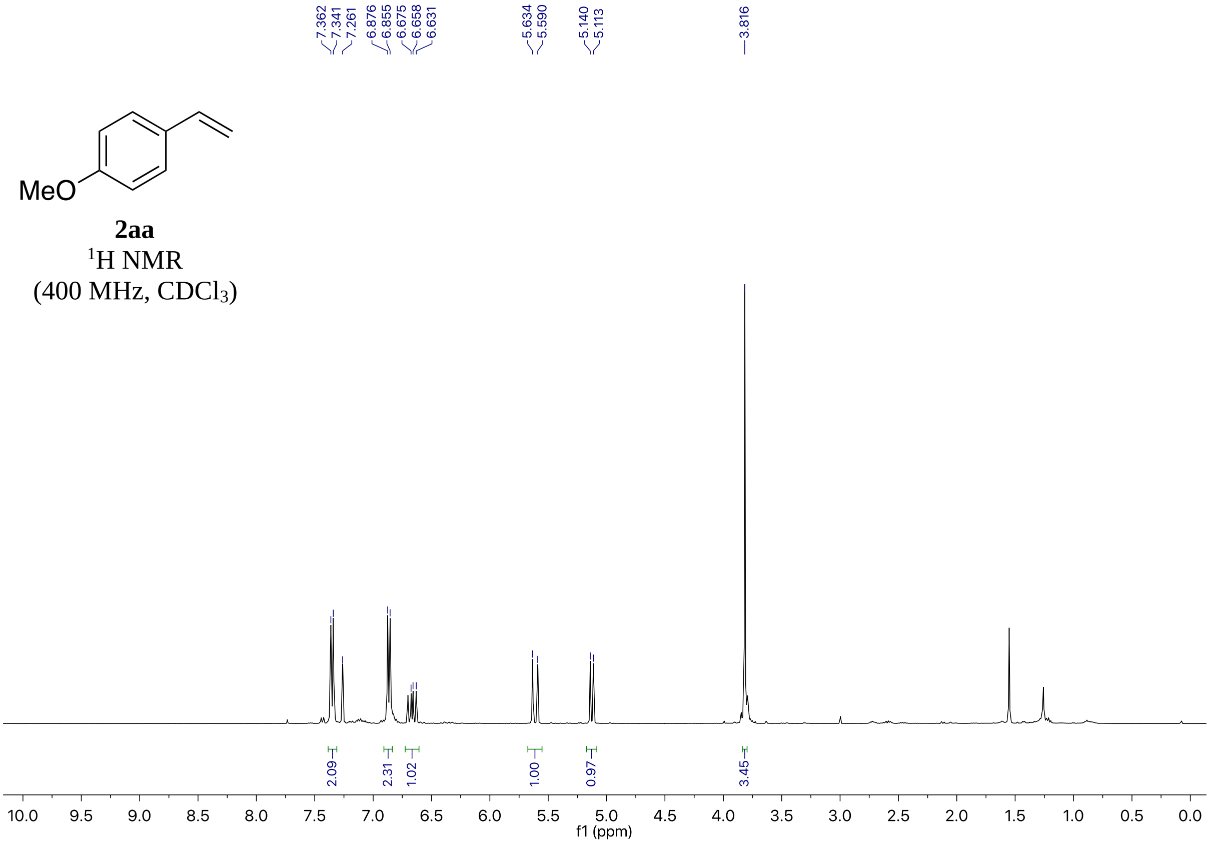

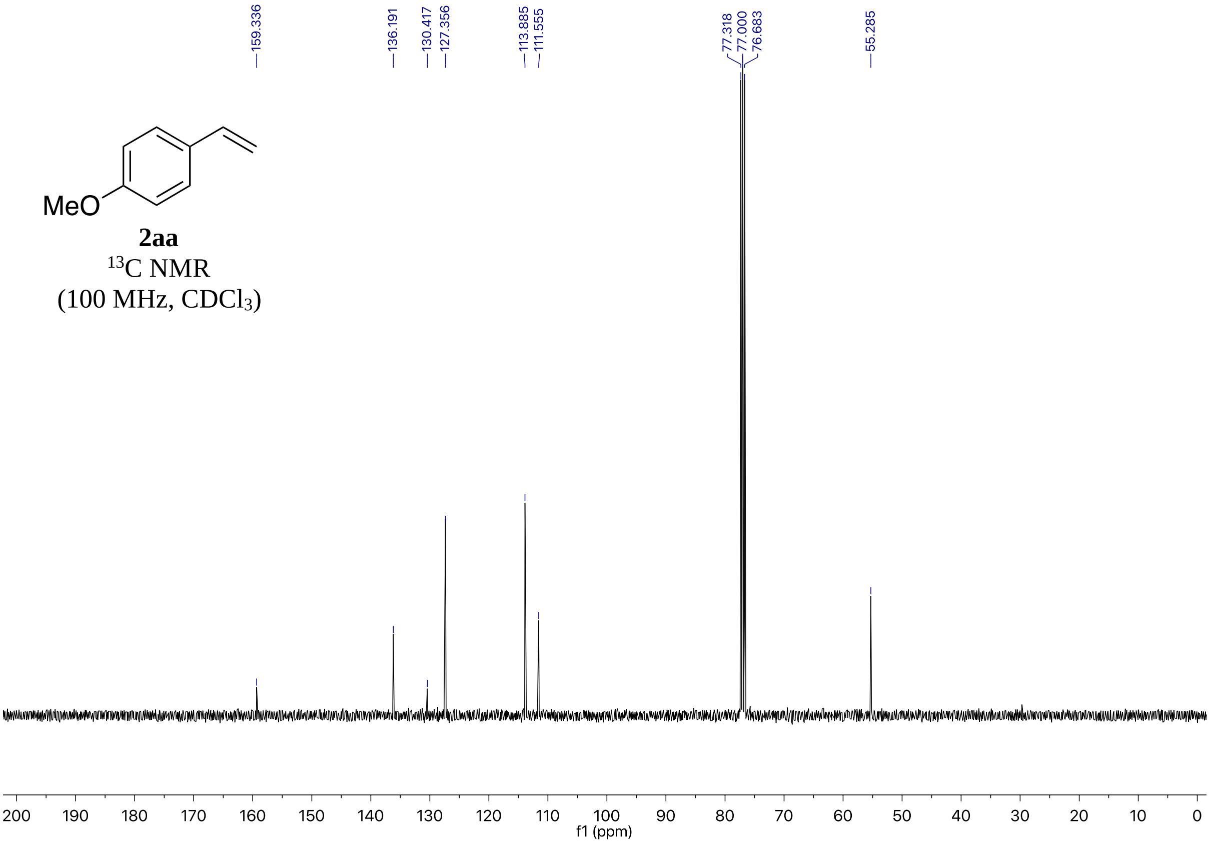

1-Bromo-4-vinylbenzene (2ab) and (4ab)

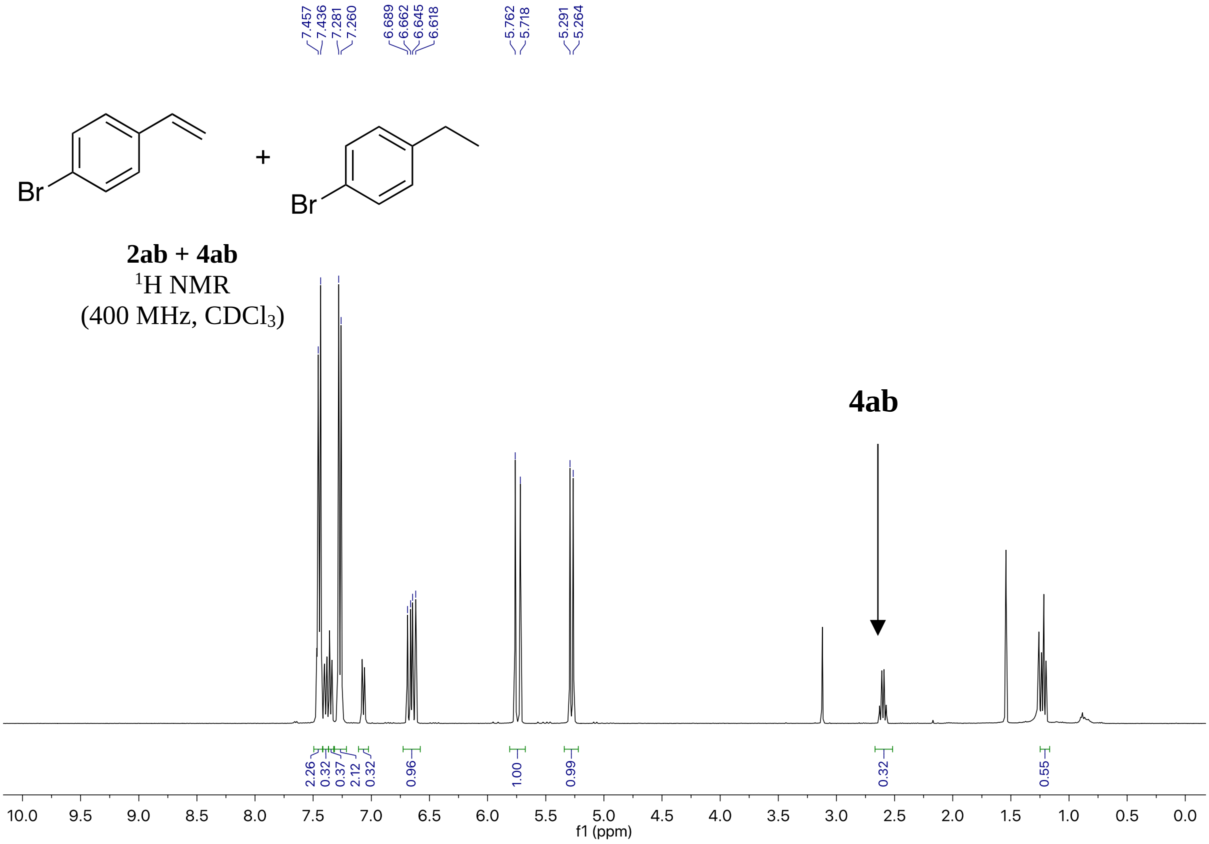

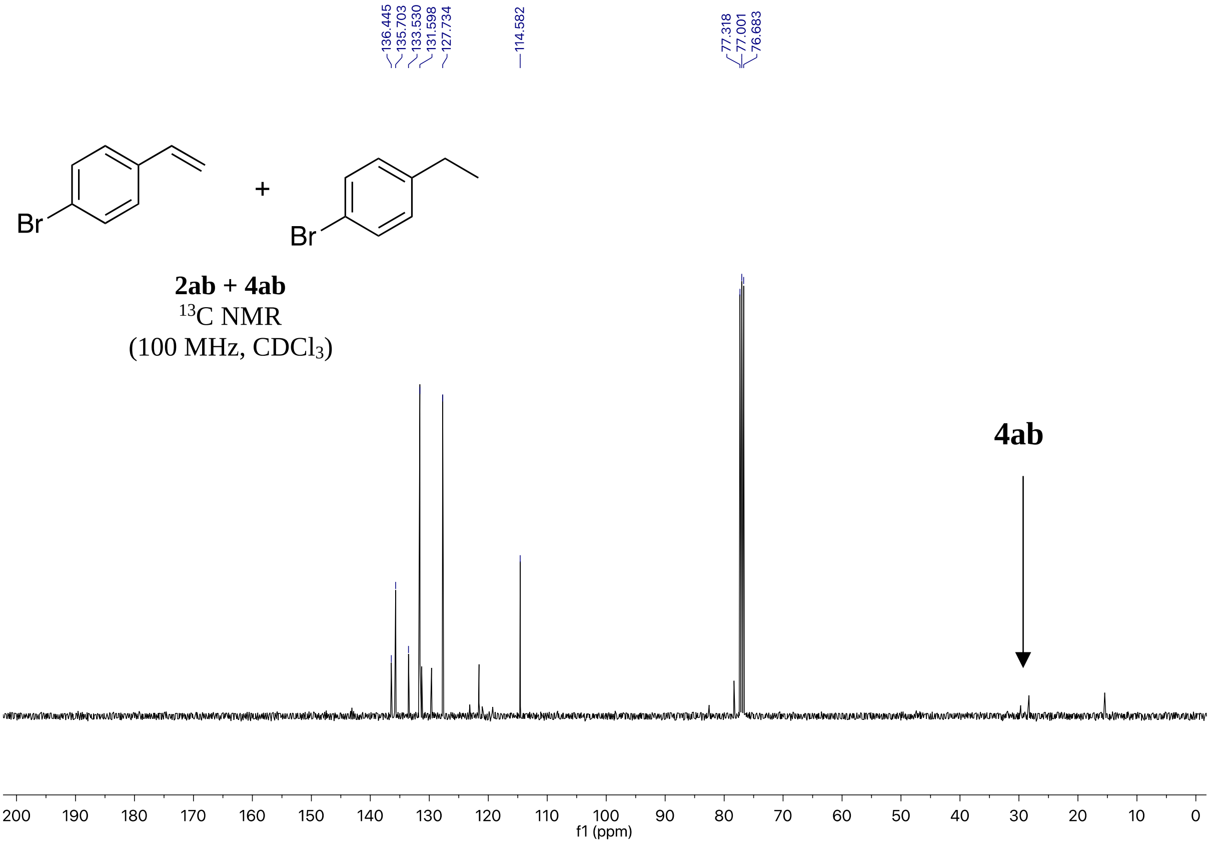

1-Iodo-4-vinylbenzene (2ac)

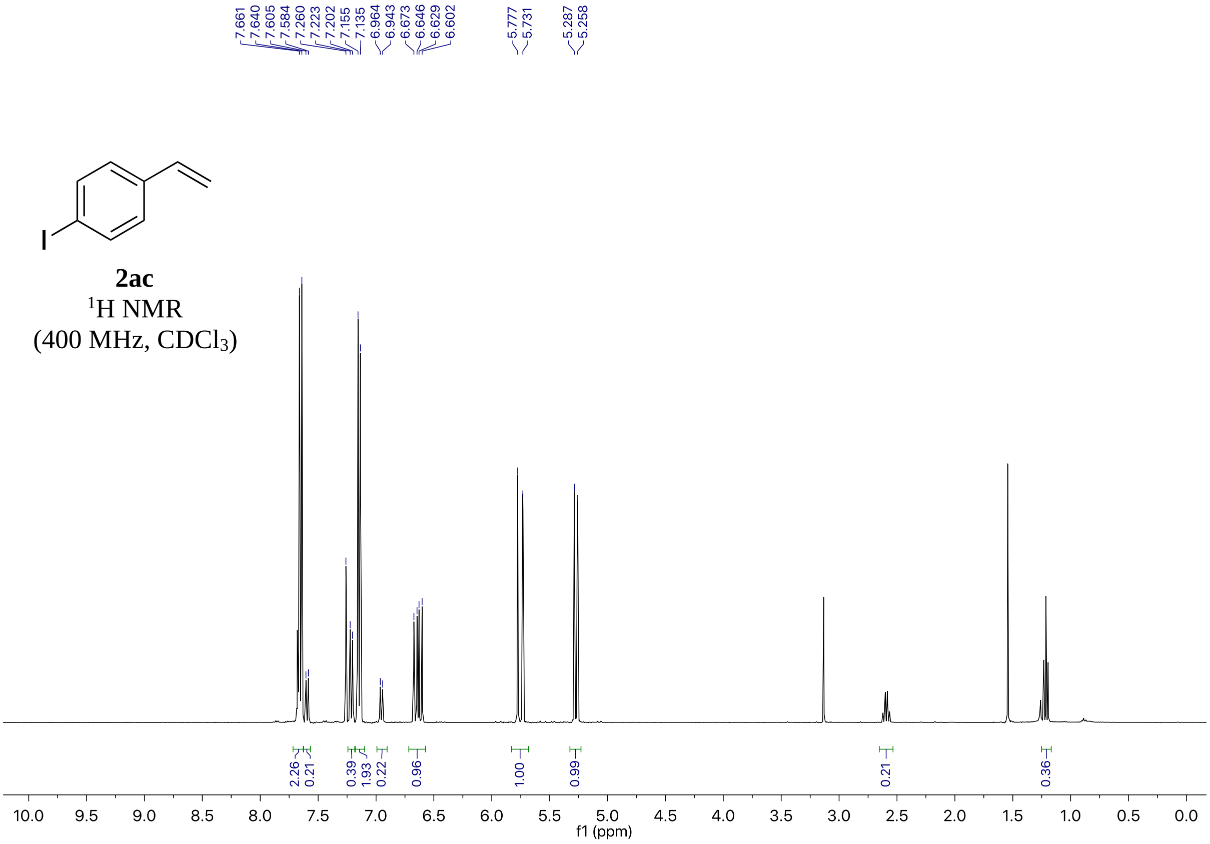

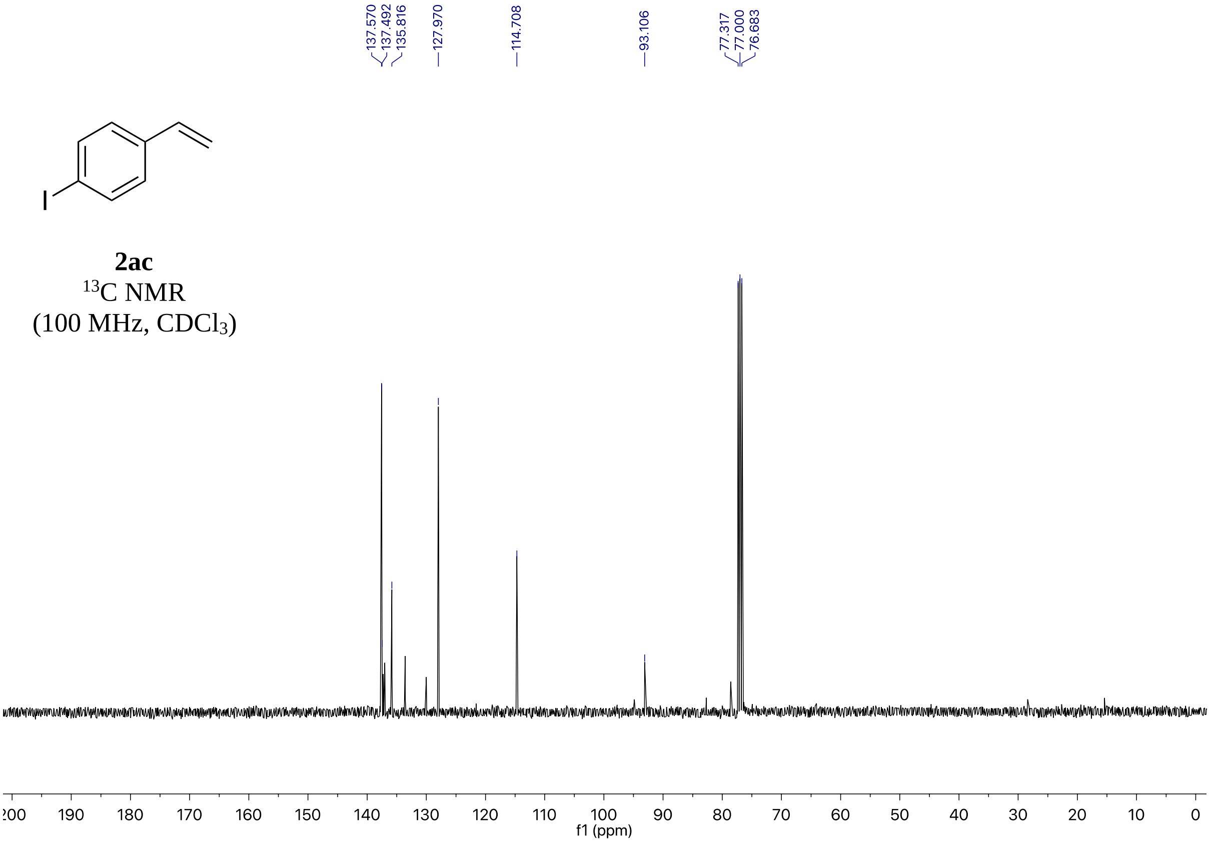

Vinyl stradiol (2ad) and (1ad)

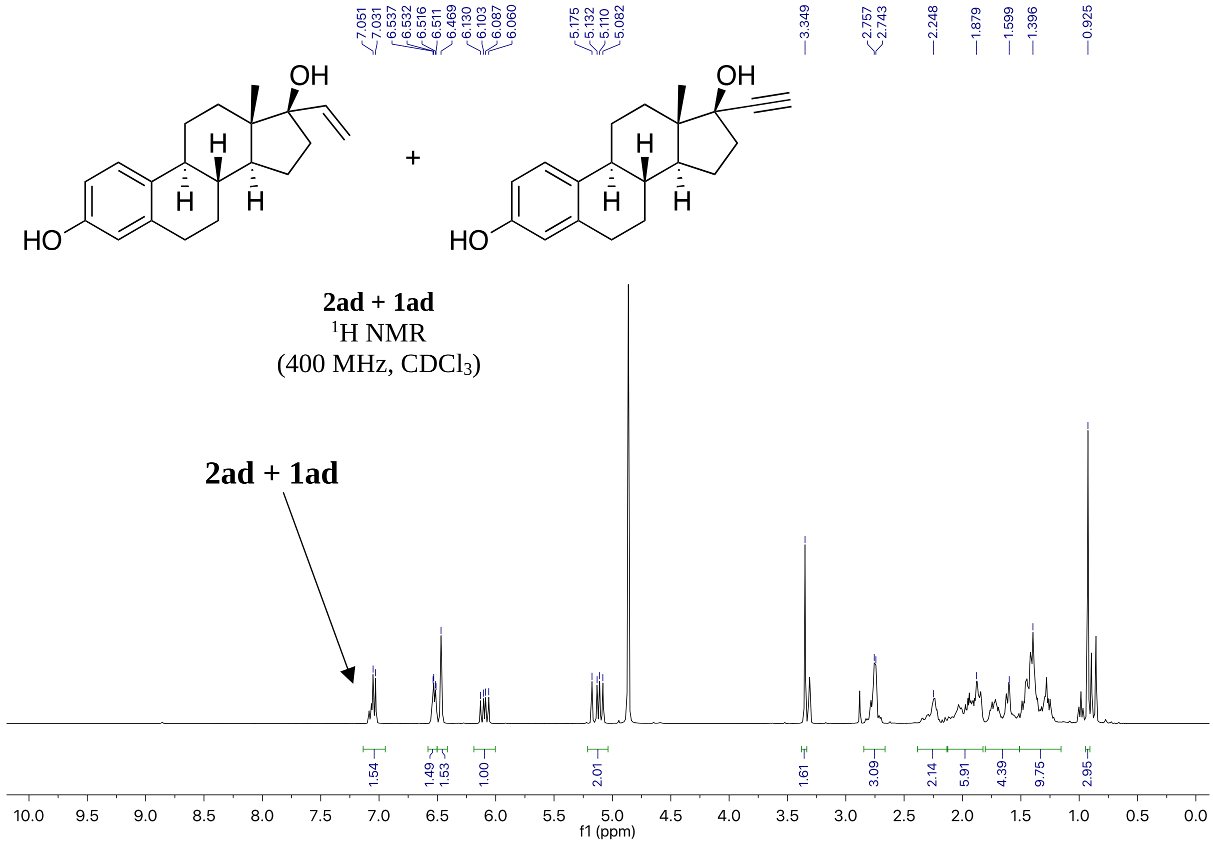

2ad + 1ad
^{1}H NMR
(400 MHz, $CDCl_3$)

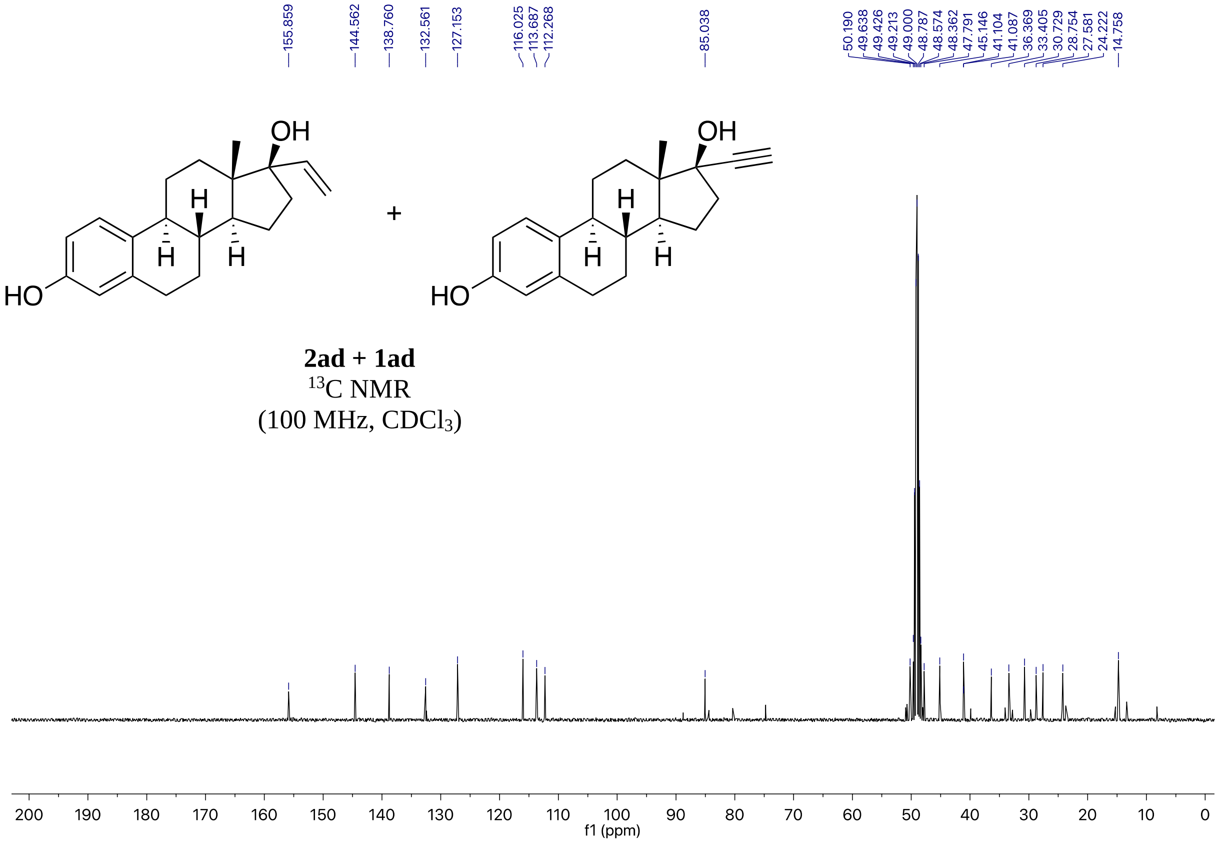

2ad + 1ad
^{13}C NMR
(100 MHz, $CDCl_3$)

NMR of compounds 2-[D]

(*Z*)-1-(2-Phenylvinyl-1,2-*d*2)-4-(trifluoromethoxy)benzene (2d-[D])

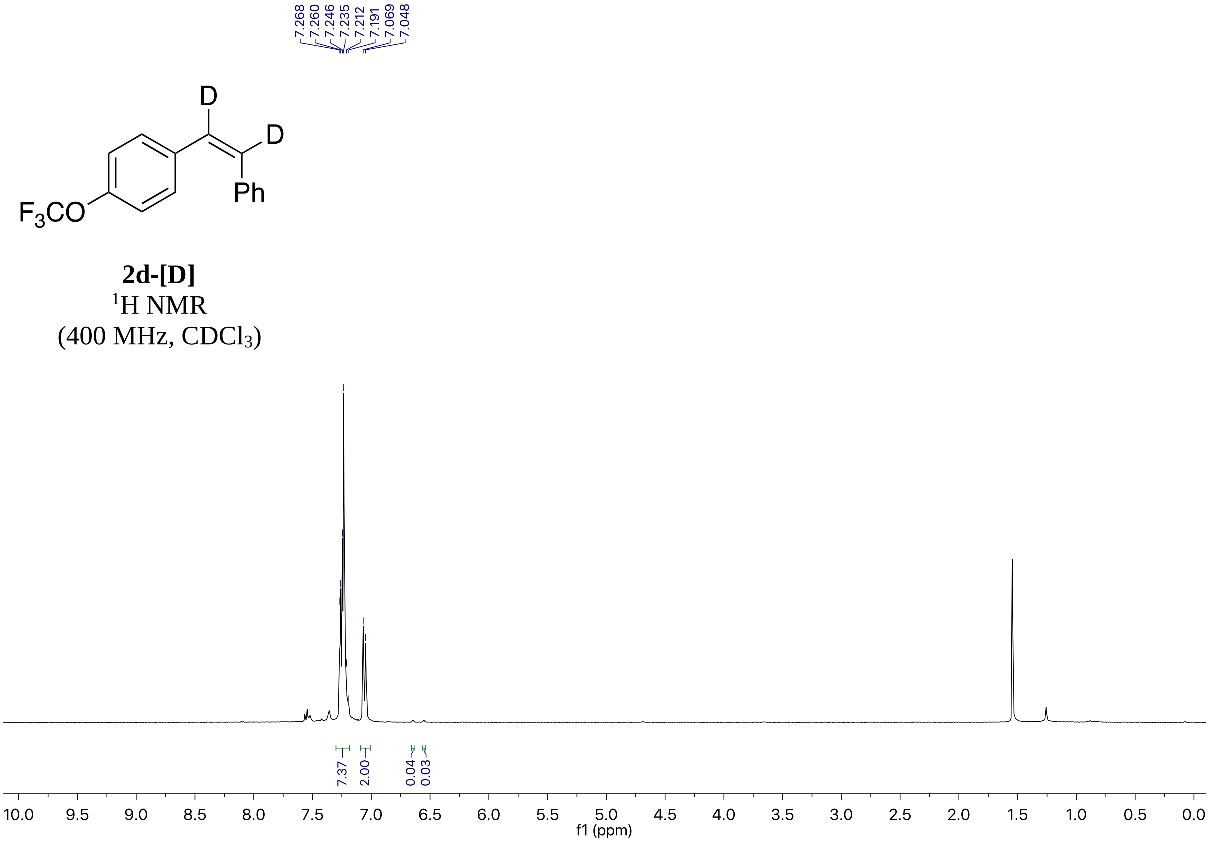

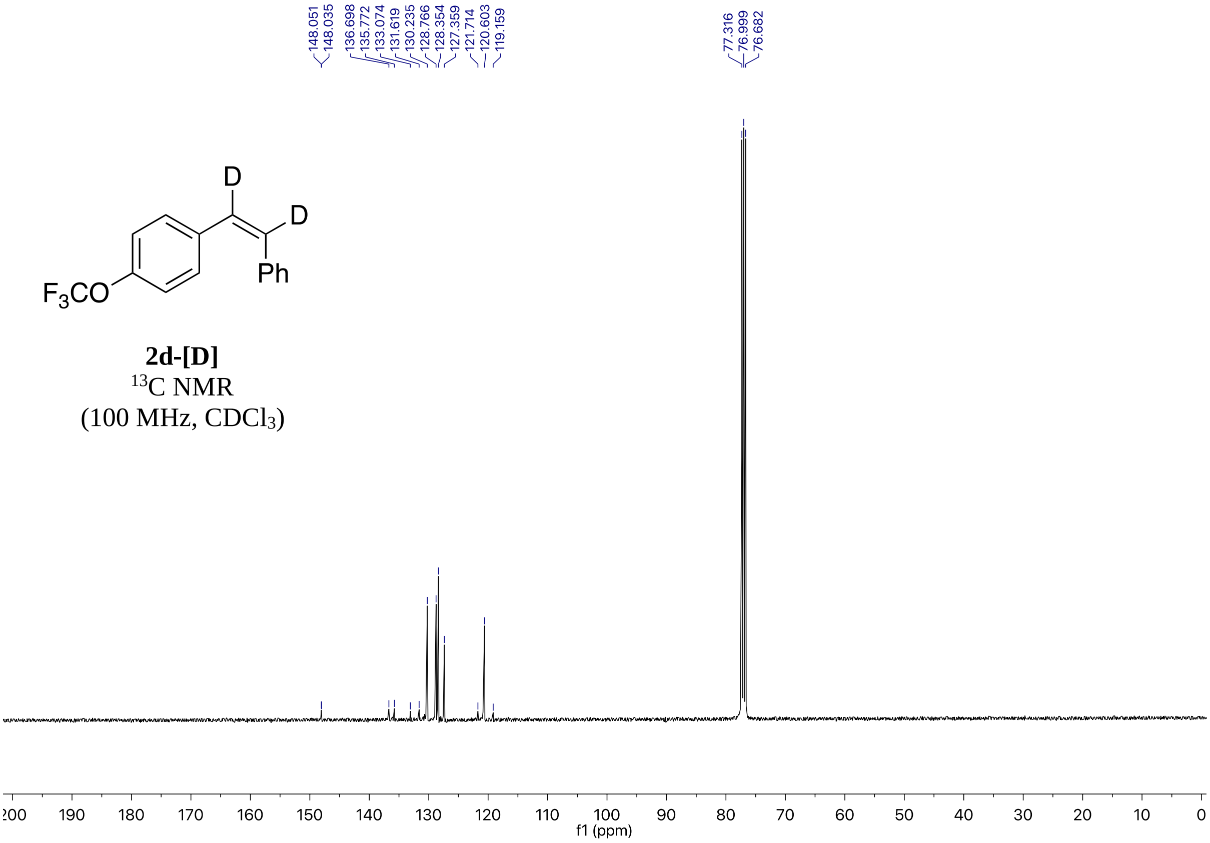

(*Z*)-1-Bromo-3-(2-phenylvinyl-1,2-*d*2)benzene (2j-[D])

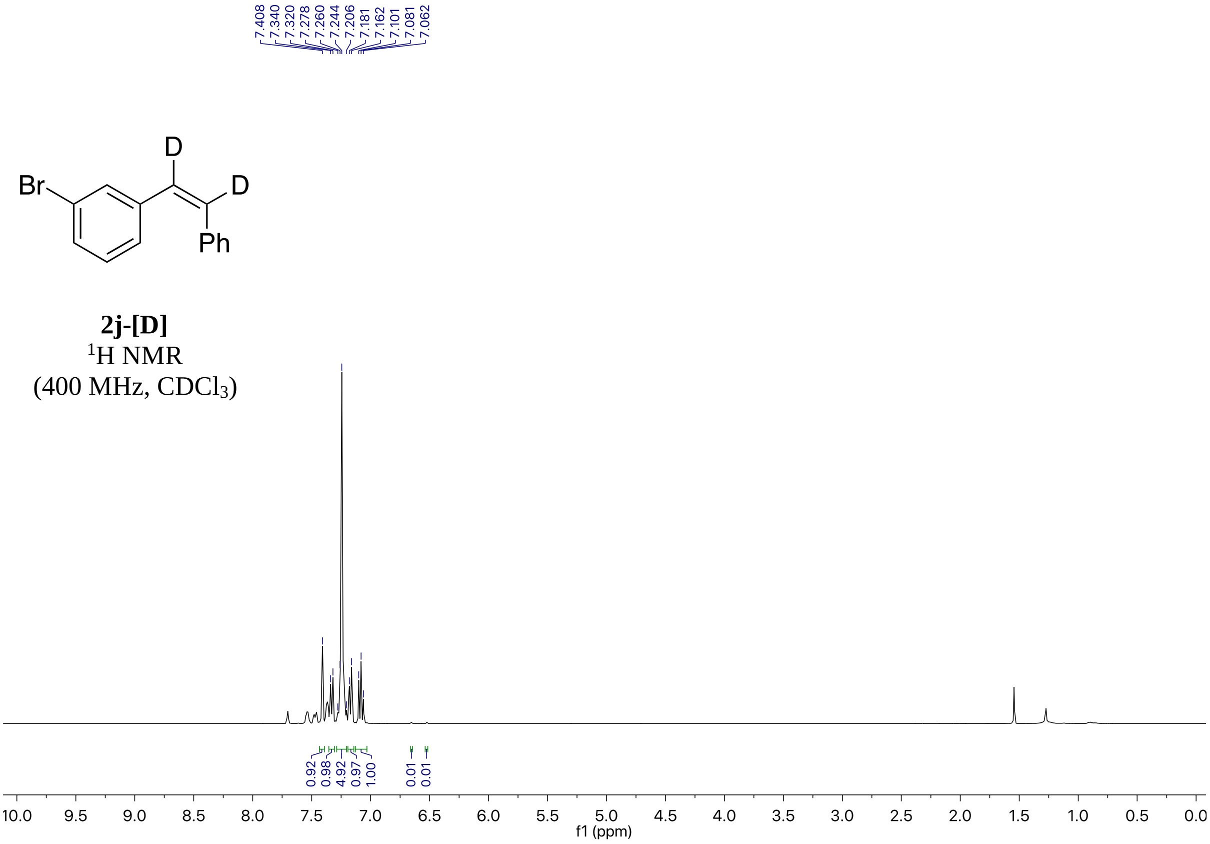

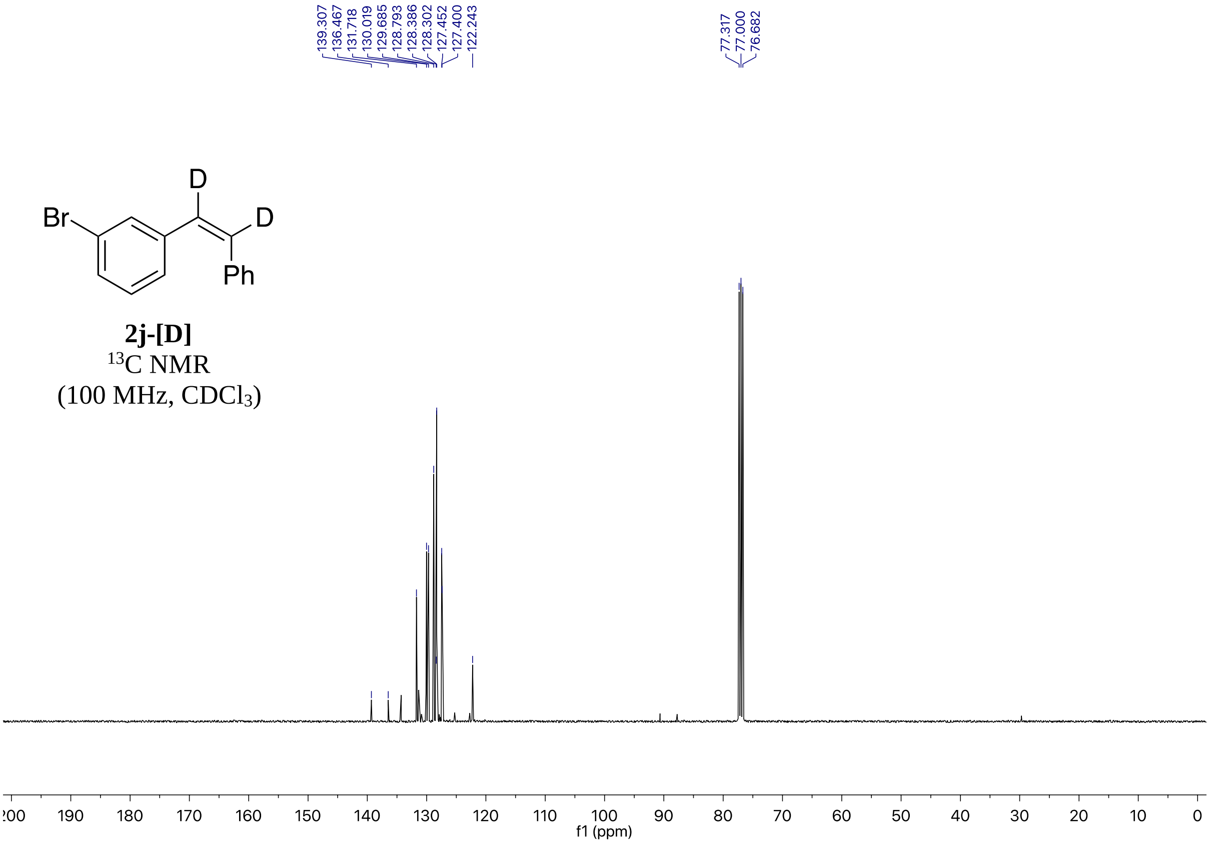

(*Z*)-1-Bromo-2-(2-phenylvinyl-1,2-*d*2)benzene (2l-[D])

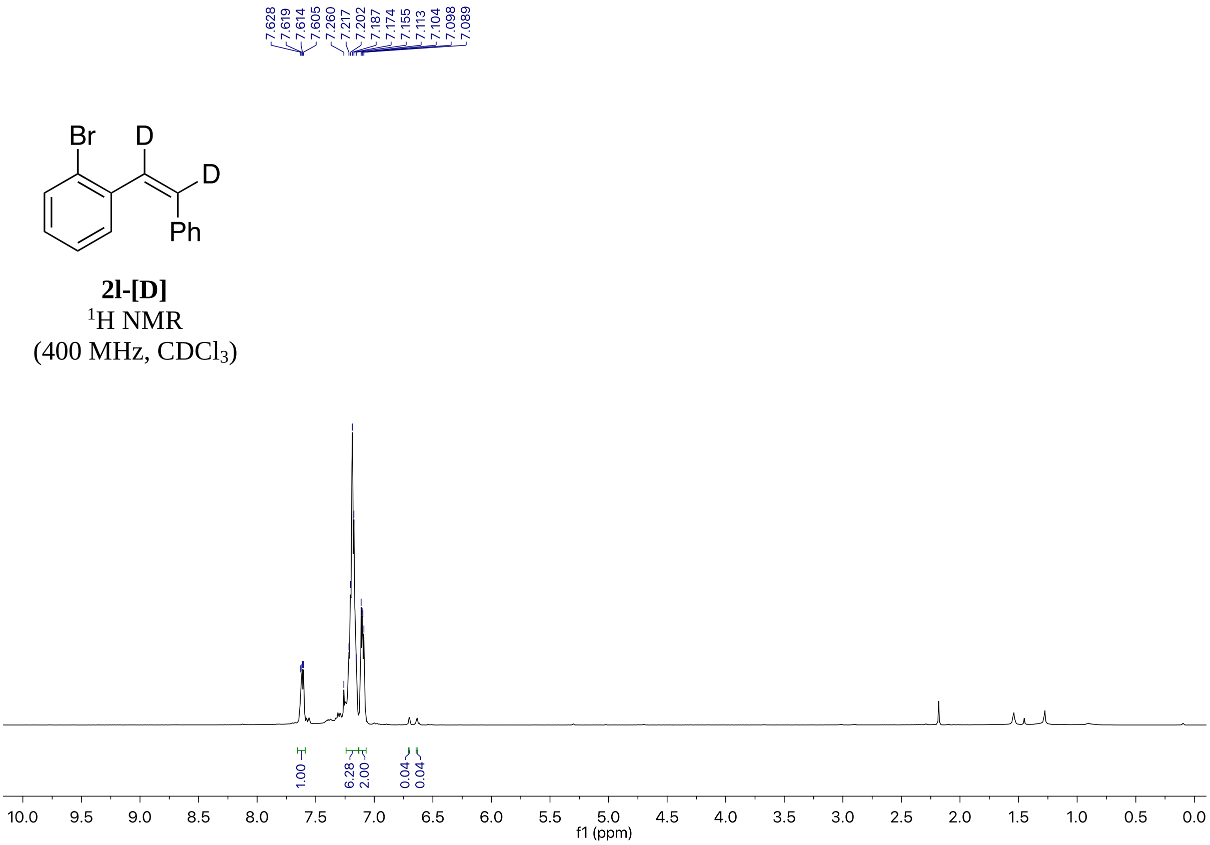

2l-[D]
^{1}H NMR
(400 MHz, $CDCl_3$)

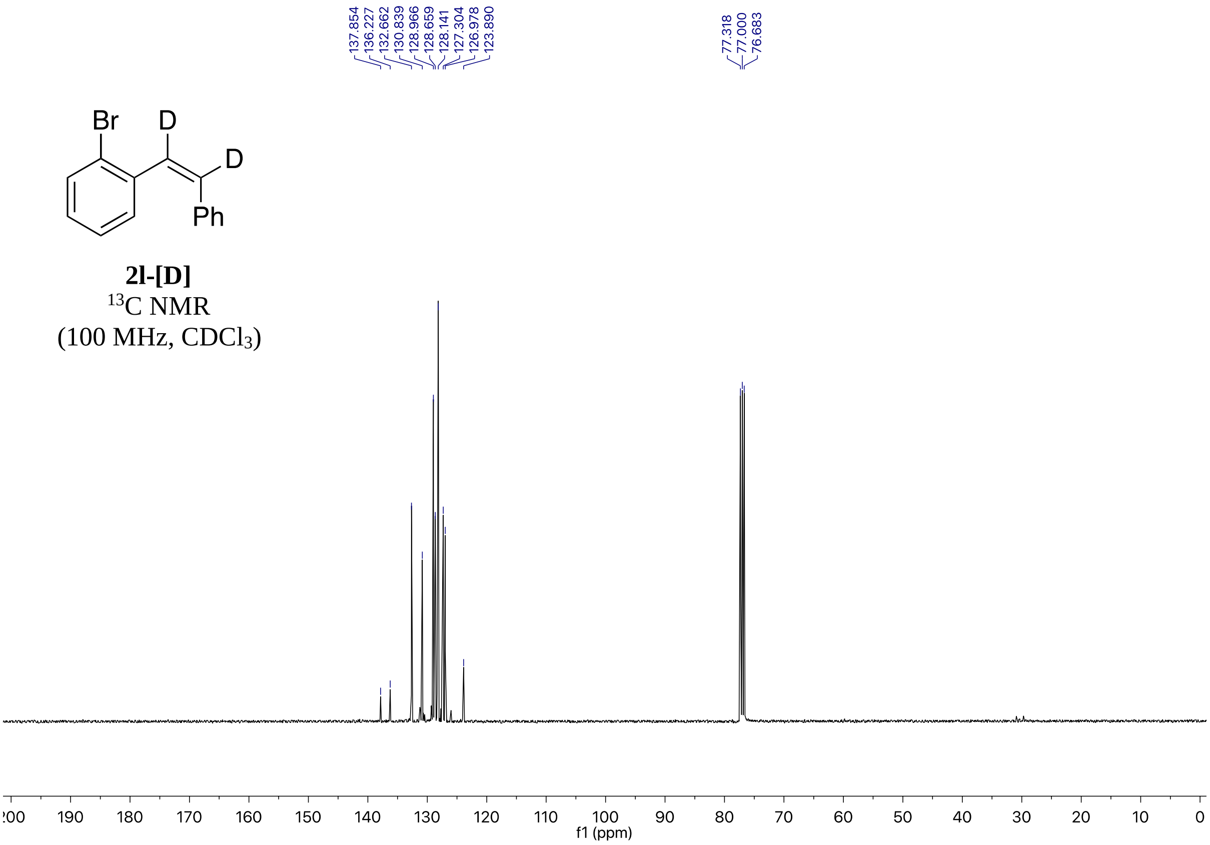

2l-[D]
^{13}C NMR
(100 MHz, $CDCl_3$)

(*Z*)-2,4-Dichloro-1-(2-phenylvinyl-1,2-*d*2)benzene (2n-[D])

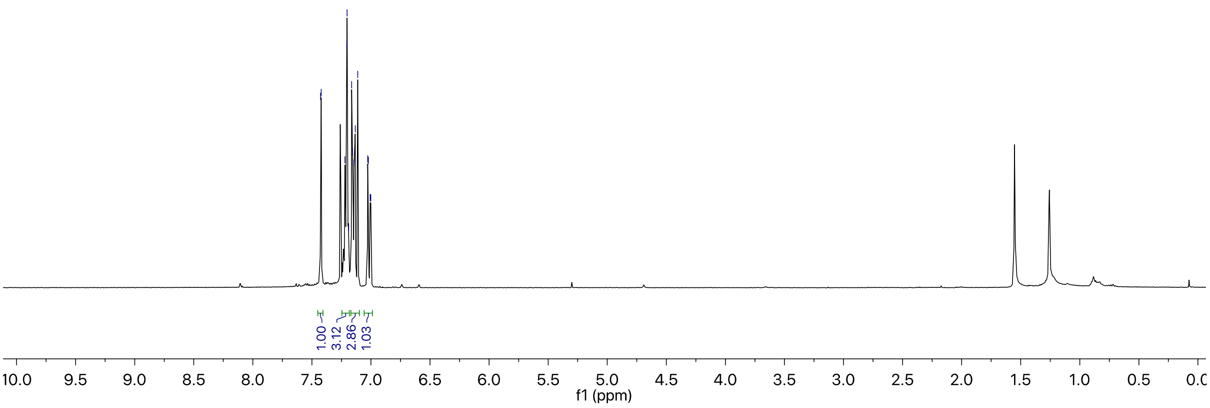

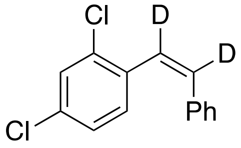

2n-[D]
^{1}H NMR
(400 MHz, $CDCl_3$)

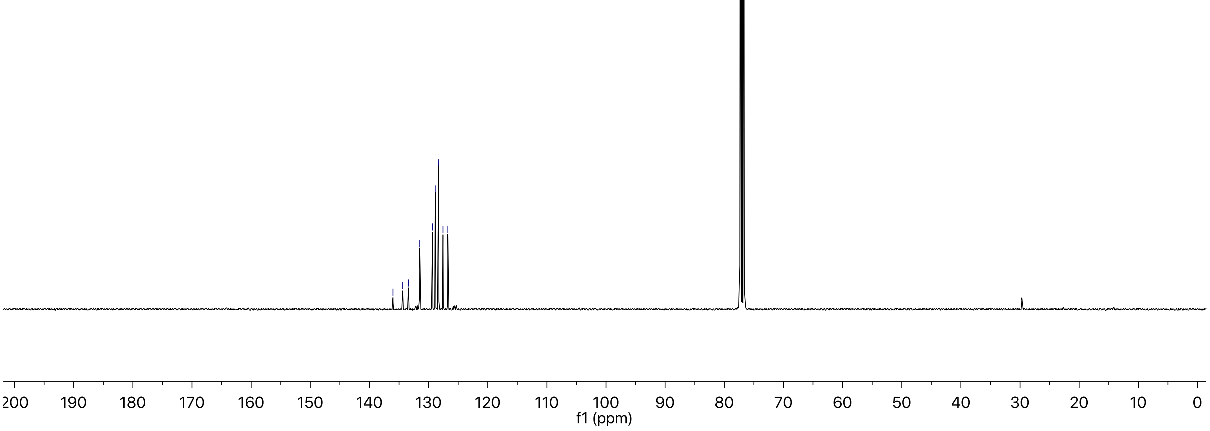

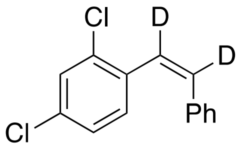

2n-[D]
^{13}C NMR
(100 MHz, $CDCl_3$)

(*Z*)-3-(2-(Thiophen-3-yl)vinyl-1,2-*d*2)pyridine (2p-[D])

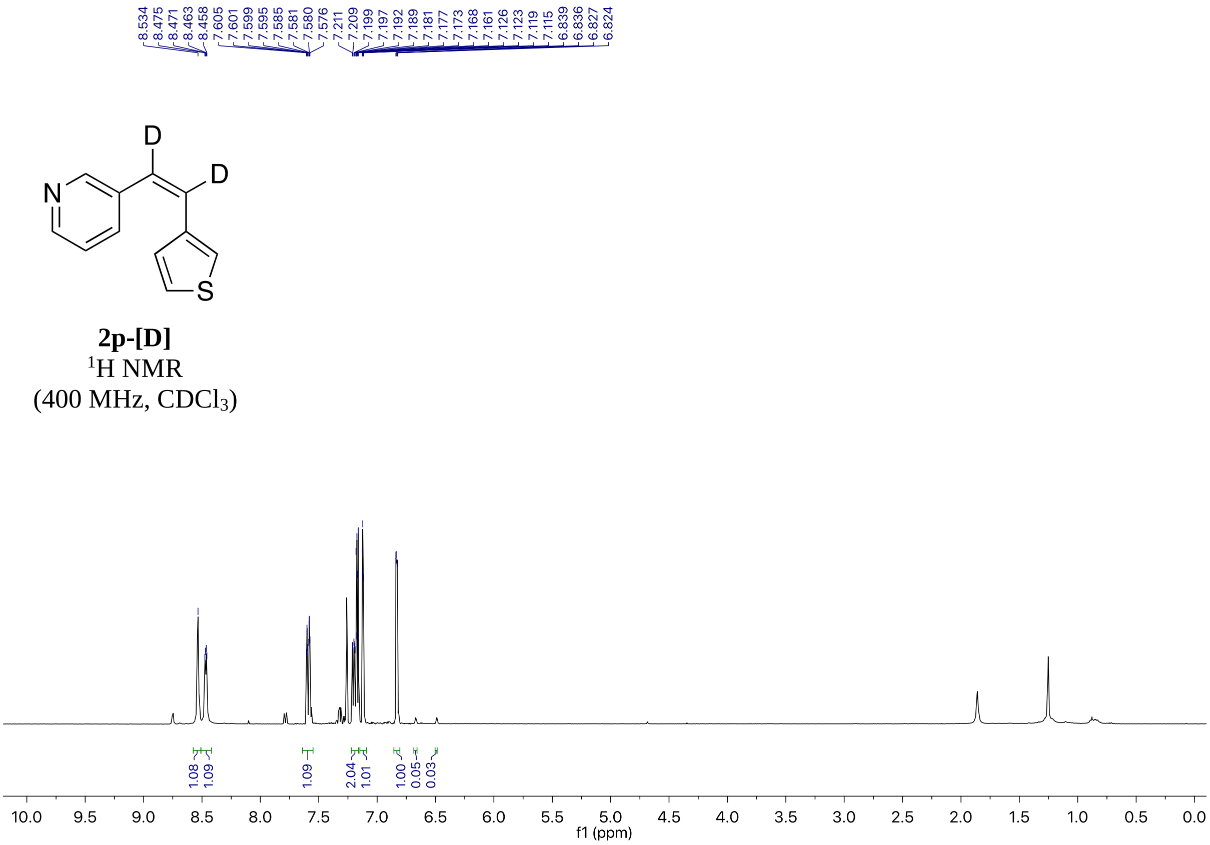

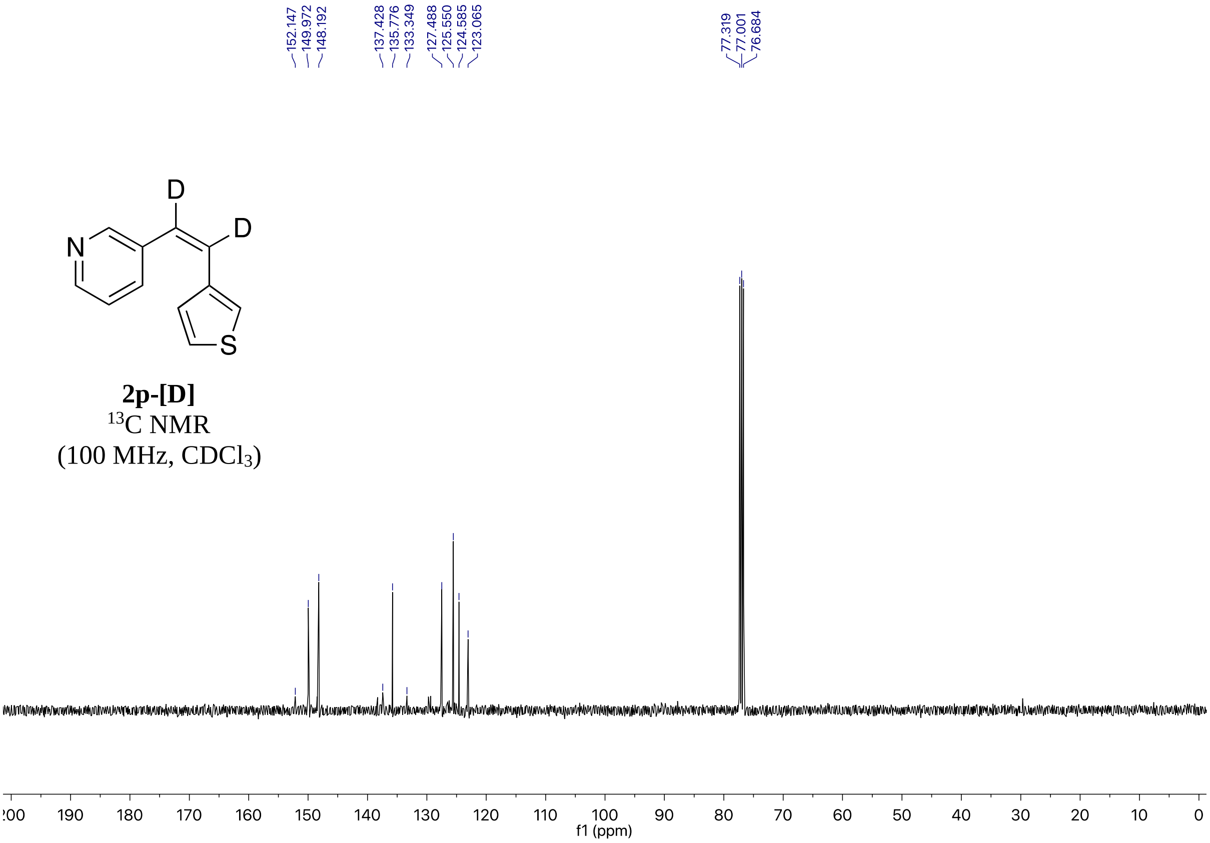

Methyl (*Z*)-3-phenylacrylate-*d*2 (2s-[D])

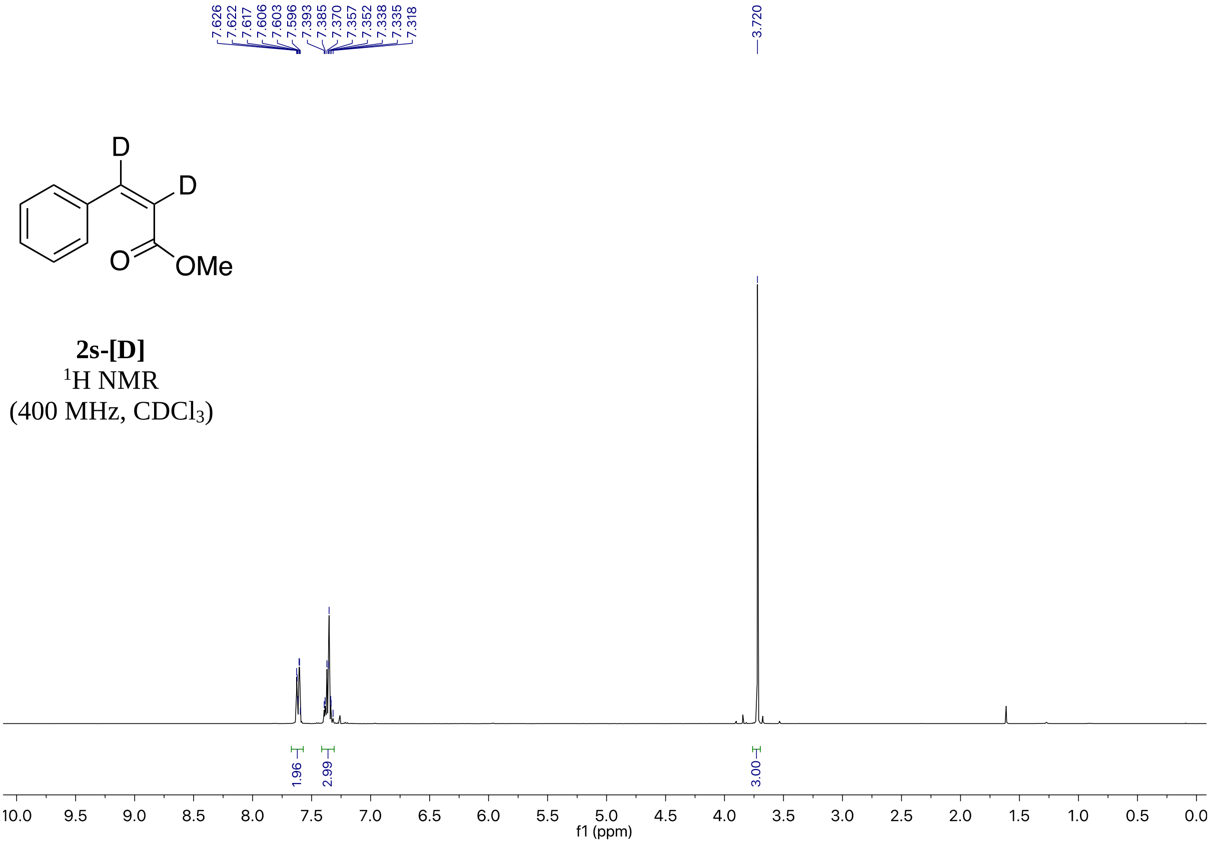

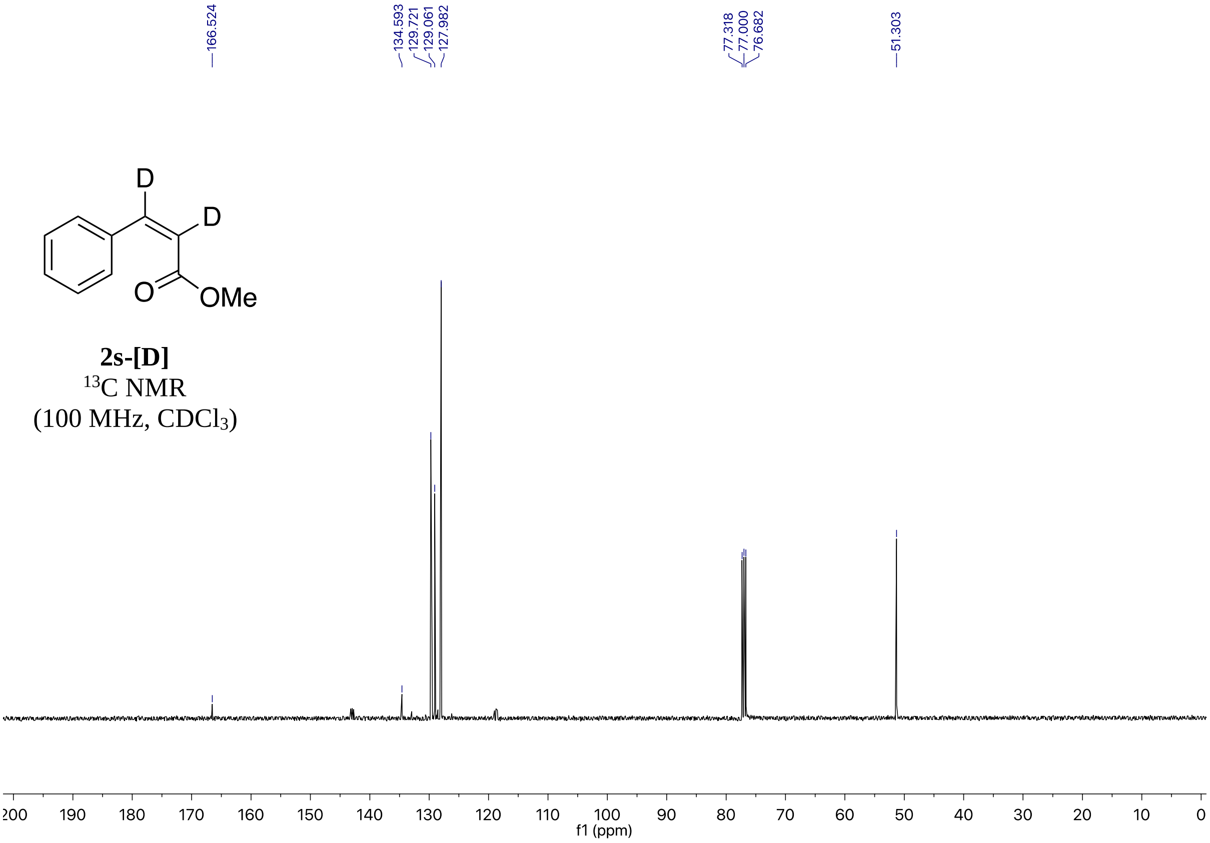

Methyl (*Z*)-3-(4-iodophenyl)acrylate-*d*2 (2t-[D])

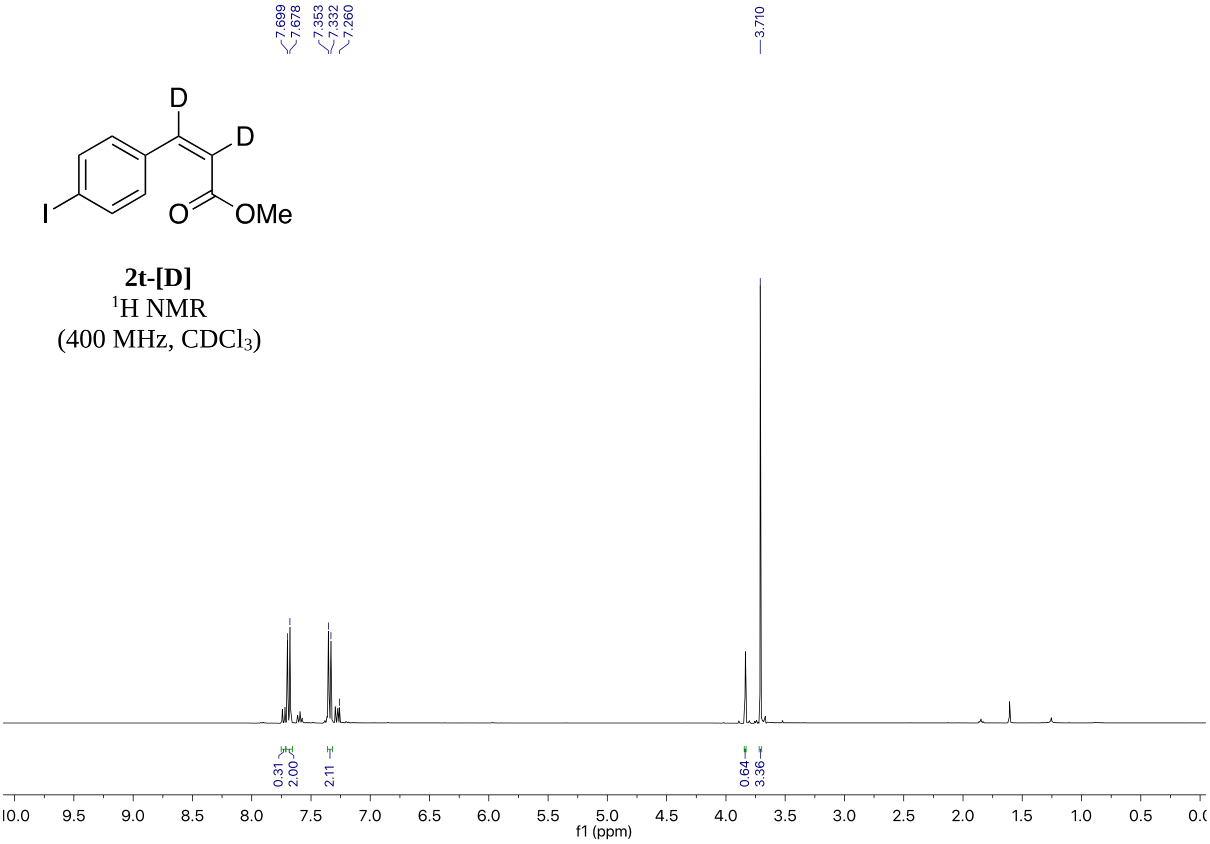

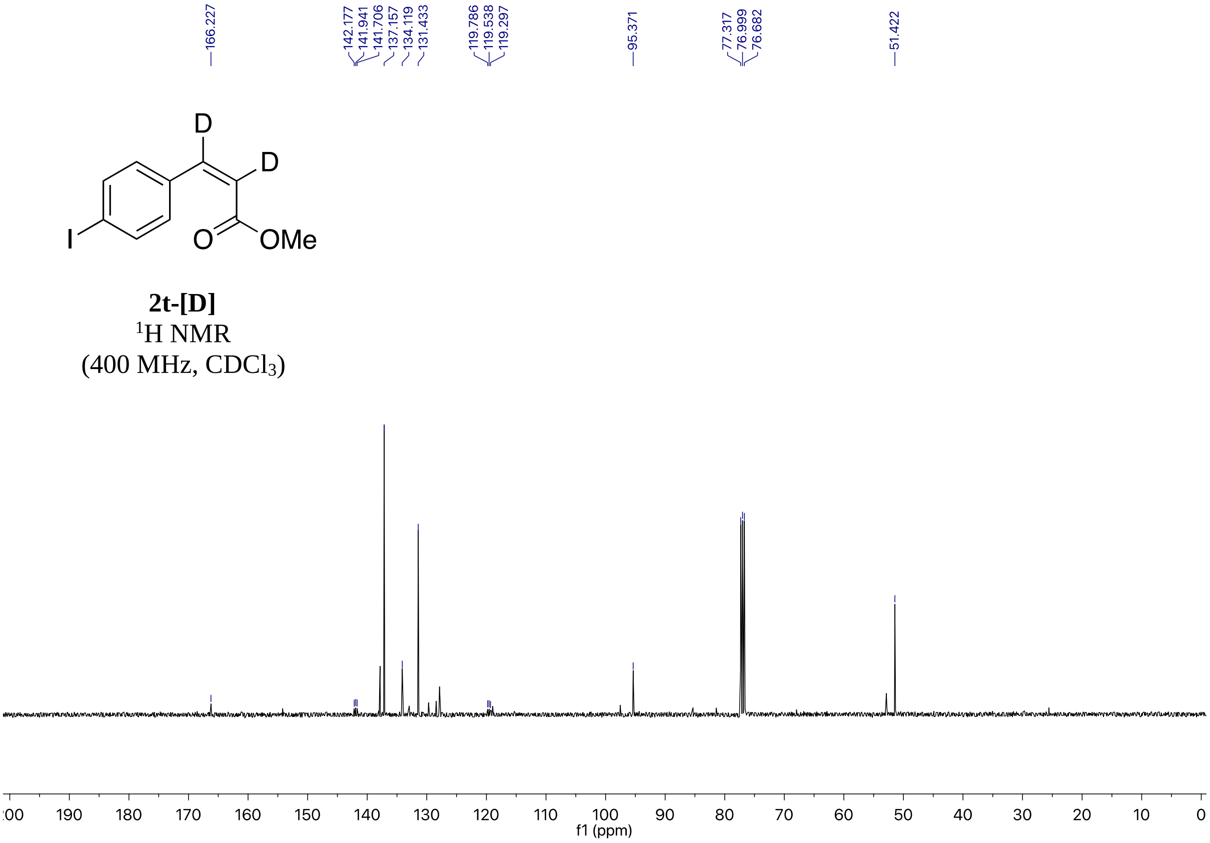

Oleic acid *d*2 (2y-[D])

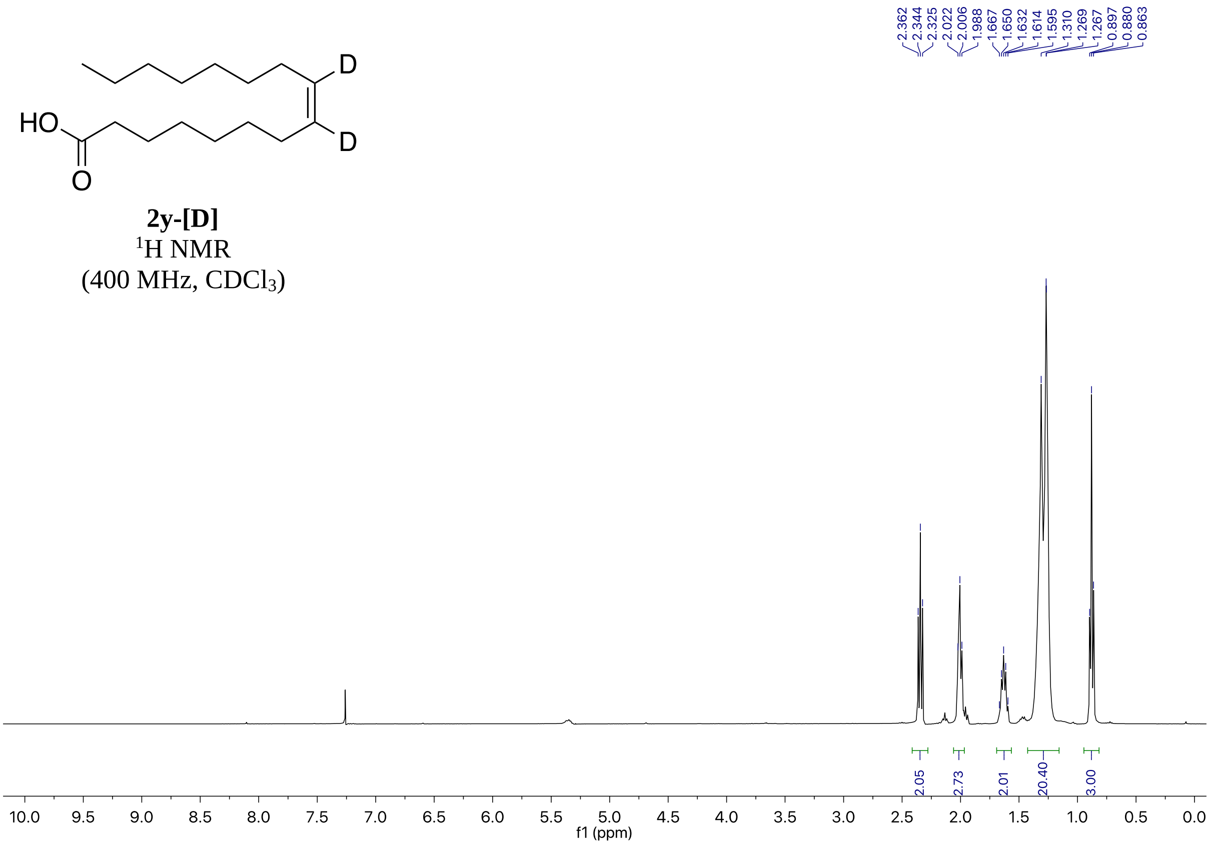

2y-[D]
^{1}H NMR
(400 MHz, $CDCl_3$)

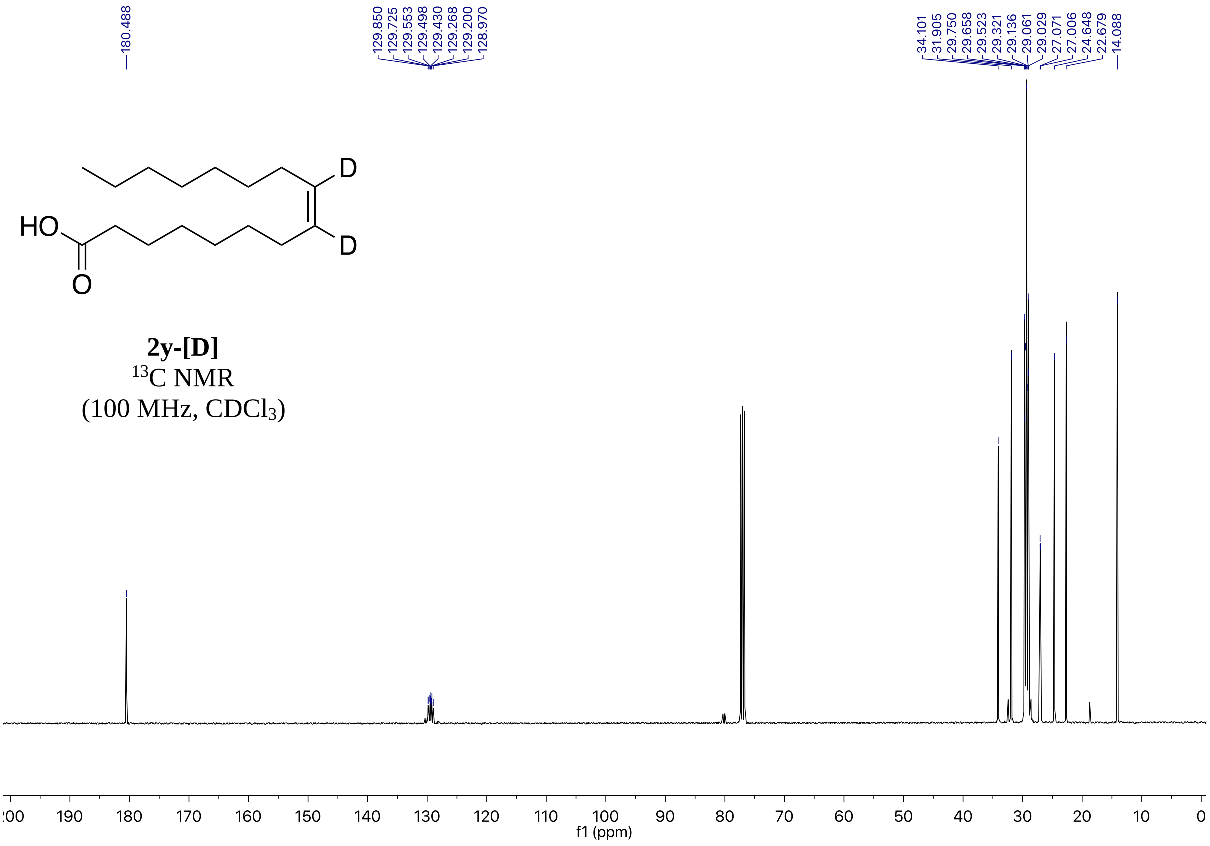

2y-[D]
^{13}C NMR
(100 MHz, $CDCl_3$)

(*E*)-Hex-5-enoic-5,6-*d*2 acid (2z-[D])

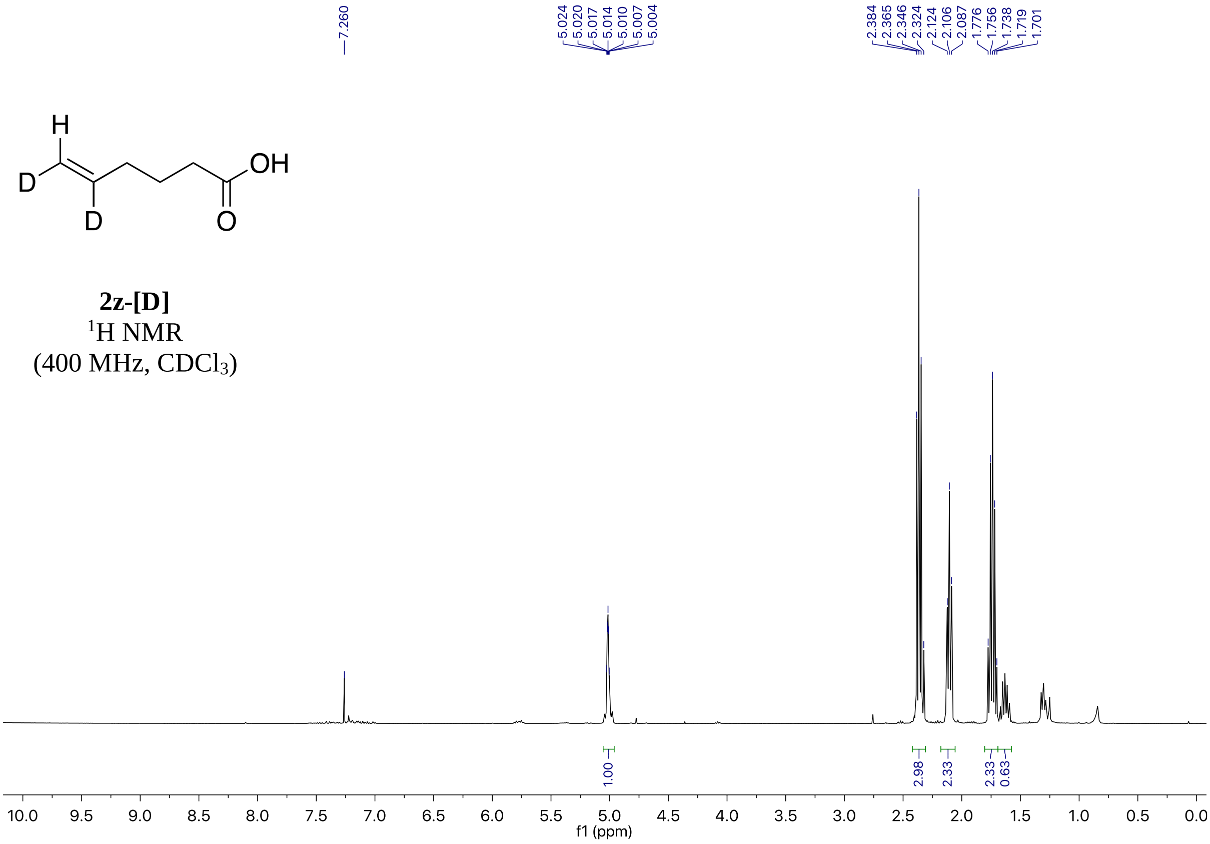

2z-[D]
^{1}H NMR
(400 MHz, $CDCl_3$)

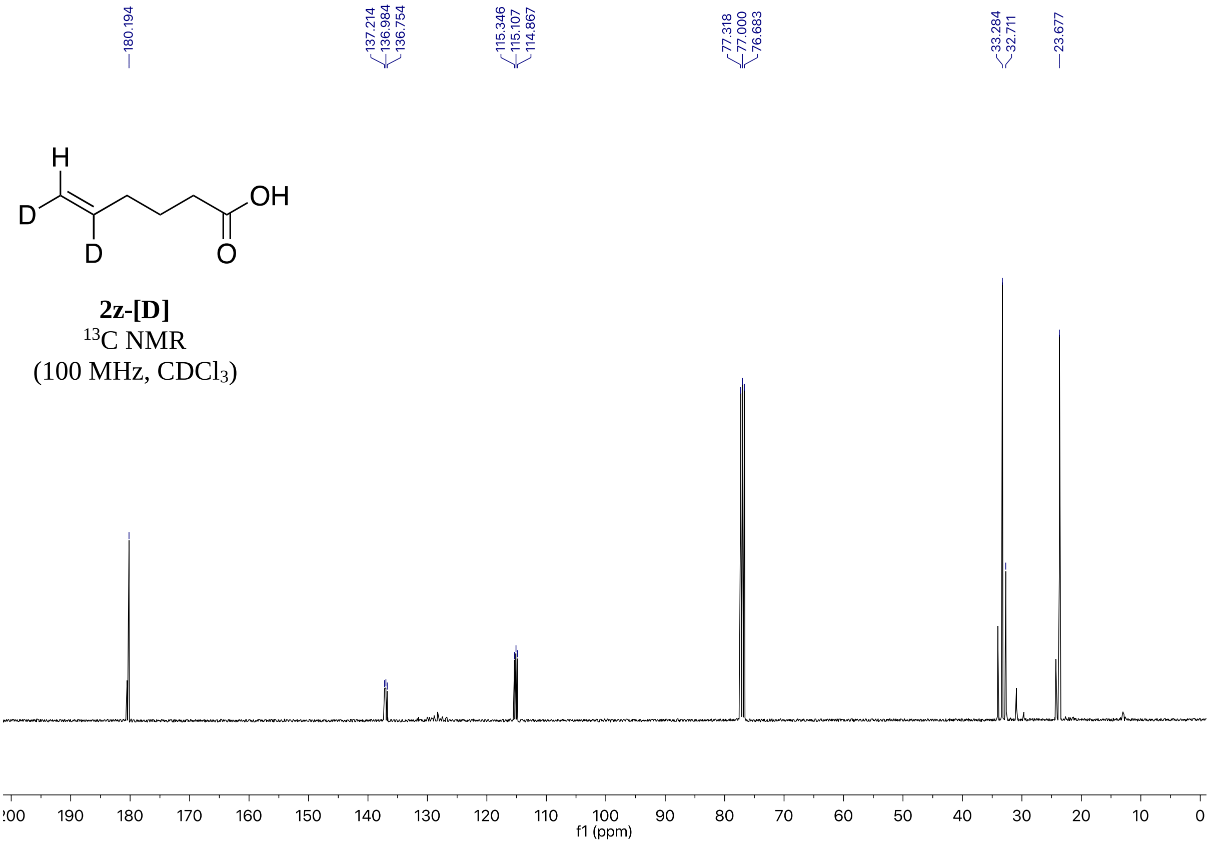

2z-[D]
^{13}C NMR
(100 MHz, $CDCl_3$)

7. References

(1) Hamasaka, G.; Roy, D.; Tazawa, A.; Uozumi, Y. Arylation of Terminal Alkynes by Aryl Iodides Catalyzed by a Parts-per-Million Loading of Palladium Acetate. *ACS Catal.* **2019**, *9* (12), 11640–11646. https://doi.org/10.1021/acscatal.9b04593.

(2) Abderrezak, M. K.; Kabouche, Z.; Bruneau, C.; Fischmeister, C. Ene-yne Cross-metathesis for the Preparation of 2,3-diaryl-1,3-dienes. *Catalysts* **2017**, *7* (12). https://doi.org/10.3390/catal7120365.

(3) Zhou, M. B.; Wei, W. T.; Xie, Y. X.; Lei, Y.; Li, J. H. Palladium-Catalyzed Cross-Coupling of Electron-Poor Terminal Alkynes with Arylboronic Acids under Ligand-Free and Aerobic Conditions. *J. Org. Chem.* **2010**, *75* (16), 5635–5642. https://doi.org/10.1021/jo101063p.

(4) Schabel, T.; Belger, C.; Plietker, B. A Mild Chemoselective Ru-Catalyzed Reduction of Alkynes, Ketones, and Nitro Compounds. *Org. Lett.* **2013**, *15* (11), 2858–2861. https://doi.org/10.1021/ol401185t.

(5) Fu, S.; Chen, N. Y.; Liu, X.; Shao, Z.; Luo, S. P.; Liu, Q. Ligand-Controlled Cobalt-Catalyzed Transfer Hydrogenation of Alkynes: Stereodivergent Synthesis of Z- and E-Alkenes. *J. Am. Chem. Soc.* **2016**, *138* (27), 8588–8594. https://doi.org/10.1021/jacs.6b04271.

(6) Gregori, B. J.; Nowakowski, M.; Schoch, A.; Pöllath, S.; Zweck, J.; Bauer, M.; Jacobi von Wangelin, A. Stereoselective Chromium-Catalyzed Semi-Hydrogenation of Alkynes. *ChemCatChem* **2020**, *12* (21), 5359–5363. https://doi.org/10.1002/cctc.202000994.

(7) Das, M.; OShea, D. F. Z-Stereoselective Aza-Peterson Olefinations with Bis(Trimethylsilane) Reagents and Sulfinyl Imines. *Org. Lett.* **2016**, *18* (2), 336–339. https://doi.org/10.1021/acs.orglett.5b03519.

(8) Dong, D. J.; Li, H. H.; Tian, S. K. A Highly Tunable Stereoselective Olefination of Semistabilized Triphenylphosphonium Ylides with N -Sulfonyl Imines. *J. Am. Chem. Soc.* **2010**, *132* (14), 5018–5020. https://doi.org/10.1021/ja910238f.

(9) Brzozowska, A.; Azofra, L. M.; Zubar, V.; Atodiresei, I.; Cavallo, L.; Rueping, M.; El-Sepelgy, O. Highly Chemo- and Stereoselective Transfer Semihydrogenation of Alkynes Catalyzed by a Stable, Well-Defined Manganese(II) Complex. *ACS Catal.* **2018**, *8* (5), 4103–4109. https://doi.org/10.1021/acscatal.8b00983.

(10) Byrne, P. A.; Gilheany, D. G. Unequivocal Experimental Evidence for a Unified Lithium Salt-Free Wittig Reaction Mechanism for All Phosphonium Ylide Types: Reactions with β-Heteroatom-Substituted Aldehydes Are Consistently Selective for Cis-Oxaphosphetane-Derived Products. *J. Am. Chem. Soc.* **2012**, *134* (22), 9225–9239. https://doi.org/10.1021/ja300943z.

(11) Kvaran, Á.; Konrásson, Á. E.; Evans, C.; Geirsson, J. K. F. 1H NMR and UV-Vis Spectroscopy of Chlorine Substituted Stilbenes: Conformational Studies. *J. Mol. Struct.* **2000**, *553* (1–3), 79–90. https://doi.org/10.1016/S0022-2860(00)00546-9.

(12) Chen, F.; Zhang, X. Pd-Catalyzed Highly Regio- and Stereocontrolled Direct Alkenylation of Electron-Deficient Polyfluoroarenes. *Chem. Lett.* **2011**, *40* (9), 978–979. https://doi.org/10.1246/cl.2011.978.

(13) Boger, D. L.; Sakya, S. M.; Yohannes, D. Total Synthesis of Combretastatin D-2: Intramolecular Ullmann Macrocyclization Reaction. *J. Org. Chem.* **1991**, *56* (13), 4204–4207. https://doi.org/10.1021/jo00013a024.

(14) Singh, S. P.; O'Donnell, J. S.; Schwan, A. L. Nucleophilic Attack of 2-Sulfinyl

Acrylates: A Mild and General Approach to Sulfenic Acid Anions. *Org. Biomol. Chem.* **2010**, *8* (7), 1712–1717. https://doi.org/10.1039/b917217c.

(15) O’Brien, J. G. K.; Chintala, S. R.; Fox, J. M. Stereoselective Synthesis of Bicyclo[6.1.0]Nonene Precursors of the Bioorthogonal Reagents s-TCO and BCN. *J. Org. Chem.* **2018**, *83* (14), 7500–7503. https://doi.org/10.1021/acs.joc.7b02329.

(16) Tortajada, A.; Mestres, R.; Iglesias-Arteaga, M. A. Synthesis of (Z)-5-Decenol and (Z)-5-Decenyl Acetate, Components of the Sex Pheromones of a Variety of Lepidoptera. *Synth. Commun.* **2003**, *33* (10), 1809–1814. https://doi.org/10.1081/SCC-120018943.

(17) Nishibayashi, R.; Kurahashi, T.; Matsubara, S. Palladium Porphyrin Catalyzed Hydrogenation of Alkynes: Stereoselective Synthesis of Cis -Alkenes. *Synlett* **2014**, *25* (9), 1287–1290. https://doi.org/10.1055/s-0033-1341240.

(18) Pai, Z. P.; Khlebnikova, T. B.; Mattsat, Y. V.; Parmon, V. N. Catalytic Oxidation of Fatty Acids. I. Epoxidation of Unsaturated Fatty Acids. *React. Kinet. Catal. Lett.* **2009**, *98* (1), 1–8. https://doi.org/10.1007/s11144-009-0069-2.

(19) Dey, S.; Karabal, P. U.; Sudalai, A. Concise Enantioselective Synthesis of Naturally Active (S)-3-Hydroxypiperidine. *Synth. Commun.* **2015**, *45* (13), 1559–1565. https://doi.org/10.1080/00397911.2015.1033428.

(20) Iwasaki, T.; Miyata, Y.; Akimoto, R.; Fujii, Y.; Kuniyasu, H.; Kambe, N. Diarylrhodates as Promising Active Catalysts for the Arylation of Vinyl Ethers with Grignard Reagents. *J. Am. Chem. Soc.* **2014**, *136* (26), 9260–9263. https://doi.org/10.1021/ja5043534.

(21) Huang, W.; Xu, J. In Situ Generation of Formaldehyde and Triphenylphosphine from (Hydroxymethyl)Triphenylphosphonium and Its Application in Wittig Olefination. *Synth. Commun.* **2015**, *45* (15), 1777–1782. https://doi.org/10.1080/00397911.2015.1043019.

(22) Kang, S. K.; Lee, H. W.; Kim, J. S.; Choi, S. C. Palladium-Catalyzed Cross-Coupling of Organostannanes with Iodanes. *Tetrahedron Lett.* **1996**, *37* (21), 3723–3726. https://doi.org/10.1016/0040-4039(96)00669-7.

(23) Yabe, Y.; Yamada, T.; Nagata, S.; Sawama, Y.; Monguchi, Y.; Sajiki, H. Development of a Palladium on Boron Nitride Catalyst and Its Application to the Semihydrogenation of Alkynes. *Adv. Synth. Catal.* **2012**, *354* (7), 1264–1268. https://doi.org/10.1002/adsc.201100936.
